# Discovery of genomic regions and candidate genes controlling shelling percentage using QTL‐seq approach in cultivated peanut (*Arachis hypogaea* L.)

**DOI:** 10.1111/pbi.13050

**Published:** 2019-01-30

**Authors:** Huaiyong Luo, Manish K. Pandey, Aamir W. Khan, Jianbin Guo, Bei Wu, Yan Cai, Li Huang, Xiaojing Zhou, Yuning Chen, Weigang Chen, Nian Liu, Yong Lei, Boshou Liao, Rajeev K. Varshney, Huifang Jiang

**Affiliations:** ^1^ Key Laboratory of Biology and Genetic Improvement of Oil Crops Ministry of Agriculture Oil Crops Research Institute of the Chinese Academy of Agricultural Sciences Wuhan China; ^2^ Center of Excellence in Genomics & Systems Biology (CEGSB) International Crops Research Institute for the Semi‐Arid Tropics (ICRISAT) Hyderabad India

**Keywords:** peanut, shelling percentage, QTL‐seq, genomic regions, candidate genes

## Abstract

Cultivated peanut (*Arachis hypogaea* L.) is an important grain legume providing high‐quality cooking oil, rich proteins and other nutrients. Shelling percentage (SP) is the 2nd most important agronomic trait after pod yield and this trait significantly affects the economic value of peanut in the market. Deployment of diagnostic markers through genomics‐assisted breeding (GAB) can accelerate the process of developing improved varieties with enhanced SP. In this context, we deployed the QTL‐seq approach to identify genomic regions and candidate genes controlling SP in a recombinant inbred line population (Yuanza 9102 × Xuzhou 68‐4). Four libraries (two parents and two extreme bulks) were constructed and sequenced, generating 456.89–790.32 million reads and achieving 91.85%–93.18% genome coverage and 14.04–21.37 mean read depth. Comprehensive analysis of two sets of data (Yuanza 9102/two bulks and Xuzhou 68‐4/two bulks) using the QTL‐seq pipeline resulted in discovery of two overlapped genomic regions (2.75 Mb on A09 and 1.1 Mb on B02). Nine candidate genes affected by 10 SNPs with non‐synonymous effects or in UTRs were identified in these regions for SP. Cost‐effective KASP (Kompetitive Allele‐Specific PCR) markers were developed for one SNP from A09 and three SNPs from B02 chromosome. Genotyping of the mapping population with these newly developed KASP markers confirmed the major control and stable expressions of these genomic regions across five environments. The identified candidate genomic regions and genes for SP further provide opportunity for gene cloning and deployment of diagnostic markers in molecular breeding for achieving high SP in improved varieties.

## Introduction

**Figure 1:**
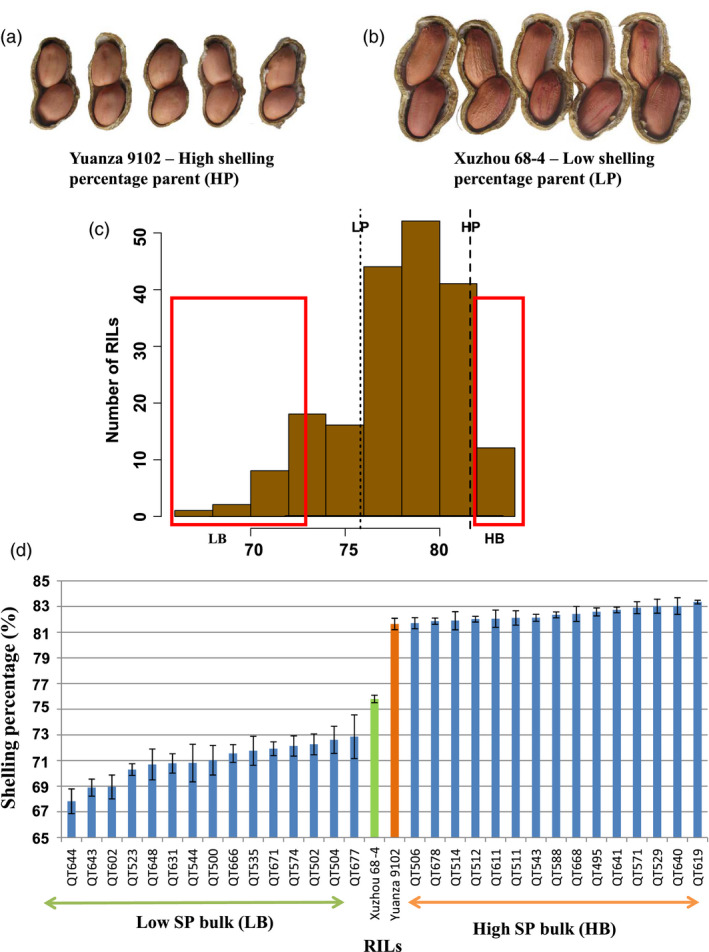
Construction of the extreme bulks for shelling percentage. (a) Xuzhou 68‐4: low value parent for shelling percentage; (b) Yuanza 9102: high value parent for shelling percentage; (c) Frequency distribution for mean values of shelling percentage in RIL population. These mean values were calculated based four seasons in Wuhan city, that is Wuhan2013, Wuhan2014, Wuhan2015 and Wuhan2016; (d) Phenotypic variability among the RILs selected for development of extreme bulks for shelling percentage. Based on the four seasons phenotyping of RIL population, 15 high shelling percentage RILs and 15 low shelling percentage RILs were used to constitute low and high bulks.

The cultivated peanut, *Arachis hypogaea* L., is an important oilseeds crop grown in >100 countries and is consumed worldwide in form of high‐quality cooking oil, edible nut, peanut butter and candy. During 2016, the annual production of peanut pods (with shell) was more than 43.98 million tonnes throughout the world (FAOSTAT). Peanut is an allotetraploid grain legume (AABB, 2*n* = 4*x* = 40) with genome size of ~2.7 Gb (Kochert *et al*., 1996). It is also known as the groundnut due to underground pod development. Peanut pod has two parts: kernel and shell (Figure 1). During post‐harvested processing, pods are passed through peanut shelling machines to collect kernels (seeds) which provide rich nutrients such as high‐quality oil, proteins and a variety of healthy bioactive compounds for human (Davis and Dean, 2016). As by‐product, shells cannot be effectively and economically utilized and may create waste disposal problem around growing or processing areas (Zhao *et al*., 2012). Therefore, shelling percentage (SP), a ratio in percentage of the weight of kernels to the weight of pods, is an important economic trait in peanut production. High SP varieties provide more kernels for human nutrients and produce fewer by‐products than low SP varieties. In recent studies, significant variations for SP were found among peanut varieties, for example ranging from 59.9% to 81.0% in the Chinese core collection (Jiang *et al*., 2013) and from 45.3% to 73.1% in the 256 accessions evaluated in Mediterranean Basin (Yol *et al*., 2018), suggesting that there is a great potential in the enhancement of SP through breeding.

Genomics‐assisted breeding (GAB) can accelerate the process of developing new varieties with improved traits of interest through precise selection of favourable alleles across generations (Varshney *et al*., 2005). However, diagnostic markers of the targeted traits are the prerequisite for deploying GAB and currently no such diagnostic marker is available for SP. The identification of linked markers to major and stable quantitative traits loci (QTLs) for SP is required to develop improved peanut varieties. Limited efforts were made to identify QTLs associated with SP in cultivated peanut, however, none of them could develop markers for use in breeding (Faye *et al*., 2015; Huang *et al*., 2015). Also these QTLs were detected in single environment, hence, do not provide information on their stability and consistency. Association analysis identified one SSR allele linked with SP in three field trials but had only 1.49%–2.98% phenotypic variation explained (PVE) (Jiang *et al*., 2014) which cannot be exploited in GAB. Recent study from our research group identified two major and consistent QTLs for SP, that is *cqSPA09* (10.47%–17.01% PVE) and *cqSPB02* (8.01%–11.20% PVE), in a recombinant inbred line (RIL) population (Yuanza 9102 × Xuzhou 68‐4) across four consecutive years in Wuhan, China (Luo *et al*., 2017). Despite providing idea on genomic regions, none of these studies provided precise localization in the peanut genome, which hampered further candidate gene discovery and diagnostic marker development for SP.

Sequencing‐based trait mapping approaches facilitate faster discovery of genomic regions and candidate genes for target traits (Pandey *et al*., 2016).The QTL‐seq approach (Takagi *et al*., 2013) is one of the successful sequencing‐based mapping approaches which deals with sequencing of pooled samples for RILs with extreme phenotypes and parental genotypes. As a result, the approach rapidly locates the candidate genomic regions and underlying genes for QTLs in plants with reference genomes. This approach is cost‐effective as sample number is less for sequencing and facilitates development of cost‐effective markers in less time for deployment in GAB. This approach has been deployed in several crops including rice (Wambugu *et al*., 2018), cucumber (Lu *et al*., 2014), tomato (Illa‐Berenguer *et al*., 2015), rapeseed (Hua *et al*., 2016), chickpea (Singh *et al*., 2016a), pigeonpea (Singh *et al*., 2016b), peanut (Pandey *et al*., 2017) and soybean (Zhong *et al*., 2018), and proved to be very successful in RIL populations with phenotyping data generated in multiple environments. Since the reference genome sequence of cultivated peanut is still unpublished, the available reference genome sequences of its two diploid ancestors, *A. duranensis* (AA, 2*n* = 2*x* = 20) and *A. ipaensis* (BB, 2*n* = 2*x* = 20) (Bertioli *et al*., 2016) provided a foundation to find candidate genes and SNPs present in QTL regions. In summary, this study reports the deployment of QTL‐seq approach that successfully located the genomic regions on the peanut genome and facilitated discovery of candidate genes and marker development for SP to use in GAB for faster development of new varieties.

## Results

### Construction of extreme bulks for shelling percentage

**Figure 2:**
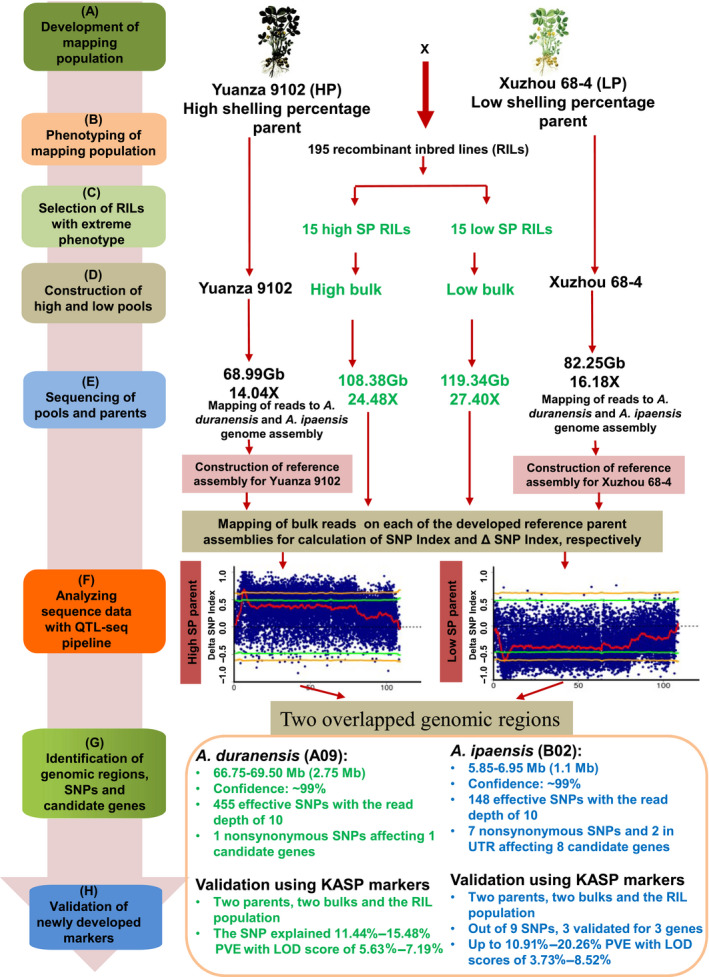
QTL‐seq approach used for trait mapping in peanut for shelling percentage.

The RIL population (Yuanza 9102 × Xuzhou 68‐4) had high phenotypic variability for SP (Figures 1 and S1). Based on the phenotyping data generated in Wuhan in four consecutive years (Luo *et al*., 2017), the SP of Yuanza 9102 was 81.65 ± 0.44%, whereas that of Xuzhou 68‐4 was 75.81 ± 0.29% (Table S1). The SP of the RIL population showed continuous distributions skewed towards higher values in all of the four environments (Figure S2), ranging from 67.83% to 83.36% on average. Therefore, 15 extreme RILs with high SP (81.71%–83.36%) and 15 extreme RILs with low SP (67.83%–72.87%) were selected to construct the two bulks (Figure 1, Table S1), that is high SP bulk (HB) and low SP bulk (LB). The whole‐genome resequencing (WGRS) data were generated for these extreme bulks in addition to parental genotypes followed by their analysis using the QTL‐seq pipeline as shown in Figure 2.

### Whole‐genome resequencing, mapping of reads and identification of SNPs

WGRS data were generated for Xuzhou 68‐4, Yuanza 9102, HB and LB samples using the Illumina NovaSeq. A total of 544.70 million reads for Xuzhou 68‐4, 456.89 million reads for Yuanza 9102, 790.32 million reads for HB and 717.72 million reads for LB were generated (Tables 1 and S2). The maximum sequencing data were obtained for HB (119.34 Gb) followed by LB (108.38 Gb), Xuzhou 68‐4 (82.25 Gb) and Yuanza 9102 (68.99 Gb).

**Table 1:** Summary of shelling percentage and Illumina sequencing of parental lines and bulks

Sample	Mean shelling percentage	Number of reads generated	Total bases	Genome coverage (%)	Mean depth (*X*)
Xuzhou 68‐4^a^	75.81	544 700 200	82 249 730 200	92.61	16.18
LB^b^	70.96	717 723 902	108 376 309 202	93.12	20.96
HB^b^	82.42	790 320 814	119 338 442 914	93.18	21.37
Yuanza 9102^a^	81.65	456 893 014	68 990 845 114	91.85	14.04
LB^c^	70.96	717 723 902	108 376 309 202	93.06	20.96
HB^c^	82.42	790 320 814	119 338 442 914	93.12	21.37

a The short reads of parental lines were aligned to the publicly available genome of diploid progenitors *A. duranensis* and *A. ipaensis* (PeanutBase: http://peanutbase.org).

b The short reads of the extreme bulks were aligned to the Xuzhou ‘reference sequence’ developed by replacement of SNPs between Xuzhou 68‐4 and diploid progenitors.

c The short reads of bulks were aligned to the Yuanza ‘reference sequence’ developed by replacement of SNPs between Yuanza 9102 and diploid progenitors.

The reads of Xuzhou 68‐4 were aligned to the genome sequences of *A. duranensis* and *A. ipaensis*, and resulted in 92.61% genome coverage and 16.18 mean read depth. A reference‐guided assembly was then generated for the male parent Xuzhou 68‐4 and referred as Xuzhou assembly hereafter (Figure 2). The reads of the two extreme bulks were mapped to the Xuzhou assembly, and achieved 93.18% mapping coverage and 21.37 mean read depth for HB, whereas 93.12% coverage and 20.96 mean read depth for LB (Tables 1 and S2). The comprehensive sequence analysis between the high and low bulks identified 172 715 genome‐wide SNPs (Table S3).

**Figure 3:**
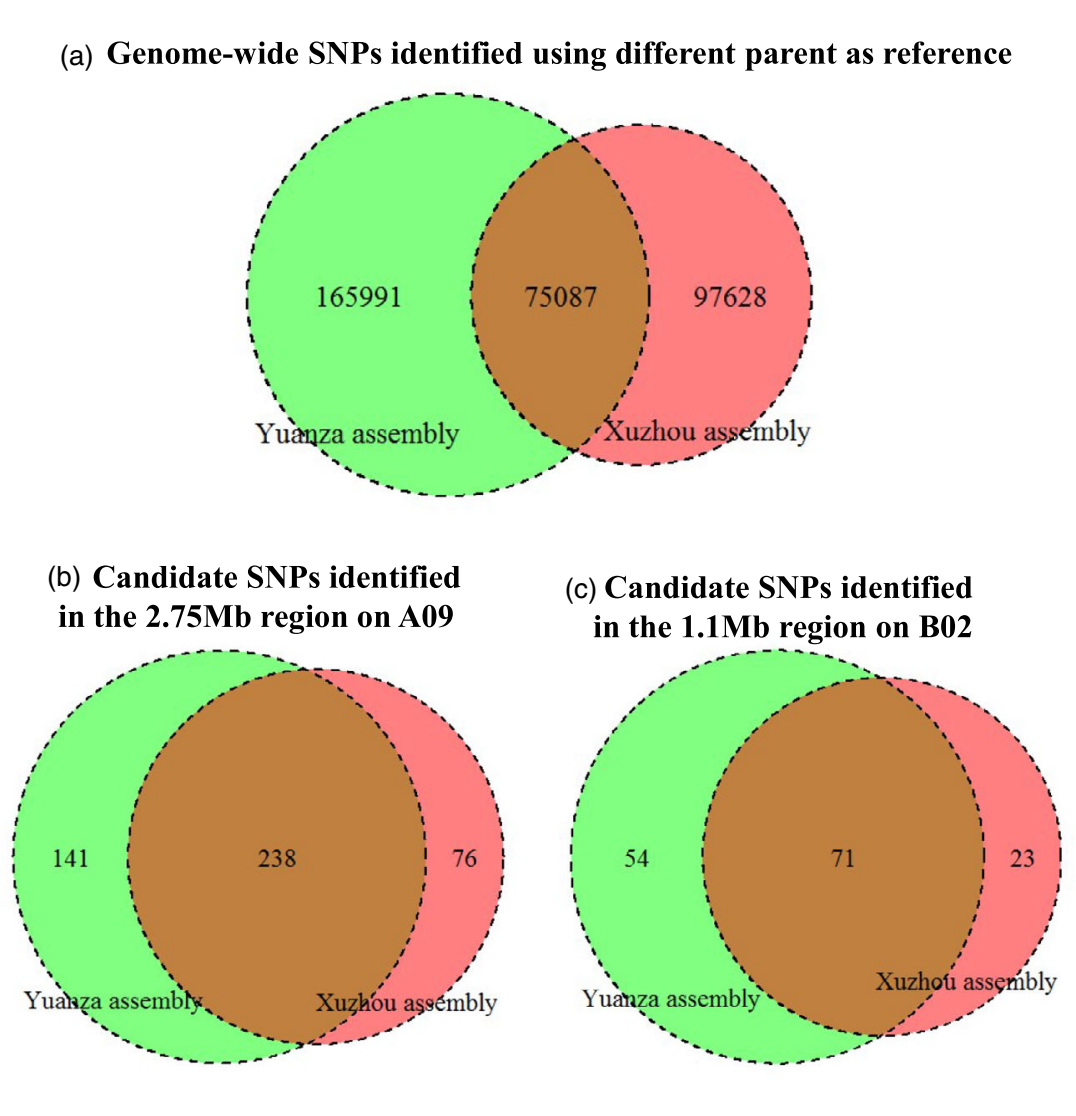
SNPs identified using the QTL‐seq approach with different parent as reference. Numbers of SNPs identified with the Xuzhou assembly as reference (Red) and numbers of SNPs identified with the Yuanza assembly as reference (Green).

Similarly, the reads of Yuanza 9102 were aligned to the genome sequences of *A. duranensis* and *A. ipaensis*, and resulted in 91.85% genome coverage and 14.04 mean average read depth. A reference‐guided assembly was then generated for the female parent Yuanza 9102 and referred as Yuanza assembly hereafter (Figure 2). The reads of the two extreme bulks were mapped to the Yuanza assembly and achieved 93.12% coverage and 21.37 mean read depth for HB, whereas 93.06% coverage and 20.96 mean read depth for LB (Tables 1 and S2). The comprehensive sequence analysis between the high and low bulks identified 241 078 genome‐wide SNPs (Table S3). This resulted in discovery of a common set of 75 087 SNPs when either of the parents’ assembly was used as reference (Figure 3).

### Candidate genomic regions for shelling percentage

**Figure 4:**
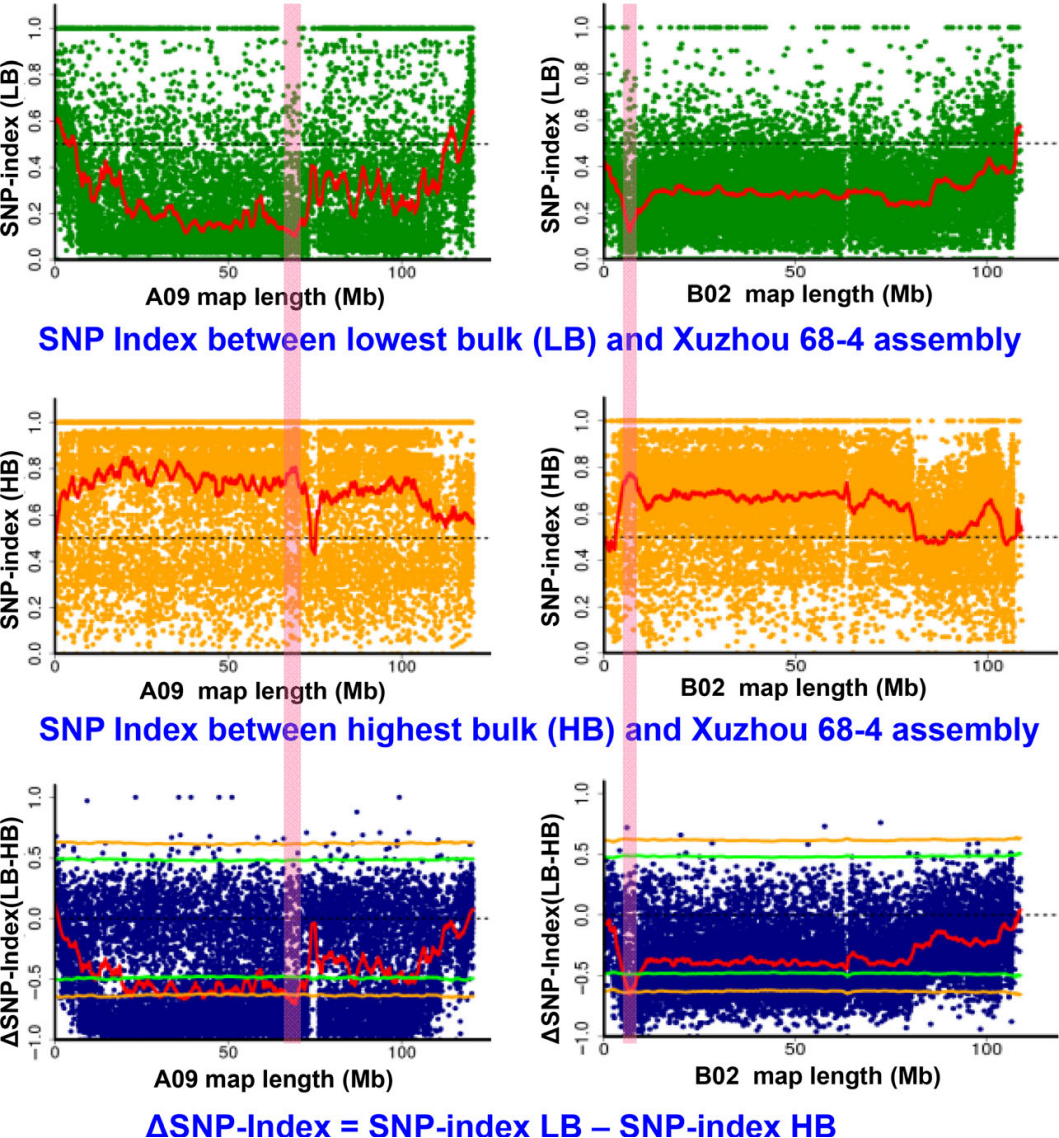
QTL‐seq approach for mapping genomic regions controlling shelling percentage using the Xuzhou 68‐4 as reference parent. SNP index plot between low bulk and Xuzhou assembly (top), high bulk and Xuzhou assembly (middle) and ΔSNP index plot (bottom) of pseudomolecule A09 and B02 with statistical confidence interval under the null hypothesis of no QTLs (orange, *P* < 0.01 and green *P* < 0.05). The significant genomic region is shaded (66.70–69.90 Mb on A09 and 5.65–6.95 Mb on B02).

With the Xuzhou assembly as reference, the SNP‐index of the genome‐wide SNPs was calculated for each bulk (Figure S3). The ΔSNP‐index was then calculated by subtracting SNP‐index of HB from SNP‐index of LB. If more alleles of one parent than the other were presented in the two extreme bulks, the SNP‐index will significantly deviate from 0.5 and the ΔSNP‐index will significantly deviate from 0 (zero). Based on the sliding window analysis for SNP‐index and ΔSNP‐index plots, a 3.20 Mb (66.70–69.90 Mb) interval on chromosome A09 and a 1.30 Mb (5.65–6.95 Mb) interval on chromosome B02 were identified for SP at a statistical confidence of *P* < 0.01 (Figures 4 and S4–S6). The ΔSNP‐index of the two genomic regions were negative, indicating that more alleles were from the reference parent Xuzhou 68‐4 in the low SP bulk but from non‐reference parent Yuanza 9102 in the high SP bulk.

**Figure 5:**
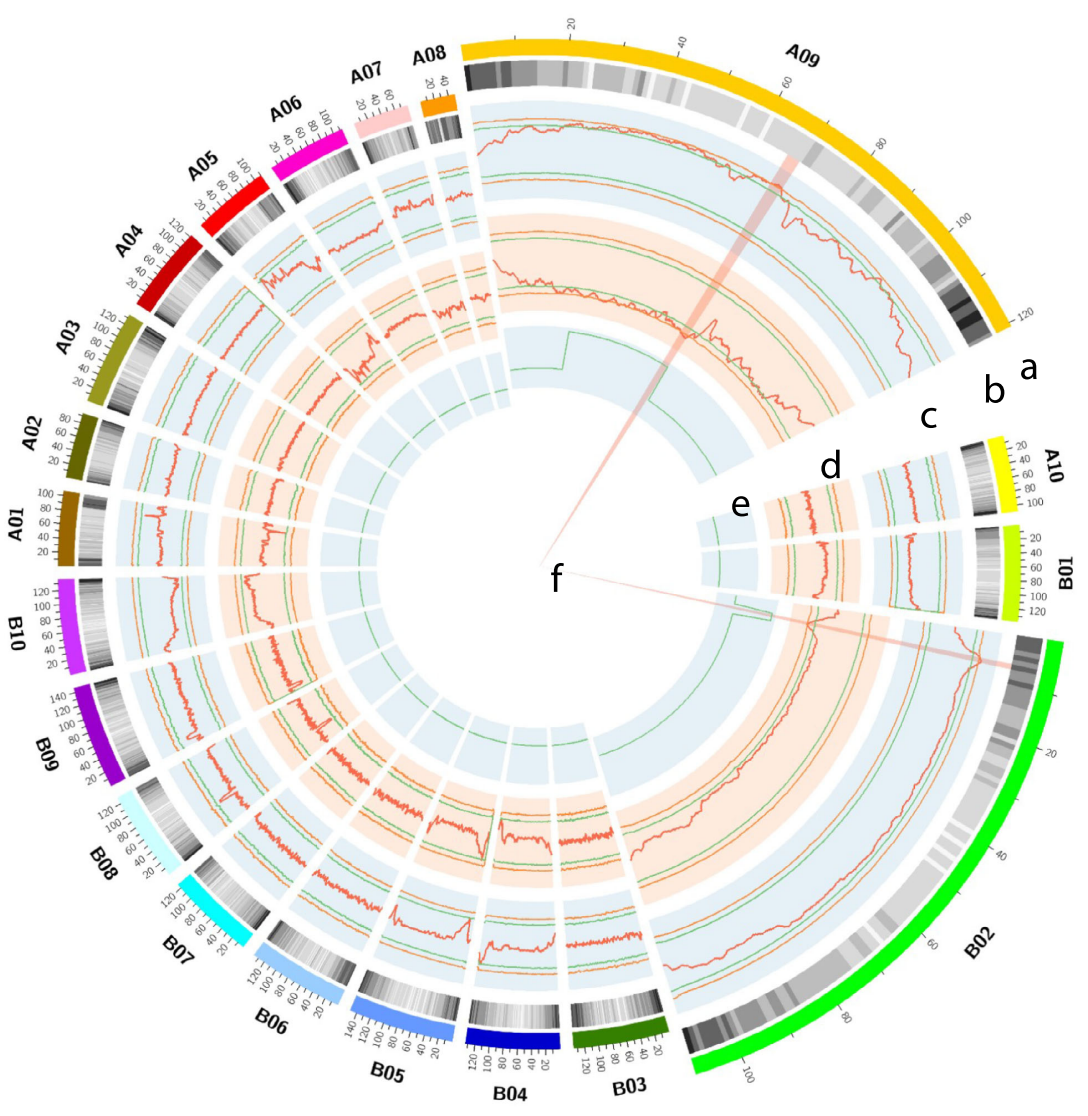
Co‐localization of QTLs from traditional and QTL‐seq approach for shelling percentage. (a) Psuedomolecules of reference genome *Arachis duranensis* and *A. ipaensis* (b) Genomewide density of annotated genes. (c) ΔSNP index plot using the Yuanza assembly as reference, from outside to inside: upper probability values at 99% confidence, upper probability values at 95% confidence, ΔSNP index, lower probability values at 95% confidence, lower probability values at 99% confidence. (d) ΔSNP index plot using the Xuzhou assembly as reference, from outside to inside: upper probability values at 99% confidence, upper probability values at 95% confidence, ΔSNP index, lower probability values at 95% confidence, lower probability values at 99% confidence. (e) Physical position of earlier mapped QTL (Luo *et al*., 2017) for shelling percentage through traditional mapping approach. The physical position of QTL was estimated through blast the flanking primers into the *A. duranensis* and *A. ipaensis* genome. (f) Common genomic positions observed through both approaches.

Similarly, a 2.75 Mb (66.75–69.50 Mb) interval on A09 and a 1.50 Mb (5.85–7.35 Mb) interval on B02 were identified at *P* < 0.01 level, using the Yuanza assembly as reference (Figures S7–S9). The ΔSNP‐index of the two genomic regions were positive, indicating that more alleles were from the non‐reference parent Xuzhou 68‐4 in the low SP bulk but from reference parent Yuanza 9102 in the high SP bulk. These intervals were significantly overlapped with the genomic regions identified with the Xuzhou assembly (Table 2). Therefore, the overlapped regions were selected as the candidate regions for SP, that is, 66.75–69.50 Mb on A09 and 5.65–6.95 Mb on B02 (Figure 5, Table 2).

**Table 2:** Genomic regions identified for shelling percentage

Reference assembly	Chr	Genomic region (Mb)	Length (Mb)	ΔSNP‐index	U99	L99	Allele source
Xuzhou	A09	66.70–69.90	3.20	−0.71	0.62	−0.63	Yuanza 9102
	B02	5.65–6.95	1.30	−0.66	0.62	−0.63	Yuanza 9102
Yuanza	A09	66.75–69.50	2.75	0.69	0.63	−0.61	Yuanza 9102
	B02	5.85–7.35	1.50	0.69	0.63	−0.61	Yuanza 9102
Overlapped	A09	66.75–69.50	2.75				Yuanza 9102
	B02	5.85–6.95	1.10				Yuanza 9102

### Candidate SNPs and putative candidate genes in the genomic regions for shelling percentage

The genomic region spanning 2.75 Mb on chromosome A09 had 455 effective SNPs with read depth of ≥10, SNP‐index significantly deviated from 0.5 and ΔSNP‐index higher than the statistical confidence at *P* < 0.01 (Figure 3, Tables S4 and S5). Of the 455 SNPs, 454 SNPs were intergenic and one was non‐synonymous. The non‐synonymous SNP could be identified using both parents as reference and affected the candidate gene encoding histone‐lysine N‐methyltransferase SUVR2‐like isoform X2 (*Aradu.A5UR9*) (Table 3).

**Table 3:** Identification of SNPs in putative candidate genes for shelling percentage

Chr	Gene	Position (bp)	Xuzhou 68‐4 base	Yuanza 9102 base	Low bulk base	High bulk base	SNP effect	Amino acid change	Function	Reference assembly
A09	*Aradu.A5UR9*	66949737	G	A	G	A	Missense	Arg575Trp	Histone‐lysine N‐methyltransferase SUVR2‐like isoform X2	Both
B02	*Araip.G7SZ3*	5861413	T	C	T	C	Stop_gain	Gln52*	Unknown protein	Yuanza
	*Araip.HEW90*	6001749	C	G	C	G	3′UTR		Unknown protein	Yuanza
	*Araip.R30RS*	6121202	A	G	A	G	Missense	Thr107Ile	Serine/threonine‐protein phosphatase 7 long form homolog	Both
	*Araip.N485E*	6148445	G	A	G	A	Missense	Arg154Cys	Serine/threonine‐protein phosphatase 7 long form homolog	Yuanza
	*Araip.J1S7B*	6155951	G	T	G	T	Missense	Arg1068Met	Vacuolar protein sorting‐associated protein 8 homolog	Yuanza
	*Araip.VZ4DX*	6470236	C	G	C	G	Missense	Ala112Gly	Ulp1 protease family carboxy‐terminal domain protein	Xuzhou
		6471046	T	C	T	C	Missense	Ser236Phe		Both
	*Araip.Y03J3*	6770282	G	A	G	A	Missense	Gly156Arg	Unknown protein	Yuanza
	*Araip.X1ALQ*	6776001	C	T	C	T	5′UTR		Unknown protein	Both

The genomic region spanning 1.1 Mb on chromosome B02 had 148 effective SNPs with read depth of ≥10, SNP‐index significantly deviated from 0.5 in addition to ΔSNP‐index higher than the statistical confidence at *P* < 0.01 (Figure 3, Tables S4 and S5). Of the 148 SNPs, 134 SNPs were intergenic and 14 SNPs were genic, including four intronic, seven non‐synonymous (one resulted in stop gained), one synonymous and two in UTRs. The seven non‐synonymous SNPs and the two SNPs in UTRs affected eight candidate genes encoding four unknown proteins (*Araip.G7SZ3*,* Araip.HEW90*,* Araip.Y03J3* and *Araip.X1ALQ*), two serine/threonine‐protein phosphatase homologs (*Araip.R30RS* and *Araip.N485E*), one vacuolar protein sorting‐associated protein (*Araip.J1S7B*) and one Ulp1 protease family carboxy‐terminal domain protein (*Araip.VZ4DX*) (Table 3).

### Validation of identified genomic regions

To validate the genomic regions identified for SP, 10 SNPs (One SNP from A09 and nine SNPs from B02) were targeted for development of KASP markers. Primers were successfully developed for the SNP at position 66949737 on chromosome A09. Of the nine SNPs on chromosome B02, primers were developed for three SNPs, whereas no primers could be designed for remaining six SNPs (Table 4, Figure S10). The RIL population (Yuanza 9102 and Xuzhou 68‐4) in F_9_ generation were genotyped with the four KASP markers (Table S6).

**Table 4:** List of KASP markers developed for shelling percentage

ID	Primer type	Primer sequence^a^	Allele	Parent
Aradu.A09_66949737	Allele‐specific (HEX)	TCTTAAACCTCGTTGCACTACCCG	G	Xuzhou 68‐4
	Allele‐specific (FAM)	ATCTTAAACCTCGTTGCACTACCCA	A	Yuanza 9102
	Common	TGCCTGAAGAATGCAAGGGGCATTT		
Araip_B02_6155951	Allele‐specific (FAM)	AGTACTTGAGGTAAACCAGCATCC	G	Xuzhou 68‐4
	Allele‐specific (HEX)	CAGTACTTGAGGTAAACCAGCATCA	T	Yuanza 9102
	Common	TACTTCTTTTGCCTCTTGTTTTAACAGGTA		
Araip_B02_6770282	Allele‐specific (FAM)	GTGGTGCTTTATGCAGCCTCG	G	Xuzhou 68‐4
	Allele‐specific (HEX)	CGTGGTGCTTTATGCAGCCTCA	A	Yuanza 9102
	Common	GCACAGGGTTGCGCGGAGTT		
Araip_B02_6776001	Allele‐specific (FAM)	GATTTACTATGTGTTTCACTAAATCGAATC	C	Xuzhou 68‐4
	Allele‐specific (HEX)	TGATTTACTATGTGTTTCACTAAATCGAATT	T	Yuanza 9102
	Common	GCAACGTCAAAGTCCCAAACCACAT		

Sequences of the allele‐specific primers do not include the tail sequences that interact with the fluor‐labelled oligos in the KASP reaction mix.

To test the stability of the identified QTLs, the phenotyping data of the RIL population in F_9_ generation was generated in another geographical location in 2017 (Yangluo2017) (Table S7), in addition to the phenotyping data used to construct extreme bulks. The phenotypic distribution in the Yangluo2017 environment was continuous and had two peaks (Figure S2). Single‐marker QTL analysis with the phenotyping data in the five environments showed high significances (*P* < 0.001) for all of the four KASP markers (Table 5). The marker, Aradu_A09_66949737, on chromosome A09 explained high phenotypic variation of 11.44%–15.48% with LOD (Likelihood of odds) scores of 5.63–7.19 across five environments. The genotyping data of the three KASP markers (Araip_B02_6155951, Araip_B02_6770282 and Araip_B02_6776001) on chromosome B02 were used to estimate genetic distances, and a map length of 2.76 cm was obtained. Among the three candidate markers on chromosome B02, Araip_B02_6776001 explained highest phenotypic variation 10.91%–20.26% with LOD scores of 3.73–8.52, whereas Araip_B02_6155951 explained 8.18%–19.20% with LOD scores of 3.54–8.42. These marker‐trait associations confirmed the identified genomic regions harbouring candidate genes for two major and stable QTLs for SP.

**Table 5:** Single‐marker analysis for shelling percentage

Marker	Environment	PVE (%)	LOD	*P*‐value
Aradu_A09_66949737	Yangluo2017	11.44	4.92	2.23E‐06
	Wuhan2016	13.50	6.16	1.21E‐07
	Wuhan2015	15.48	7.19	1.10E‐08
	Wuhan2014	12.14	5.63	4.21E‐07
	Wuhan2013	13.78	6.41	6.60E‐08
Araip_B02_6155951	Yangluo2017	19.20	8.42	<1.0E‐09
	Wuhan2016	14.09	6.31	8.40E‐08
	Wuhan2015	14.90	6.90	2.10E‐08
	Wuhan2014	17.20	8.25	1.00E‐09
	Wuhan2013	8.18	3.54	4.81E‐05
Araip_B02_6770282	Yangluo2017	17.46	9.31	2.00E‐09
	Wuhan2016	13.29	6.08	1.46E‐07
	Wuhan2015	14.49	6.37	7.30E‐08
	Wuhan2014	17.04	7.98	2.00E‐09
	Wuhan2013	9.13	3.83	3.00E‐05
Araip_B02_6776001	Yangluo2017	20.26	8.51	2.00E‐09
	Wuhan2016	16.31	6.44	6.20E‐08
	Wuhan2015	17.24	6.91	2.10E‐08
	Wuhan2014	19.60	8.52	<1.0E‐09
	Wuhan2013	10.91	3.73	3.78E‐05

PVE, phenotypic variation explained.

To evaluate the combined effect of the identified QTLs, RILs were classified into four groups using the four KASP markers as diagnostic markers. The alleles of the identified QTLs on A09 and B02 from Yuanza 9102 were designated as ‘AA’ and ‘BB’, respectively, whereas those from Xuzhou 68‐4 were designated as ‘aa’ and ‘bb’. RILs with the AABB genotype showed significant higher SP than the other three genotypes (aaBB, AAbb or aabb) in all five environments (Figure S11, Table S8). The average SP of RILs with the aaBB genotype was slightly higher than that with the AAbb genotype but not significant. Furthermore, RILs with the aaBB or AAbb had significant higher shelling percentages than those with the aabb genotype.

In addition, the four KASP markers were also genotyped in diverse panel consisting of 337 cultivars whose shelling percentages successfully evaluated in Wuhan in 2016 and Nanchong in 2017. Eight cultivars showed the AA genotype and their shelling percentages were significantly higher than the others’ (Figure S12a). A total of 61 cultivars showed the BB genotype, however, their shelling percentages were significantly lower than the others’ on average (Figure S12b). Six cultivars showed the AABB genotype, and their shelling percentages were significantly higher than the 274 cultivars with aabb genotype (Figure S12c).

## Discussion

The genetic mapping in cultivated peanut started in 2009 with only 135 SSR loci (Varshney *et al*., 2009a). The last decade witnessed development and availability of genomic and genetic resources including reference genomes of diploid progenitors and several thousands of genetic markers in peanut (Bertioli *et al*., 2016; Chen *et al*., 2016; Luo *et al*., 2017; Shirasawa *et al*., 2012). The genetic markers were widely used in the QTL mapping of important traits such as yield, kernel, oil quality, disease resistance and drought tolerance (Vishwakarma *et al*., 2017). However, genotyping of these markers in mapping population is laborious and time‐consuming, and also do not lead to discovery of candidate genes and tightly linked markers. Additionally, low level of polymorphism poses a great challenge in developing high‐density genetic maps and conducting high‐resolution genetic mapping (Luo *et al*., 2017). In the recent past, NGS‐based technologies were deployed to overcome these issues because of the affordable cost of sequencing and large number of genome‐wide SNPs identified by NGS‐based technologies for trait mapping and molecular breeding (Varshney *et al*., 2009b; Zhou *et al*., 2014). The recently completed genome sequences of the diploid ancestors of cultivated peanut were reported to be highly similar to cultivated peanut's A and B subgenomes (Bertioli *et al*., 2016), which may due to its short evolutionary history after two diploid genomes merged ~3500–4500 years ago (Kochert *et al*., 1996). The genome sequences of the diploid ancestors were used as reference and proved to be successful in rapid trait mapping by NGS‐based technologies in peanut (Agarwal *et al*., 2018; Bertioli *et al*., 2016; Pandey *et al*., 2017; Wang *et al*., 2018). One of such approach namely QTL‐seq only requires whole‐genome resequencing of extreme bulks and parent of mapping population, making it cost effective (Takagi *et al*., 2013). This approach has been proved to be very successful with RIL populations in several crops including rice (Takagi *et al*., 2013), peanut (Pandey *et al*., 2017), cucumber (Wei *et al*., 2016), chickpea (Singh *et al*., 2016a) and pigeonpea (Singh *et al*., 2016b). Therefore, we have applied the QTL‐seq approach, in the present study, to identify genomic regions and candidate genes for the important economic trait namely SP in a RIL population (Yuanza 9102 × Xuzhou 68‐4).

According to the QTL‐seq approach, only one parent was sequenced and used as reference to compare the two extreme bulks (Takagi *et al*., 2013), and recent reports in cucumber (Lu *et al*., 2014), tomato (Illa‐Berenguer *et al*., 2015), rapeseed (Hua *et al*., 2016), chickpea (Singh *et al*., 2016a), pigeonpea (Singh *et al*., 2016b), peanut (Pandey *et al*., 2017), soybean (Zhong *et al*., 2018) and rice (Wambugu *et al*., 2018) used one parent as reference. Whether the choice of different parent would affect the results were not reported. In this study, both parents were sequenced and used together with the same bulks to generate two sets of results in parallel using the default parameters of the QTL‐seq pipeline developed by the Iwate Biotechnology Research Center, Japan (Takagi *et al*., 2013), and this is the main methodological difference between this study and other QTL‐seq reports in different crops. The numbers of identified genome‐wide SNPs showed significantly difference between the two sets of results (Figure 3a). The large differences might due to multiple factors, for example: (i) the existing similarity between A and B subgenomes of the cultivated peanut; (ii) the high percentage of repetitive content of peanut (~64%); (iii) the reference‐guided assembly of parents were constructed based on the genome sequences of both the diploid progenitor species *A. duranensis* and *A. ipaensis*, which might be still distant from the cultivated peanut; and (iv) complexity posed due to complex polyploid genome. These factors might cause different alignment errors and detection of false‐positive SNPs upon using different parent‐based reference genomes for sequence analysis of pooled samples. For example, 22 reads (20C + 2G) of LB were mapped at the 6470236 position of chromosome B02 when Xuzhou 68‐4 was used as reference; however, only eight reads (6C + 2G) of LB were mapped at this position when Yuanza 9102 was used as reference. This SNP was identified when Xuzhou 68‐4 used as reference while was treated as spurious and filtered out when Yuanza 9102 used as reference (Table 3). The validated SNPs Araip_B02_6155951 and Araip_B02_6770282 (Table 4) were filtered out when Xuzhou 68‐4 used as reference because their consensus qualities (18 and 12 respectively) were lower than the default cutoff (20) of the QTL‐seq pipeline. However, in order to address the reason for the large differences, a large‐scale testing needs to be conducted on species with different ploidy levels and on different combinations of internal parameters of the QTL‐pipeline. Nevertheless, such large differences did not affect the identification of genomic regions for SP (Table 2). With different parent as reference, two overlapped genomic regions on A09 (2.75 Mb) and B02 (1.1 Mb) were identified as candidates controlling SP. Notably, some candidate SNPs in these regions were unique to either of the parent‐based reference, that is identified with different parent as reference (Figure 3b,c). Among the four SNPs validated with KASP markers, two SNPs were identified only when the Yuanza 9102 was used as reference (Tables 3 and 4). The present study combined the identified SNPs of the two sets of results thus provided comprehensively identification of genomic region, SNPs and candidate genes for SP in peanut. Therefore, along with the reduction in cost of whole‐genome sequencing, it is worth to sequence both parents together with the extreme bulks to improve the results of QTL‐seq approach especially in polyploid species.

SNPs in a big genomic region on chromosome A09 showed unusual deviation from theoretical values of SNP‐index and ΔSNP‐index (Figure 4), which was not observed on remaining 19 chromosomes (Figures [Link], [Link], [Link], [Link], [Link], [Link]). This phenomenon might be due to reduced recombination rates in this region, and was observed in other QTL‐seq studies in different crops with high quality reference genomes (Singh *et al*., 2016a; Takagi *et al*., 2013; Zhong *et al*., 2018), although the sizes of unusual genomic regions varied. In a high‐density genetic map of peanut (Wang *et al*., 2018), reductions in recombination rates were observed in the middle parts of peanut chromosomes, and these regions might be close to centromeres. Although variations in recombination rate were reported in animals (Dukic *et al*., 2016) and plants (Bauer *et al*., 2013; Salome *et al*., 2012), genetic factors controlling recombination are not well understood and need further studies. In order to identify genomic regions controlling SP with higher probability, we applied the *P* < 0.01 cutoff rather than *P* < 0.05 in the present study. Other small peaks on observed A09 (Figure 4) may not be as confident as the major peak, and they were filtered out based on following consideration: (i) they were less significant than the selected region (Figure 4), which might be the result of recombination suppression; (ii) they were significant (*P* < 0.01) when Xuzhou 68‐4 used as reference (Figures 5a and S6) but not significant (*P* < 0.01) when Yuan 9102 used as reference (Figures 5d and S9). As shown in Figure 2, we selected the overlapped genomic regions of two sets of results as candidates controlling SP, which were validated by KASP markers. This is a benefit of sequencing both parents.

The genomic regions identified with the QTL‐seq approach were robust and more precise than the classical SSR marker‐based QTL mapping. The present study successfully identified two genomic regions responsible for SP with stable expressions across five environments. The genomic region (66.75–69.50 Mb) on chromosome A09 was mapped on the same location with the previously identified major QTL *cqSPA09* through classical SSR marker‐based genetic mapping (Luo *et al*., 2017). This study has also successfully narrowed down the QTL region mapped on A09 from 44 Mb (26–70 Mb, identified through genetic mapping) to 2.75 Mb. Similarly, the genomic region (5.85–6.95 Mb) on chromosome B02 identified in this study also harboured the previously identified major QTL *cqSPB02* through classical SSR marker‐based genetic mapping (Luo *et al*., 2017). This study successfully narrowed down the second genomic region mapped on B02 from 3.18 Mb (5.95–9.13 Mb) to 1.1 Mb. Furthermore, the genomic regions for the other 23 QTLs, reported by Luo *et al*. (2017) with lower PVE and less stable across environments, could not be identified based on the QTL‐seq approach. These results illustrated that the QTL‐seq approach could be successful in identifying candidate regions for major and stable QTLs but not for minor QTLs.

The QTL‐seq approach facilitated discovery of 12 putative genes in the 2.75 Mb genomic region on A09, encoding histone‐lysine N‐methyltransferase SUVR2‐like isoform X2, oxysterol‐binding protein‐related protein, ankyrin repeat family protein, zeta‐carotene desaturase, serine carboxypeptidase‐like, RNA‐binding protein, pentatricopeptide repeat (PPR) superfamily protein and function unknown proteins (Table S9). Notably, there was only one non‐synonymous SNP in this region which affected the candidate gene (*Aradu.A5UR9)* encoding histone‐lysine N‐methyltransferase SUVR2‐like isoform X2, which has been reported to involved in transcriptional gene silencing by RNA‐directed DNA methylation in *Arabidopsis thaliana* (Han *et al*., 2014; Liu *et al*., 2018). The possible role of RNA‐directed DNA methylation in controlling of SP in peanut needs to be further investigated. Similarly, the genomic region identified in 1.1 Mb region on B02 harboured 78 putative genes mainly related to catalytic activity, binding, metabolic process and cellular process (Table S9). Notably, there were seven non‐synonymous SNPs and two SNPs in UTR in this region and they affected eight candidate genes (Table 3). *Araip.R30RS* and *Araip.N485E* were predicted to code for serine‐threonine protein phosphatases, which might be the regulators of signal transduction cascades and has been reported to be crucial factor for correct cell division and differentiation in *A. thaliana* (Uhlken *et al*., 2014). *Araip.J1S7B* would code for vacuolar protein sorting‐associated proteins, which form endosomal the sorting complex required for plant development (Cai *et al*., 2014). *Araip.VZ4DX* might code for Ulp1 protease family protein, which regulate protein desumoylation and flower development (Murtas *et al*., 2003). The SNP Araip_B02_6776001 showed significant association with SP and was found located in the 5′‐UTR of *Araip.X1ALQ* encoding function unknown protein. Therefore, the unknown proteins coded by *Araip.G7SZ3*,* Araip.HEW90*,* Araip.Y03J3* and *Araip.X1ALQ* may possess function in the determination of SP as well. Based on these findings, these genes should be targeted as candidates for fine mapping and gene cloning to reveal the genetic basis of SP in peanut.

The main advantage of the sequencing‐based trait mapping approaches is development of markers in addition to gene discovery for target traits (Pandey *et al*., 2016). The QTL‐seq approach has earlier helped in identification of genomic regions and discovery of candidate genes in addition to development of diagnostic markers for foliar disease resistance in peanut (Pandey *et al*., 2017). The QTL‐seq approach deployed in present study also successfully facilitated development and validation of four KASP markers (for one SNP on A09 and for three SNPs on B02) for used in molecular breeding to improve SP. When these KASP markers were used to screen 337 cultivars, five cultivars possessing both alleles of the identified QTLs from Yuanza 9102 showed high shelling percentages, ranging from 76.78% to 81.42% in two environments. When used in selection programs, genotyping with KASP makers is a cost‐effective approach than SSR markers (Steele *et al*., 2018) in addition to being amenable to high‐throughput genotyping. These results indicated the potential of these KASP markers in tracking the favourable alleles for SP in the breeding programs.

In summary, the QTL‐seq approach is powerful to refine genomic regions and identify candidate genes for major and stable QTLs for SP. Utilization of both parents as reference genome generated more comprehensive information for the exploration of candidate SNPs and genes for traits of interest. The present study successfully not only significantly narrowed down the genomic regions for both QTLs but also identified candidate genes and markers for SP for using in breeding.

## Materials and methods

### Plant materials and phenotyping

A RIL population consisting of 195 lines (F_9_ generation) was developed from the cross Yuanza 9102 × Xuzhou 68‐4 by single seed decent (SSD) method (Luo *et al*., 2017). The shelling percentage ($\text{SP}=\frac{\text{Weight of kernels}}{\text{Weight of pods}}\times 100\%$) of female parent, Yuanza 9102, is significantly higher than that of the male parent, Xuzhou 68‐4 (Figure 1). Phenotyping data for SP of the RIL population were generated in four consecutive years in the experimental field in Wuhan city, China and designated as Wuhan2013, Wuhan2014, Wuhan2015 and Wuhan2016 (Luo *et al*., 2017). To test the stability of the identified QTLs, the RIL population in F_9_ generation and the two parents were planted in another experimental field in the rural area in Yangluo, Wuhan, China in 2017, designated as Yuangluo2017. They were planted and phenotyped as explained in the earlier study (Luo *et al*., 2017). Statistical analysis was performed using IBM SPSS Statistics Version 22 software (Armonk, NY).

### Construction of extreme bulks

Genomic DNA of the RILs in F_9_ generation and the two parents were extracted from the young leaves collected in the Yangluo2017 environment using CTAB method (Doyle, 1990). For developing the extreme bulks, 15 RILs with high SP and 15 RILs with low SP were selected based on the mean phenotypic values of precise phenotyping data obtained in four environments, that is, Wuhan2013, Wuhan2014, Wuhan2015 and Wuhan2016 (Luo *et al*., 2017). High SP bulk (HB) were pooled with same amount of DNA from the 15 selected RILs with high phenotypic values, and similarly low SP bulk (LB) were pooled with same amount of DNA from the 15 selected RILs with low mean phenotypic values.

### Construction of libraries and Illumina sequencing

A total of four Illumina libraries were prepared for the HB and LB bulks mentioned above as well as the two parents, using the NEBNext Ultra II DNA Library Prep Kit for Illumina. For each library, two mg DNA was sheared, end repaired, adapter ligated and separated using 2% agarose gel. The fragments around 350 bp were purified and enriched using the adaptor compatible PCR primers. After size checking with Agilent Technologies 2100 Bioanalyzer (Santa Clara, CA), pair‐ended 150 bp short reads of the four amplified libraries were generated on NovaSeq platform with NovaSeq 6000 S4 Reagent Kit.

### Identification of genomic regions for shelling percentage

The QTL‐seq pipeline (QTL‐seq_framework1.4.4), developed by the Iwate Biotechnology Research Center, Japan (Takagi *et al*., 2013), was downloaded and used for the mapping of QTLs for SP with few modification (Figure 2). The raw sequencing reads with more than 10% of nucleotides showing Phred quality scores less than 30 were filtered out to generate cleaned reads.

To construct reference‐based assembly for the parent Xuzhou 68‐4, its cleaned reads were first aligned to the genomic sequences of *A. duranensis* and *A. ipaensis* (Bertioli *et al*., 2016) using the BWA software (Li and Durbin, 2009). The Coval software (Kosugi *et al*., 2013) was used to refine the short‐read sequence alignments. Then, SNPs were identified with the Samtools software and refined with the Coval software. Finally, the Xuzhou assembly was developed for male parent Xuzhou 68‐4 by substituting the reference bases with alternative bases at the positions of confidence SNPs in the genomic sequences of *A. duranensis* and *A. ipaensis*. Similarly, a reference‐based assembly, the Yuanza assembly, was constructed for the other parent Yuanza 9102.

To discover the genomic regions for QTLs controlling SP, the reads from high and low bulks were first aligned to the Xuzhou assembly, and SNPs were called for both the bulks. SNP‐index for each SNP position was calculated for both the bulks using the formula: $\text{SNP‐index (at a position)}=\frac{\text{Count of alternate base}}{\text{Count of reads aligned}}$ (Figure S3). ΔSNP‐index was then calculated by subtracting SNP‐index of high bulk from SNP‐index of low bulk for each SNP position except those with read depth <10 in both the bulks and SNP‐index <0.3 in either of the bulks. Sliding window analysis was conducted with 2 Mb interval and 50 kb increment for SNP‐index and ΔSNP‐index plots (Figure S3–S9). Only SNPs with ΔSNP‐index significantly higher than 0.5 or lower than −0.5 at the 0.01 confidence level were considered as the effective SNPs for SP. The effects of these SNPs were analysed with the SnpEff v3.0 software (Cingolani *et al*., 2012). On the other hand, the reads from high and low bulks were aligned to the Yuanza assembly to identify genomic regions for SP in the same way. The overlapped genomic regions identified with both parental assemblies were finally identified as the genomic regions controlling SP in the RIL population.

### KASP marker development and validation of identified candidate genomic regions

In order to validate the identified genomic regions for SP in the RIL population, 100 bp upstream and downstream sequences of genic SNPs were used for the development of KASP markers (He *et al*., 2014). For each SNP, two allele‐specific forward primers and one common reverse primer were designed and synthesized in LGC Genomic Ltd. Hoddesdon, UK. All KASP primers were listed in Table 4. These markers were used to genotype all individuals of the RIL population. The association between genotyping data and phenotyping data were calculated by single‐marker analysis (SMA) with the Windows QTL Cartographer software (Wang *et al*., 2012). The phenotyping data generated in the Yangluo2017 environment and the four environments used for making the pool as mentioned above were used for this analysis.

## Conflict of interest

The authors declare that there is no conflict of interest.

## Author contribution

H.L., M.K.P., R.K.V. and H.J. conceived, designed and supervised the experiments. Y.L., B.L. and H.J. developed the RIL population. H.L, J.G., B.W., Y.C., L.H., X.Z., Y.C., W.C. and N.L. conducted field trials and phenotyping. H.L., J.G. B.W. and Y.C. performed DNA extraction and genotyping. H.L., M.K.P., A.W.K., B.L., R.K.V. and H.J. performed the QTL‐seq analysis and interpreted the results. H.L. prepared the first draft and H.L., M.K.P., Y.L., B.L., R.K.V. and H.J. contributed to the final editing of manuscript. All authors read and approved the final manuscript.

## Supporting information


**Figure S1** Phenotypic differences in the representative RILs.

**Figure S2** Phenotypic distribution of shelling percentage in the RIL population across five environments.

**Figure S3** Alignment, SNP identification and calculation of SNP index for shelling percentage.

**Figure S4** SNP‐index plots for 20 pseudomolecules of low bulk with parent Xuzhou 68‐4 as reference.

**Figure S5** SNP‐index plots for 20 pseudomolecules of high bulk with parent Xuzhou 68‐4 as reference.

**Figure S6** The Δ(SNP‐index) plot obtained by subtraction of high pool SNP‐index from low pool SNP‐index using parent Xuzhou 68‐4 as reference.

**Figure S7** SNP‐index plots for 20 pseudomolecules of low bulk with parent Yuanza 9102 as reference.

**Figure S8** SNP‐index plots for 20 pseudomolecules of high bulk with parent Yuanza 9102 as reference.

**Figure S9** The Δ(SNP‐index) plot obtained by subtraction of high pool SNP‐index from low bulk SNP‐index using parent Yuanza 9102 as reference.

**Figure S10** Genotypes of the four KASP markers in the RIL population displayed in the SNPviewer software.

**Figure S11** Boxplot of shelling percentage for RILs with different combinations of the two identified QTLs in the present study.

**Figure S12** Boxplots of shelling percentages for diverse cultivars screened with KASP markers.

**Table S1** Details on the recombinant inbred lines (RILs) selected for construction of extreme bulks.

**Table S2** Details on whole genome re‐sequencing data generated on parental genotypes and bulks.

**Table S3** Pseudomolecule‐wise SNPs distribution between extreme bulks for shelling percentage.

**Table S4** Identification of SNPs for shelling percentage between extreme bulks using the Xuzhou assembly.

**Table S5** Identification of SNPs for shelling percentage between extreme bulks using the Yuanza assembly

**Table S6** Genotypes of the developed KASP markers in the RIL population.

**Table S7** Phenotyping data generated in the Yangluo2017 environment for the RIL population.

**Table S8** Phenotypic effect of the two major and stable QTLs for shelling percentage in the RIL population.

**Table S9** Putative genes and their functional annotations within the identified regions on chromosomes A09 and B02.

## References

[pbi13050-bib-0001] Agarwal, G. , Clevenger, J. , Pandey, M.K. , Wang, H. , Shasidhar, Y. , Chu, Y. , Fountain, J.C. *et al*. (2018) High‐density genetic map using whole‐genome resequencing for fine mapping and candidate gene discovery for disease resistance in peanut. Plant Biotechnol. J. 16, 1954–1967. 29637729 10.1111/pbi.12930 PMC6181220

[pbi13050-bib-0002] Bauer, E. , Falque, M. , Walter, H. , Bauland, C. , Camisan, C. , Campo, L. , Meyer, N. *et al*. (2013) Intraspecific variation of recombination rate in maize. Genome Biol. 14, R103. 24050704 10.1186/gb-2013-14-9-r103 PMC4053771

[pbi13050-bib-0003] Bertioli, D.J. , Cannon, S.B. , Froenicke, L. , Huang, G. , Farmer, A.D. , Cannon, E.K. , Liu, X. *et al*. (2016) The genome sequences of *Arachis duranensis* and *Arachis ipaensis,* the diploid ancestors of cultivated peanut. Nat. Genet. 48, 438–446. 26901068 10.1038/ng.3517

[pbi13050-bib-0004] Cai, Y. , Zhuang, X.H. , Gao, C.J. , Wang, X.F. and Jiang, L.W. (2014) The Arabidopsis endosomal sorting complex required for transport III regulates internal vesicle formation of the prevacuolar compartment and is required for plant development. Plant Physiol. 165, 1328–1343. 24812106 10.1104/pp.114.238378 PMC4081340

[pbi13050-bib-0005] Chen, X. , Li, H. , Pandey, M.K. , Yang, Q. , Wang, X. , Garg, V. , Li, H. *et al*. (2016) Draft genome of the peanut A‐genome progenitor (*Arachis duranensis*) provides insights into geocarpy, oil biosynthesis, and allergens. Proc. Natl Acad. Sci. USA 113, 6785–6790. 27247390 10.1073/pnas.1600899113 PMC4914189

[pbi13050-bib-0006] Cingolani, P. , Platts, A. , le Wang, L. , Coon, M. , Nguyen, T. , Wang, L. , Land, S.J. *et al*. (2012) A program for annotating and predicting the effects of single nucleotide polymorphisms, SnpEff: SNPs in the genome of *Drosophila melanogaster* strain *w* ^ *1118* ^ *; iso‐2; iso‐3* . Fly, 6, 80–92. 22728672 10.4161/fly.19695 PMC3679285

[pbi13050-bib-0007] Davis, J.P. and Dean, L.L. (2016) Chapter 11 ‐ Peanut composition, flavor and nutrition A2 ‐ Stalker, H. Thomas. In Peanuts ( Wilson, R.F. , ed), pp. 289–345. Urbana, IL: AOCS Press.

[pbi13050-bib-0008] Doyle, J. (1990) Isolation of plant DNA from fresh tissue. Focus, 12, 13–15.

[pbi13050-bib-0009] Dukic, M. , Berner, D. , Roesti, M. , Haag, C.R. and Ebert, D. (2016) A high‐density genetic map reveals variation in recombination rate across the genome of *Daphnia magna* . BMC Genet. 17, 137. 27737627 10.1186/s12863-016-0445-7 PMC5064971

[pbi13050-bib-0010] Faye, I. , Pandey, M.K. , Hamidou, F. , Rathore, A. , Ndoye, O. , Vadez, V. and Varshney, R.K. (2015) Identification of quantitative trait loci for yield and yield related traits in groundnut (*Arachis hypogaea* L.) under different water regimes in Niger and Senegal. Euphytica, 206, 631–647. 26594055 10.1007/s10681-015-1472-6 PMC4643859

[pbi13050-bib-0011] Han, Y.F. , Dou, K. , Ma, Z.Y. , Zhang, S.W. , Huang, H.W. , Li, L. , Cai, T. *et al*. (2014) SUVR2 is involved in transcriptional gene silencing by associating with SNF2‐related chromatin‐remodeling proteins in *Arabidopsis* . Cell Res. 24, 1445–1465. 25420628 10.1038/cr.2014.156 PMC4260354

[pbi13050-bib-0012] He, C. , Holme, J. and Anthony, J. (2014) SNP genotyping: the KASP assay. In Crop Breeding. Methods in Molecular Biology (Methods and Protocols) ( Fleury, D. and Whitford, R. , eds), pp. 75–86. New York: Humana Press. 10.1007/978-1-4939-0446-4_7 24816661

[pbi13050-bib-0013] Hua, Y. , Zhang, D. , Zhou, T. , He, M. , Ding, G. , Shi, L. , Xu, F. *et al*. (2016) Transcriptomics‐assisted quantitative trait locus fine mapping for the rapid identification of a nodulin 26‐like intrinsic protein gene regulating boron efficiency in allotetraploid rapeseed. Plant, Cell Environ. 39, 1601–1618. 26934080 10.1111/pce.12731

[pbi13050-bib-0014] Huang, L. , He, H.Y. , Chen, W.G. , Ren, X.P. , Chen, Y.N. , Zhou, X. , Xia, Y. *et al*. (2015) Quantitative trait locus analysis of agronomic and quality‐related traits in cultivated peanut (*Arachis hypogaea* L.). Theor. Appl. Genet. 128, 1103–1115. 25805315 10.1007/s00122-015-2493-1 PMC4434864

[pbi13050-bib-0015] Illa‐Berenguer, E. , Van Houten, J. , Huang, Z. and van der Knaap, E. (2015) Rapid and reliable identification of tomato fruit weight and locule number loci by QTL‐seq. Theor. Appl. Genet. 128, 1329–1342. 25893466 10.1007/s00122-015-2509-x

[pbi13050-bib-0016] Jiang, H.F. , Ren, X.P. , Chen, Y.N. , Huang, L. , Zhou, X.J. , Huang, J. , Froenicke, L. *et al*. (2013) Phenotypic evaluation of the Chinese mini‐mini core collection of peanut (*Arachis hypogaea* L.) and assessment for resistance to bacterial wilt disease caused by *Ralstonia solanacearum* . Plant Genet. Resour. 11, 77–83.

[pbi13050-bib-0017] Jiang, H.F. , Huang, L. , Ren, X.P. , Chen, Y.N. , Zhou, X.J. , Xia, Y. , Huang, J. *et al*. (2014) Diversity characterization and association analysis of agronomic traits in a Chinese peanut (*Arachis hypogaea* L.) mini‐core collection. J. Integr. Plant Biol. 56, 159–169. 24237710 10.1111/jipb.12132

[pbi13050-bib-0018] Kochert, G. , Stalker, H.T. , Gimenes, M. , Galgaro, L. , Lopes, C.R. and Moore, K. (1996) RFLP and cytogenetic evidence on the origin and evolution of allotetraploid domesticated peanut, *Arachis hypogaea* (Leguminosae). Am. J. Bot. 83, 1282–1291.

[pbi13050-bib-0019] Kosugi, S. , Natsume, S. , Yoshida, K. , MacLean, D. , Cano, L. , Kamoun, S. and Terauchi, R. (2013) Coval: improving alignment quality and variant calling accuracy for next‐generation sequencing data. PLoS ONE, 8, e75402. 24116042 10.1371/journal.pone.0075402 PMC3792961

[pbi13050-bib-0020] Li, H. and Durbin, R. (2009) Fast and accurate short read alignment with Burrows‐Wheeler transform. Bioinformatics, 25, 1754–1760. 19451168 10.1093/bioinformatics/btp324 PMC2705234

[pbi13050-bib-0021] Liu, W. , Duttke, S.H. , Hetzel, J. , Groth, M. , Feng, S. , Gallego‐Bartolome, J. , Zhong, Z. *et al*. (2018) RNA‐directed DNA methylation involves co‐transcriptional small‐RNA‐guided slicing of polymerase V transcripts in *Arabidopsis* . Nat. Plants 4, 181–188. 29379150 10.1038/s41477-017-0100-y PMC5832601

[pbi13050-bib-0022] Lu, H. , Lin, T. , Klein, J. , Wang, S. , Qi, J. , Zhou, Q. , Sun, J. *et al*. (2014) QTL‐seq identifies an early flowering QTL located near *Flowering Locus T* in cucumber. Theor. Appl. Genet. 127, 1491–1499. 24845123 10.1007/s00122-014-2313-z

[pbi13050-bib-0023] Luo, H. , Xu, Z. , Li, Z. , Li, X. , Lv, J. , Ren, X. , Huang, L. *et al*. (2017) Development of SSR markers and identification of major quantitative trait loci controlling shelling percentage in cultivated peanut (*Arachis hypogaea* L.). Theor. Appl. Genet. 130, 1635–1648. 28508097 10.1007/s00122-017-2915-3 PMC5511596

[pbi13050-bib-0024] Murtas, G. , Reeves, P.H. , Fu, Y.F. , Bancroft, I. , Dean, C. and Coupland, G. (2003) A nuclear protease required for flowering‐time regulation in *Arabidopsis* reduces the abundance of SMALL UBIQUITIN‐RELATED MODIFIER conjugates. Plant Cell, 15, 2308–2319. 14507998 10.1105/tpc.015487 PMC197297

[pbi13050-bib-0025] Pandey, M.K. , Roorkiwal, M. , Singh, V.K. , Ramalingam, A. , Kudapa, H. , Thudi, M. , Chitikineni, A. *et al*. (2016) Emerging genomic tools for legume breeding: current status and future prospects. Front. Plant Sci. 7, 455. 27199998 10.3389/fpls.2016.00455 PMC4852475

[pbi13050-bib-0026] Pandey, M.K. , Khan, A.W. , Singh, V.K. , Vishwakarma, M.K. , Shasidhar, Y. , Kumar, V. , Garg, V. *et al*. (2017) QTL‐seq approach identified genomic regions and diagnostic markers for rust and late leaf spot resistance in groundnut (*Arachis hypogaea* L.). Plant Biotechnol. J. 15, 927–941. 28028892 10.1111/pbi.12686 PMC5506652

[pbi13050-bib-0027] Salome, P.A. , Bomblies, K. , Fitz, J. , Laitinen, R.A. , Warthmann, N. , Yant, L. and Weigel, D. (2012) The recombination landscape in *Arabidopsis thaliana* F2 populations. Heredity (Edinb.) 108, 447–455. 22072068 10.1038/hdy.2011.95 PMC3313057

[pbi13050-bib-0028] Shirasawa, K. , Koilkonda, P. , Aoki, K. , Hirakawa, H. , Tabata, S. , Watanabe, M. , Hasegawa, M. *et al*. (2012) *In silico* polymorphism analysis for the development of simple sequence repeat and transposon markers and construction of linkage map in cultivated peanut. BMC Plant Biol. 12, 80. 22672714 10.1186/1471-2229-12-80 PMC3404960

[pbi13050-bib-0029] Singh, V.K. , Khan, A.W. , Jaganathan, D. , Thudi, M. , Roorkiwal, M. , Takagi, H. , Garg, V. *et al*. (2016a) QTL‐seq for rapid identification of candidate genes for 100‐seed weight and root/total plant dry weight ratio under rainfed conditions in chickpea. Plant Biotechnol. J. 14, 2110–2119. 27107184 10.1111/pbi.12567 PMC5095801

[pbi13050-bib-0030] Singh, V.K. , Khan, A.W. , Saxena, R.K. , Kumar, V. , Kale, S.M. , Sinha, P. , Chitikineni, A. *et al*. (2016b) Next‐generation sequencing for identification of candidate genes for *Fusarium* wilt and sterility mosaic disease in pigeonpea (*Cajanus cajan*). Plant Biotechnol. J. 14, 1183–1194. 26397045 10.1111/pbi.12470 PMC5054876

[pbi13050-bib-0031] Steele, K.A. , Quinton‐Tulloch, M.J. , Amgai, R.B. , Dhakal, R. , Khatiwada, S.P. , Vyas, D. , Heine, M. *et al*. (2018) Accelerating public sector rice breeding with high‐density KASP markers derived from whole genome sequencing of indica rice. Mol. Breeding 38, 38. 10.1007/s11032-018-0777-2 PMC5842261 29563850

[pbi13050-bib-0032] Takagi, H. , Abe, A. , Yoshida, K. , Kosugi, S. , Natsume, S. , Mitsuoka, C. , Uemura, A. *et al*. (2013) QTL‐seq: rapid mapping of quantitative trait loci in rice by whole genome resequencing of DNA from two bulked populations. Plant J. 74, 174–183. 23289725 10.1111/tpj.12105

[pbi13050-bib-0033] Uhlken, C. , Horvath, B. , Stadler, R. , Sauer, N. and Weingartner, M. (2014) MAIN‐LIKE1 is a crucial factor for correct cell division and differentiation in *Arabidopsis thaliana* . Plant J. 78, 107–120. 24635680 10.1111/tpj.12455

[pbi13050-bib-0034] Varshney, R.K. , Graner, A. and Sorrells, M.E. (2005) Genomics‐assisted breeding for crop improvement. Trends Plant Sci. 10, 621–630. 16290213 10.1016/j.tplants.2005.10.004

[pbi13050-bib-0035] Varshney, R.K. , Bertioli, D.J. , Moretzsohn, M.C. , Vadez, V. , Krishnamurthy, L. , Aruna, R. , Nigam, S.N. *et al*. (2009a) The first SSR‐based genetic linkage map for cultivated groundnut (*Arachis hypogaea* L.). Theor. Appl. Genet. 118, 729–739. 19048225 10.1007/s00122-008-0933-x

[pbi13050-bib-0036] Varshney, R.K. , Nayak, S.N. , May, G.D. and Jackson, S.A. (2009b) Next‐generation sequencing technologies and their implications for crop genetics and breeding. Trends Biotechnol. 27, 522–530. 19679362 10.1016/j.tibtech.2009.05.006

[pbi13050-bib-0037] Vishwakarma, M.K. , Nayak, S.N. , Guo, B. , Wan, L. , Liao, B. , Varshney, R.K. and Pandey, M.K. (2017) Classical and molecular approaches for mapping of genes and quantitative trait loci in peanut. In The Peanut Genome ( Varshney, R.K. , Pandey, M.K. and Puppala, N. , eds), pp. 93–116. Cham: Springer International Publishing.

[pbi13050-bib-0038] Wambugu, P. , Ndjiondjop, M.N. , Furtado, A. and Henry, R. (2018) Sequencing of bulks of segregants allows dissection of genetic control of amylose content in rice. Plant Biotechnol. J. 16, 100–110. 28499072 10.1111/pbi.12752 PMC5785344

[pbi13050-bib-0039] Wang, S. , Basten, C.J. and Zeng, Z.‐B. (2012) Windows QTL Cartographer 2.5. Department of Statistics, North Carolina State University, Raleigh, NC.

[pbi13050-bib-0040] Wang, Z. , Huai, D. , Zhang, Z. , Cheng, K. , Kang, Y. , Wan, L. , Yan, L. *et al*. (2018) Development of a high‐density genetic map based on specific length amplified fragment sequencing and its application in quantitative trait loci analysis for yield‐related traits in cultivated peanut. Front. Plant Sci. 9, 827. 29997635 10.3389/fpls.2018.00827 PMC6028809

[pbi13050-bib-0041] Wei, Q.Z. , Fu, W.Y. , Wang, Y.Z. , Qin, X.D. , Wang, J. , Li, J. , Lou, Q.F. *et al*. (2016) Rapid identification of fruit length loci in cucumber (*Cucumis sativus* L.) using next‐generation sequencing (NGS)‐based QTL analysis. Sci. Rep. 6, 27496. 27271557 10.1038/srep27496 PMC4895147

[pbi13050-bib-0042] Yol, E. , Furat, S. , Upadhyaya, H.D. and Uzun, B. (2018) Characterization of groundnut (*Arachis hypogaea* L.) collection using quantitative and qualitative traits in the Mediterranean Basin. J. Integr. Agric. 17, 63–75.

[pbi13050-bib-0043] Zhao, X.Y. , Chen, J. and Du, F.L. (2012) Potential use of peanut by‐products in food processing: a review. J. Food Sci. Technol. 49, 521–529. 24082262 10.1007/s13197-011-0449-2 PMC3550843

[pbi13050-bib-0044] Zhong, C. , Sun, S.L. , Li, Y.P. , Duan, C.X. and Zhu, Z.D. (2018) Next‐generation sequencing to identify candidate genes and develop diagnostic markers for a novel Phytophthora resistance gene, *RpsHC18*, in soybean. Theor. Appl. Genet. 131, 525–538. 29138903 10.1007/s00122-017-3016-z

[pbi13050-bib-0045] Zhou, X.J. , Xia, Y.L. , Ren, X.P. , Chen, Y.L. , Huang, L. , Huang, S. , Liao, B. *et al*. (2014) Construction of a SNP‐based genetic linkage map in cultivated peanut based on large scale marker development using next‐generation double‐digest restriction‐site‐associated DNA sequencing (ddRADseq). BMC Genom. 15, 14. 10.1186/1471-2164-15-351 PMC4035077 24885639

