# Supplementary figures and images for: Discovery of genomic regions and candidate genes controlling shelling percentage using QTL‐seq approach in cultivated peanut (Arachis hypogaea L.)
Source: Plant Biotechnol J. 2019 Jan 30;17(7):1248–60. doi: 10.1111/pbi.13050 (PMC6576108; doi:10.1111/pbi.13050)

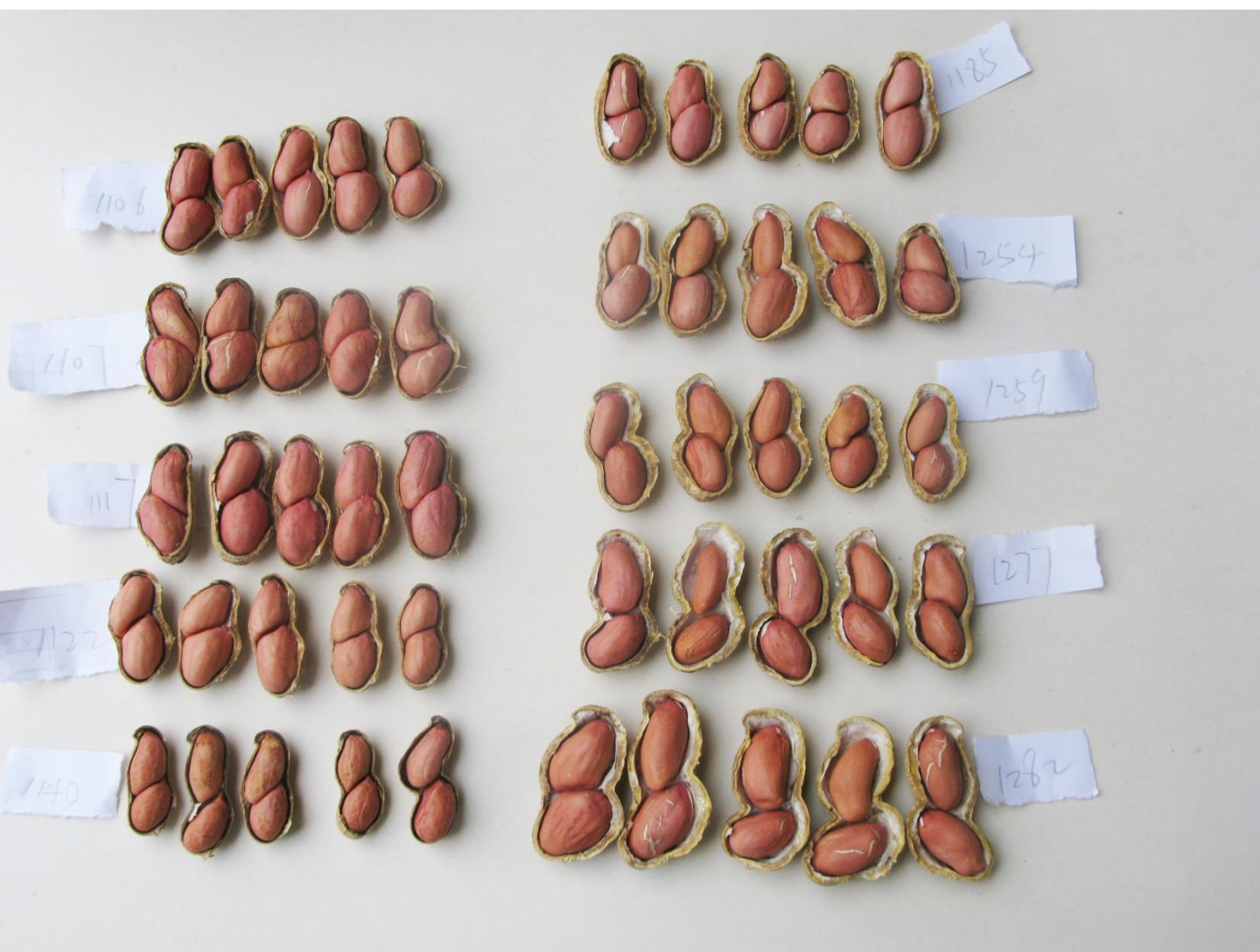

**Figure S1. Phenotypic differences in the representative RILs**

Supplement: Supplementary file 1 — Figure S1 Phenotypic differences in the representative RILs. [file PBI-17-1248-s019.pdf]
