# Supplementary material for: Discovery of genomic regions and candidate genes controlling shelling percentage using QTL‐seq approach in cultivated peanut (Arachis hypogaea L.)
Source: Plant Biotechnol J. 2019 Jan 30;17(7):1248–60. doi: 10.1111/pbi.13050 (PMC6576108; doi:10.1111/pbi.13050)
Supplement: Supplementary file 2 — Figure S2 Phenotypic distribution of shelling percentage in the RIL population across five environments. [file PBI-17-1248-s018.pdf]

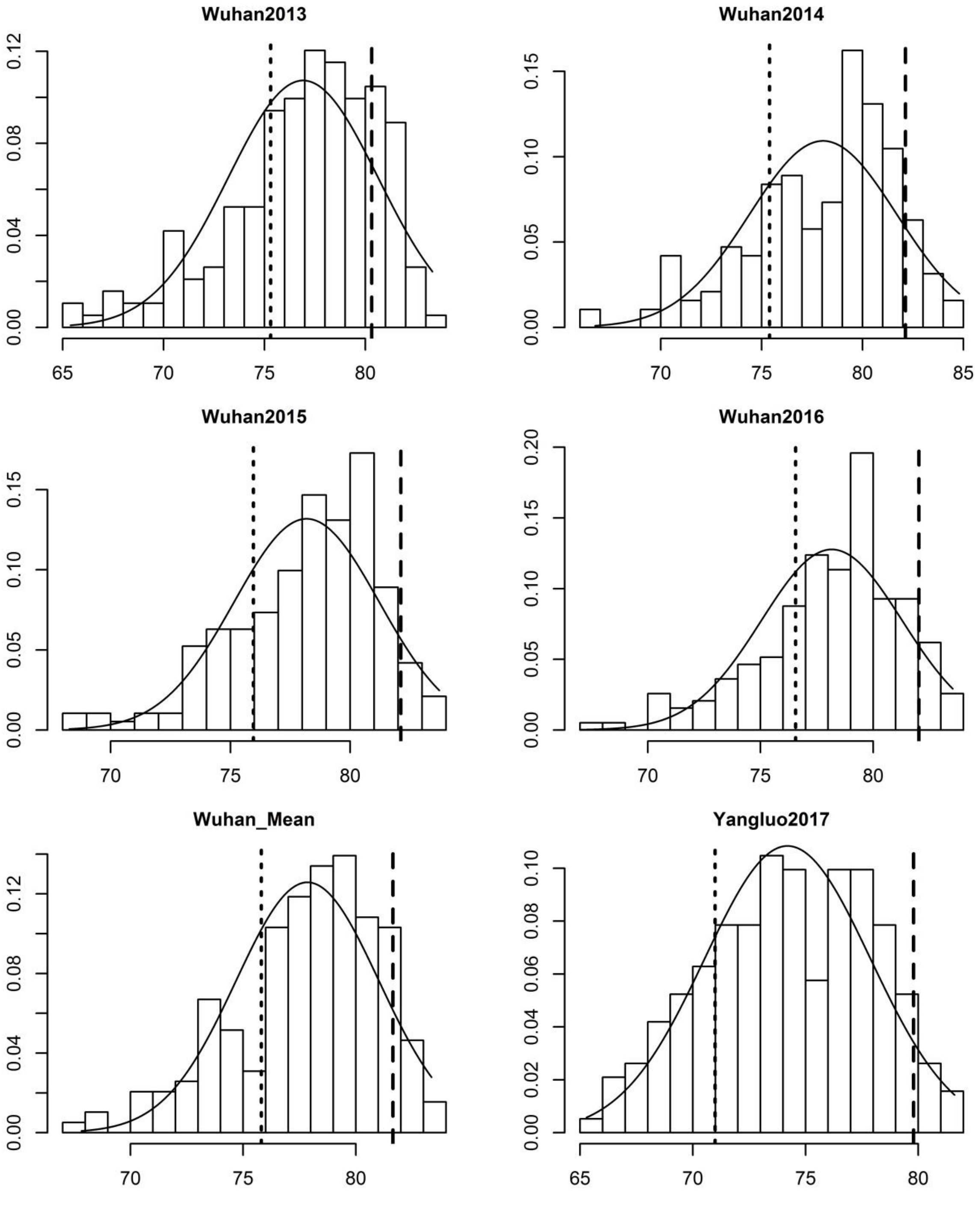

Figure S2 Phenotypic distribution of shelling percentage in the RIL population across five environments. The y-axis represented density, while the x-axis represented values of shelling percentage. The normal distribution curve in each graph represented the expected density. The mean value were calculated based on four environments, i.e., Wuhan2013, Wuhan2014, Wuhan2015 and Wuhan 2016, to construct the extreme bulks, and were designated as Wuhan_Mean. The data of the Yangluo2017 environment was generated after the bulk construction.
