# Supplementary material for: Discovery of genomic regions and candidate genes controlling shelling percentage using QTL‐seq approach in cultivated peanut (Arachis hypogaea L.)
Source: Plant Biotechnol J. 2019 Jan 30;17(7):1248–60. doi: 10.1111/pbi.13050 (PMC6576108; doi:10.1111/pbi.13050)
Supplement: Supplementary file 5 — Figure S5 SNP‐index plots for 20 pseudomolecules of high bulk with parent Xuzhou 68‐4 as reference. [file PBI-17-1248-s021.pdf]

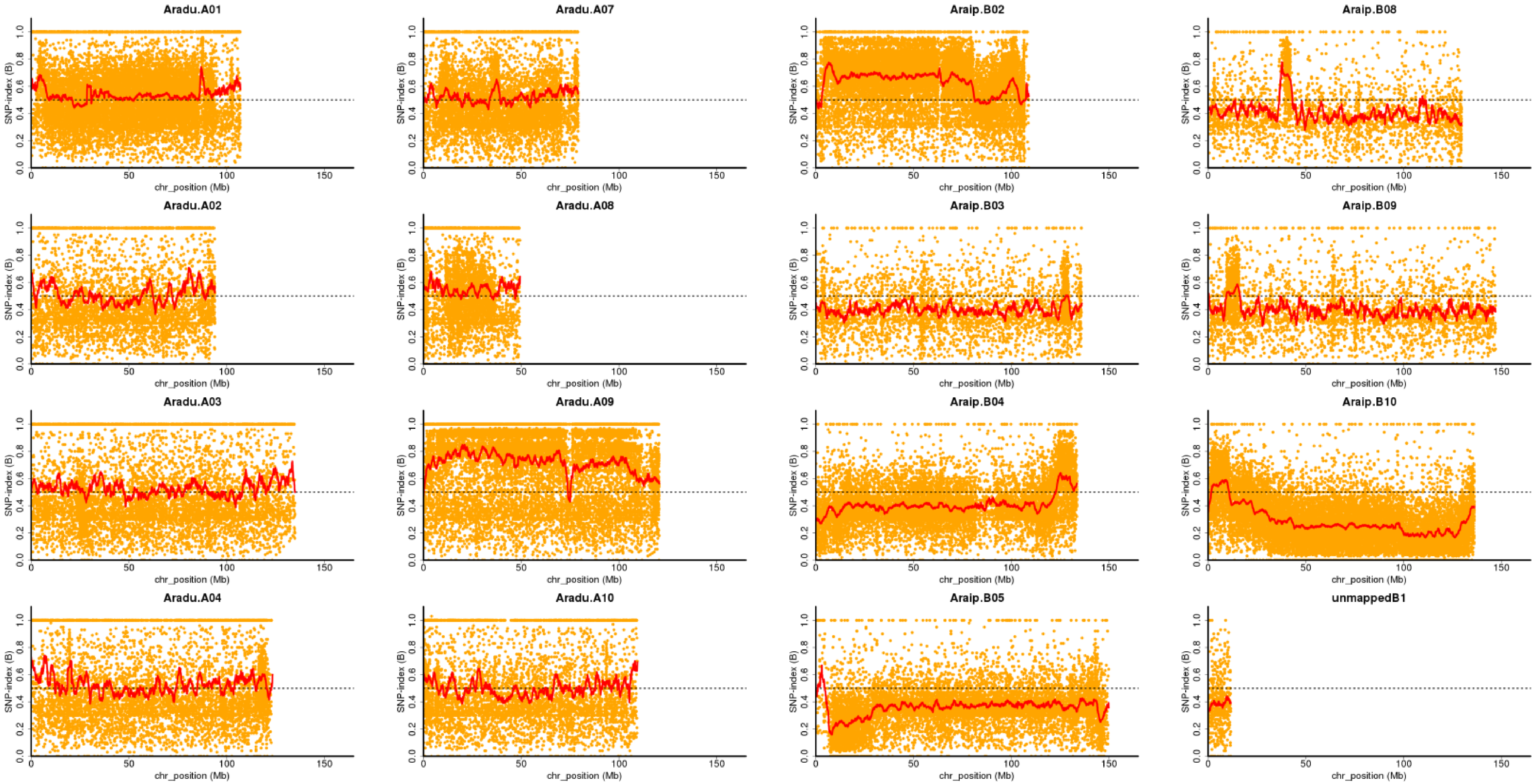

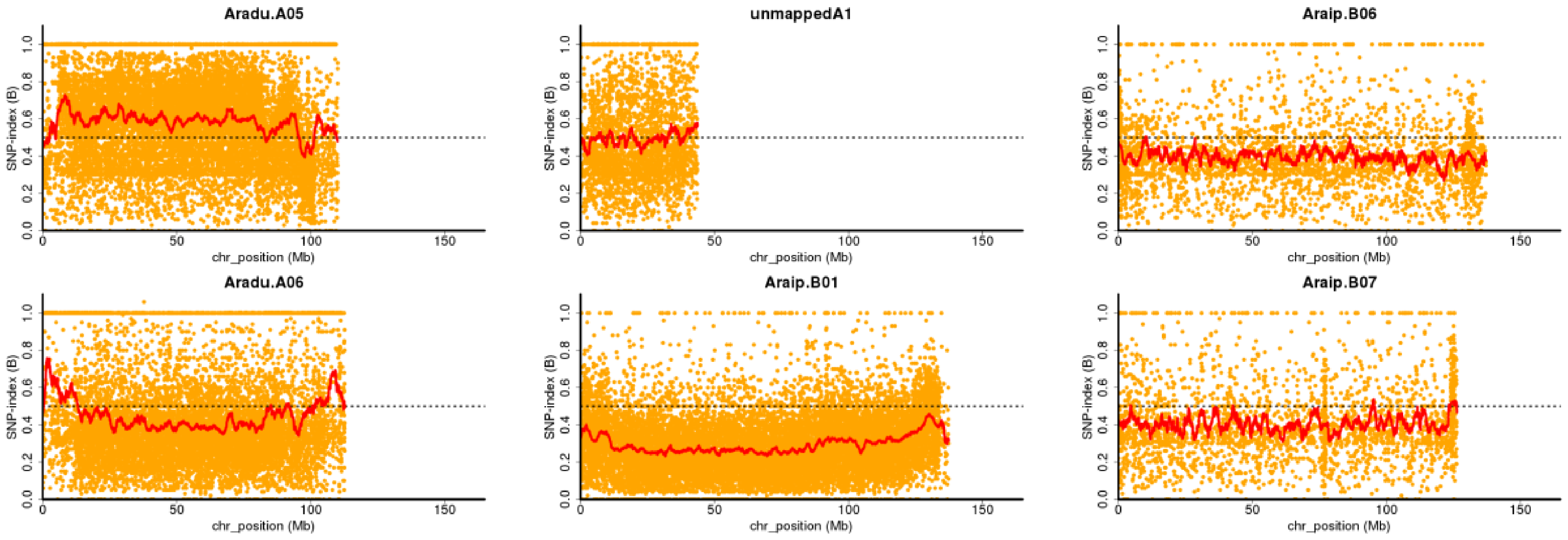

Figure S5. SNP-index plots for 20 pseudomolecules of high bulk with parent Xuzhou 68-4 as reference. Red lines indicate the sliding window average of 2 Mb interval with 50 kb increment for SNP-index. UnmappedA1 and UnmappedA1 represent the unmapped scaffolds for A and B subgenome, respectively.
