# Supplementary material for: Discovery of genomic regions and candidate genes controlling shelling percentage using QTL‐seq approach in cultivated peanut (Arachis hypogaea L.)
Source: Plant Biotechnol J. 2019 Jan 30;17(7):1248–60. doi: 10.1111/pbi.13050 (PMC6576108; doi:10.1111/pbi.13050)
Supplement: Supplementary file 6 — Figure S6 The Δ(SNP‐index) plot obtained by subtraction of high pool SNP‐index from low pool SNP‐index using parent Xuzhou 68‐4 as reference. [file PBI-17-1248-s002.pdf]

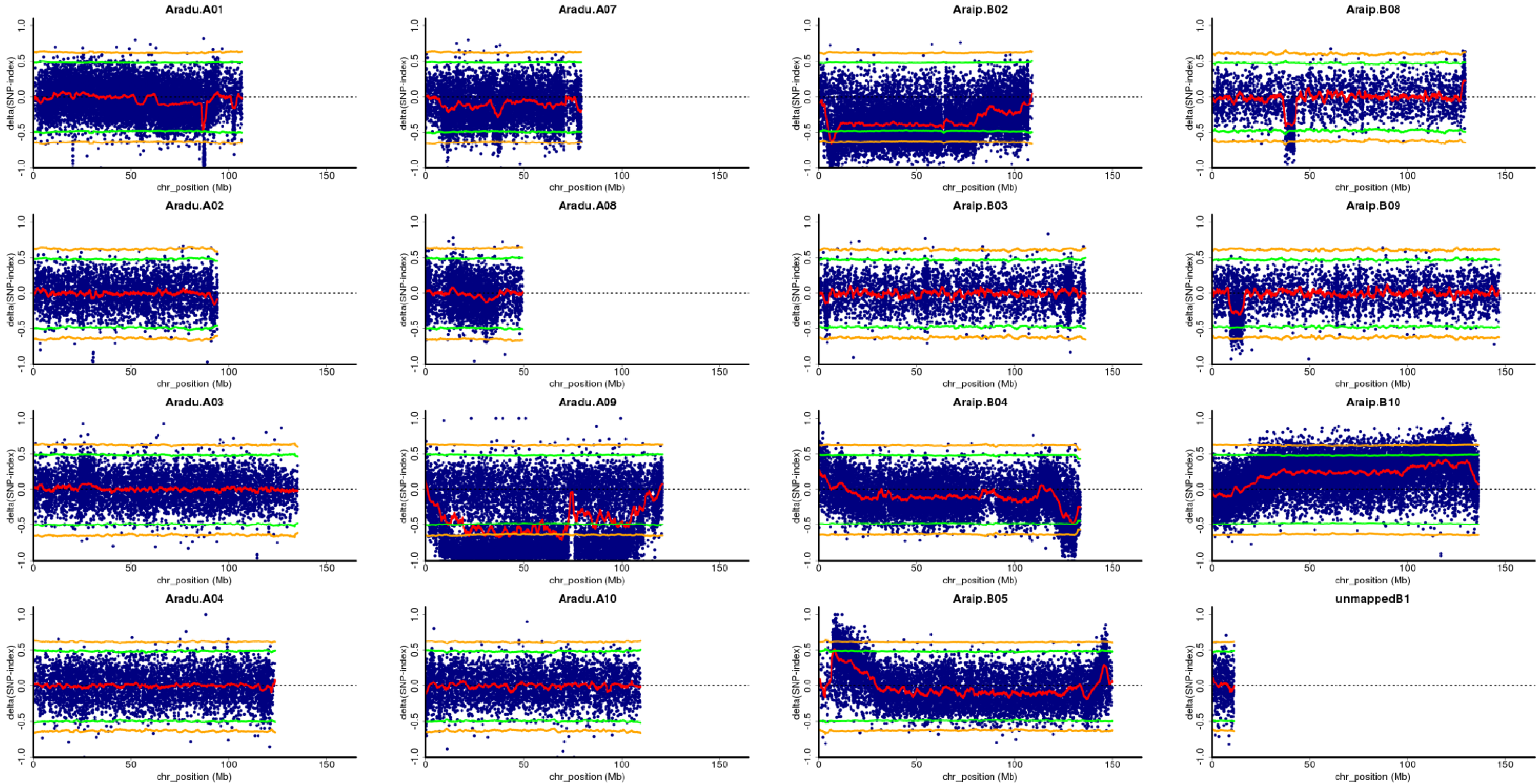

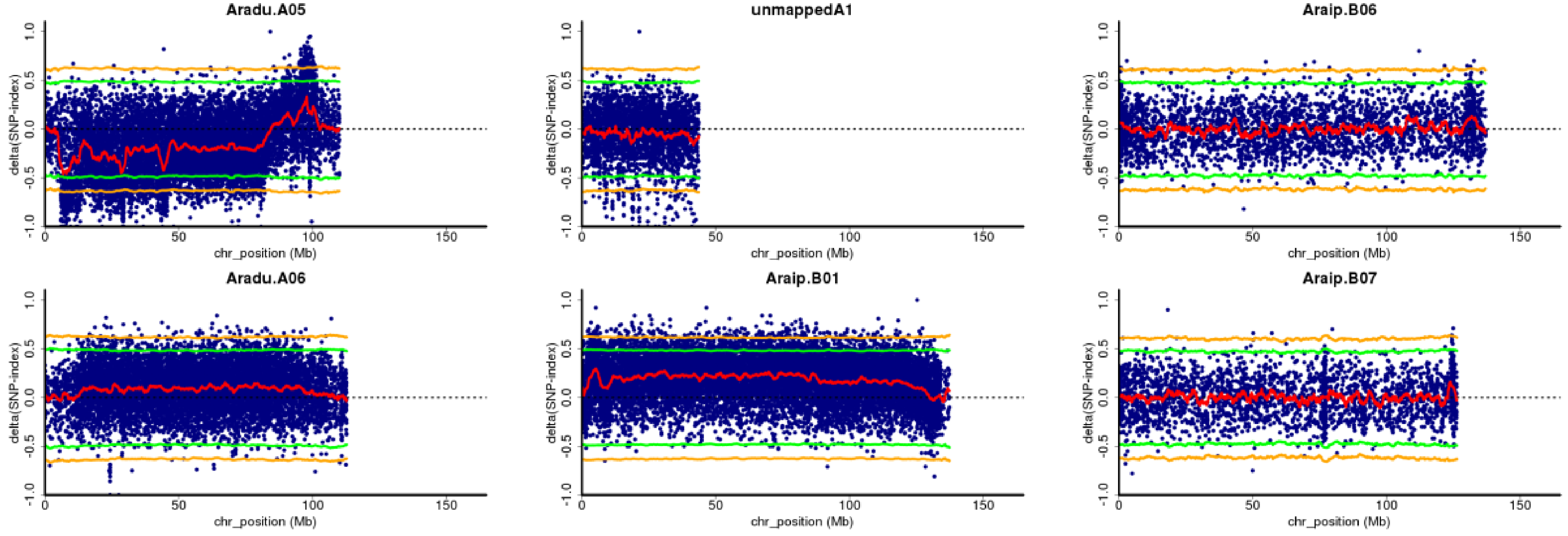

Figure S6. The Δ(SNP-index) plot obtained by subtraction of high pool SNP-index from low pool SNP-index using parent Xuzhou 68-4 as reference. Statistical confidence intervals under the null hypothesis of no QTL are shown (green: $P < 0.05$; orange: $P < 0.01$). UnmappedA1 and Unmapped B1 represent the unmapped scaffolds for A and B subgenome, respectively.
