# Supplementary material for: Discovery of genomic regions and candidate genes controlling shelling percentage using QTL‐seq approach in cultivated peanut (Arachis hypogaea L.)
Source: Plant Biotechnol J. 2019 Jan 30;17(7):1248–60. doi: 10.1111/pbi.13050 (PMC6576108; doi:10.1111/pbi.13050)
Supplement: Supplementary file 8 — Figure S8 SNP‐index plots for 20 pseudomolecules of high bulk with parent Yuanza 9102 as reference. [file PBI-17-1248-s004.pdf]

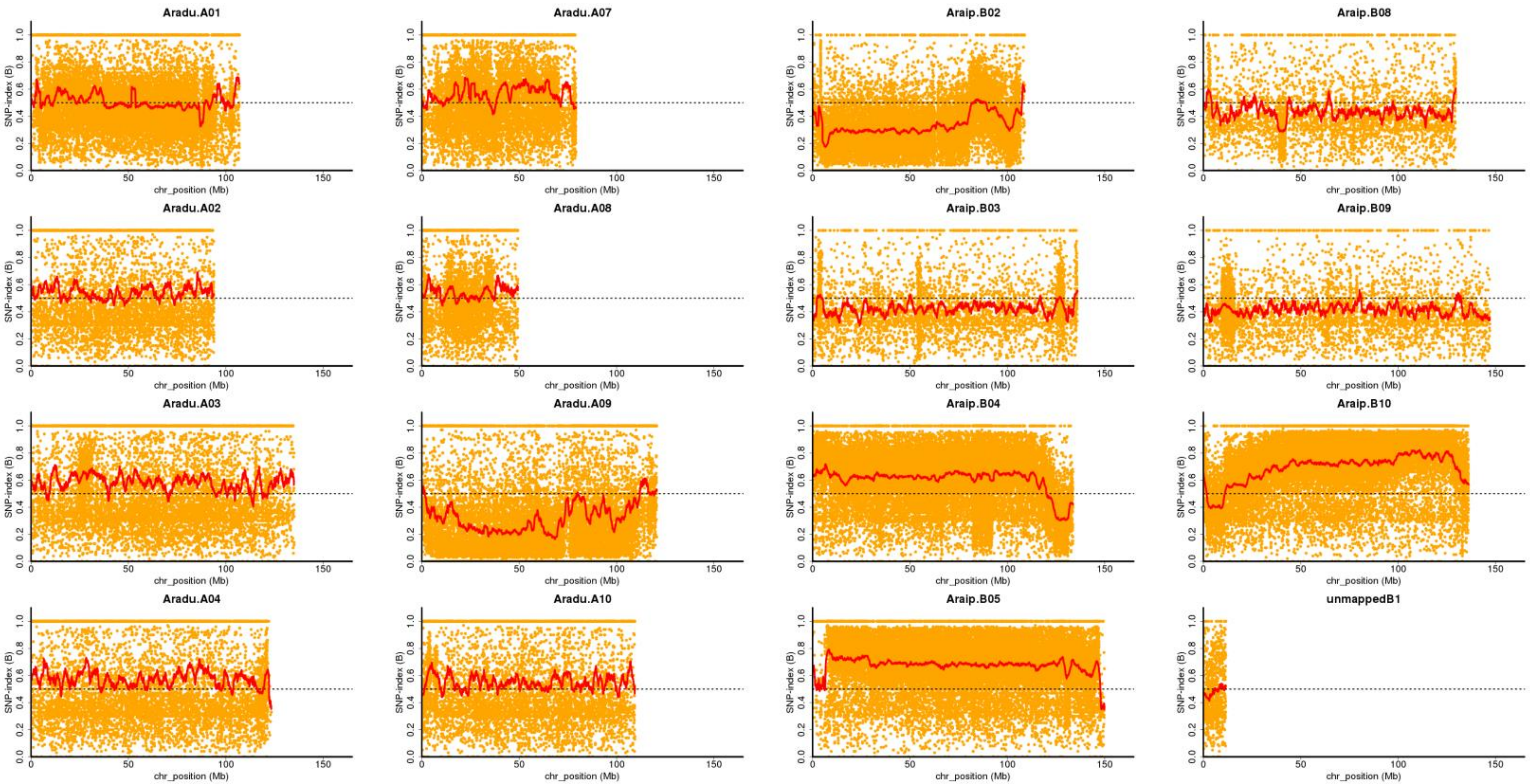

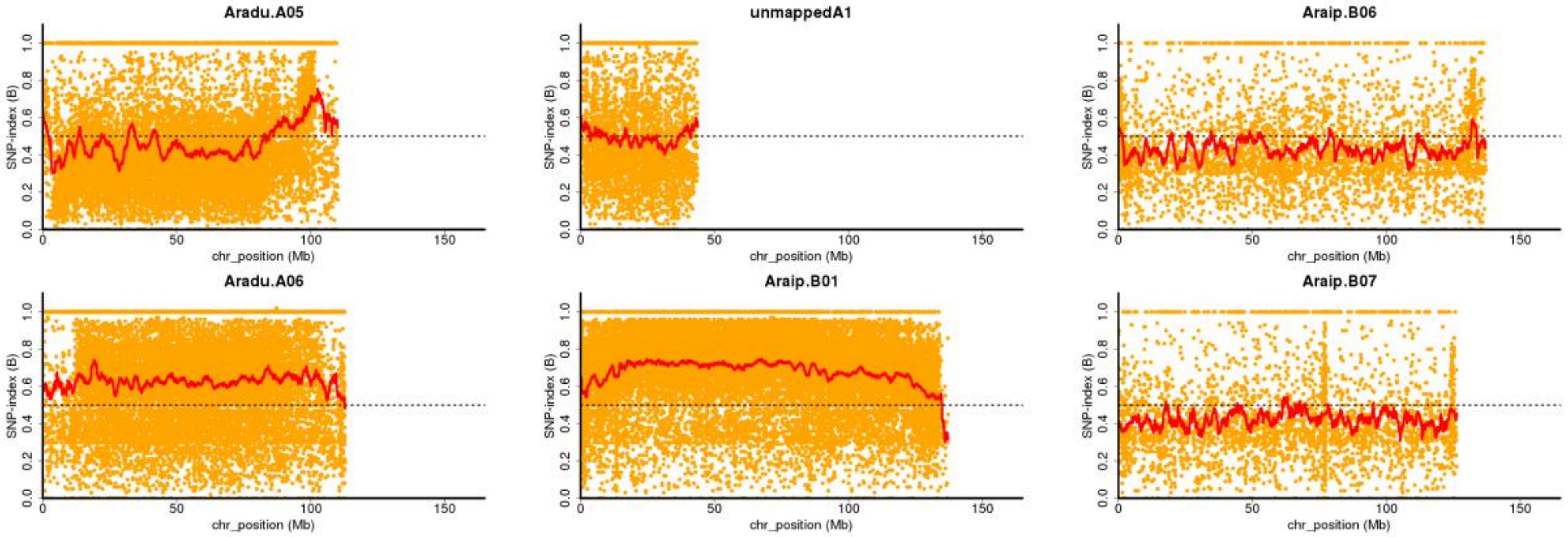

Figure S8. SNP-index plots for 20 pseudomolecules of high bulk with parent Yuanza 9102 as reference. Red lines indicate the sliding window average of 2Mb interval with 50 kb increment for SNP-index. UnmappedA1 and UnmappedB1 represent the unmapped scaffolds for A and B subgenome, respectively.
