# Supplementary material for: Discovery of genomic regions and candidate genes controlling shelling percentage using QTL‐seq approach in cultivated peanut (Arachis hypogaea L.)
Source: Plant Biotechnol J. 2019 Jan 30;17(7):1248–60. doi: 10.1111/pbi.13050 (PMC6576108; doi:10.1111/pbi.13050)
Supplement: Supplementary file 10 — Figure S10 Genotypes of the four KASP markers in the RIL population displayed in the SNPviewer software. [file PBI-17-1248-s006.pdf]

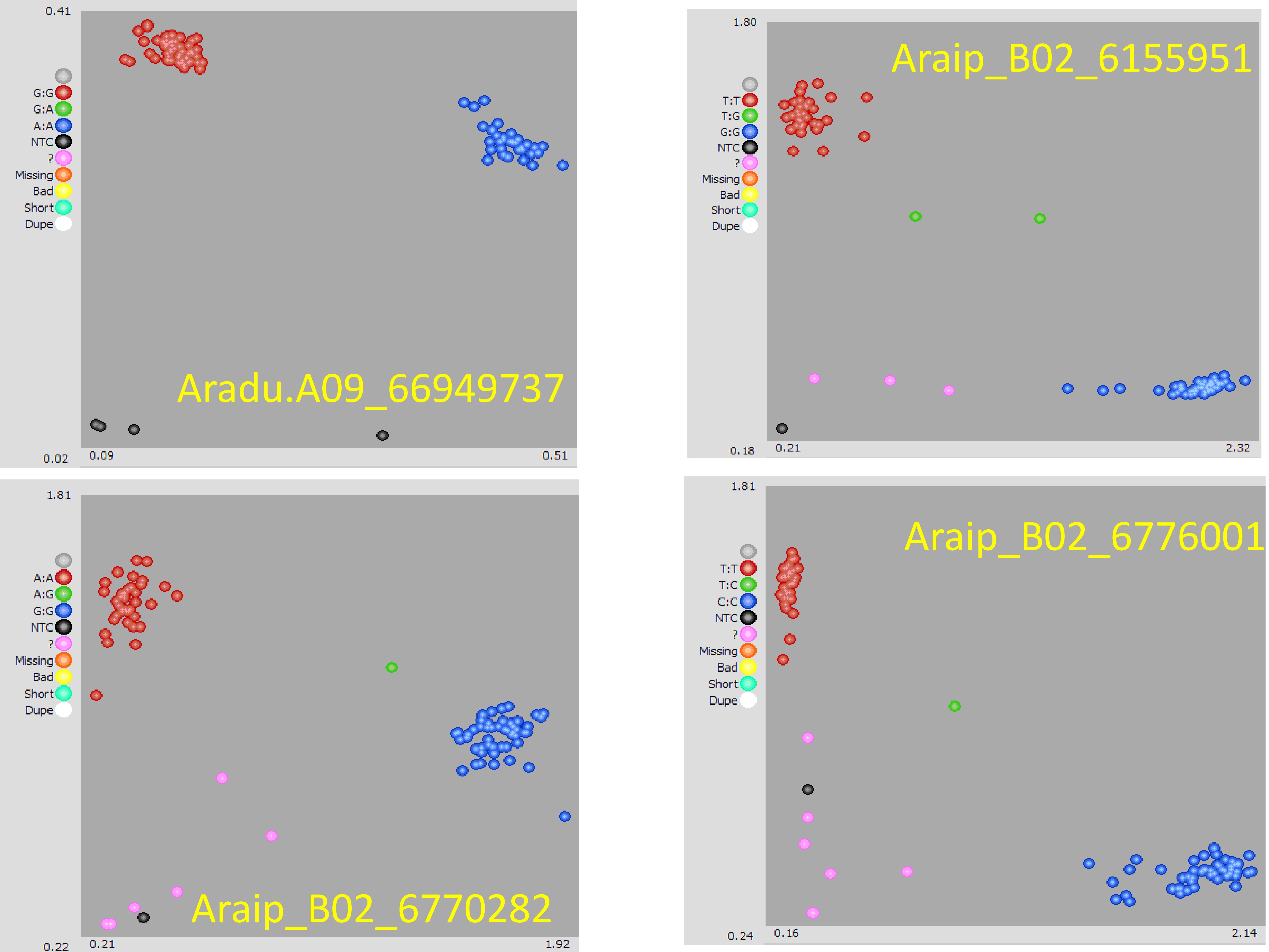

Figure S10. Genotypes of the four KASP markers in the RIL pupulation displayed in the SNPviewer software
