# Supplementary material for: Discovery of genomic regions and candidate genes controlling shelling percentage using QTL‐seq approach in cultivated peanut (Arachis hypogaea L.)
Source: Plant Biotechnol J. 2019 Jan 30;17(7):1248–60. doi: 10.1111/pbi.13050 (PMC6576108; doi:10.1111/pbi.13050)
Supplement: Supplementary file 11 — Figure S11 Boxplot of shelling percentage for RILs with different combinations of the two identified QTLs in the present study. [file PBI-17-1248-s007.pdf]

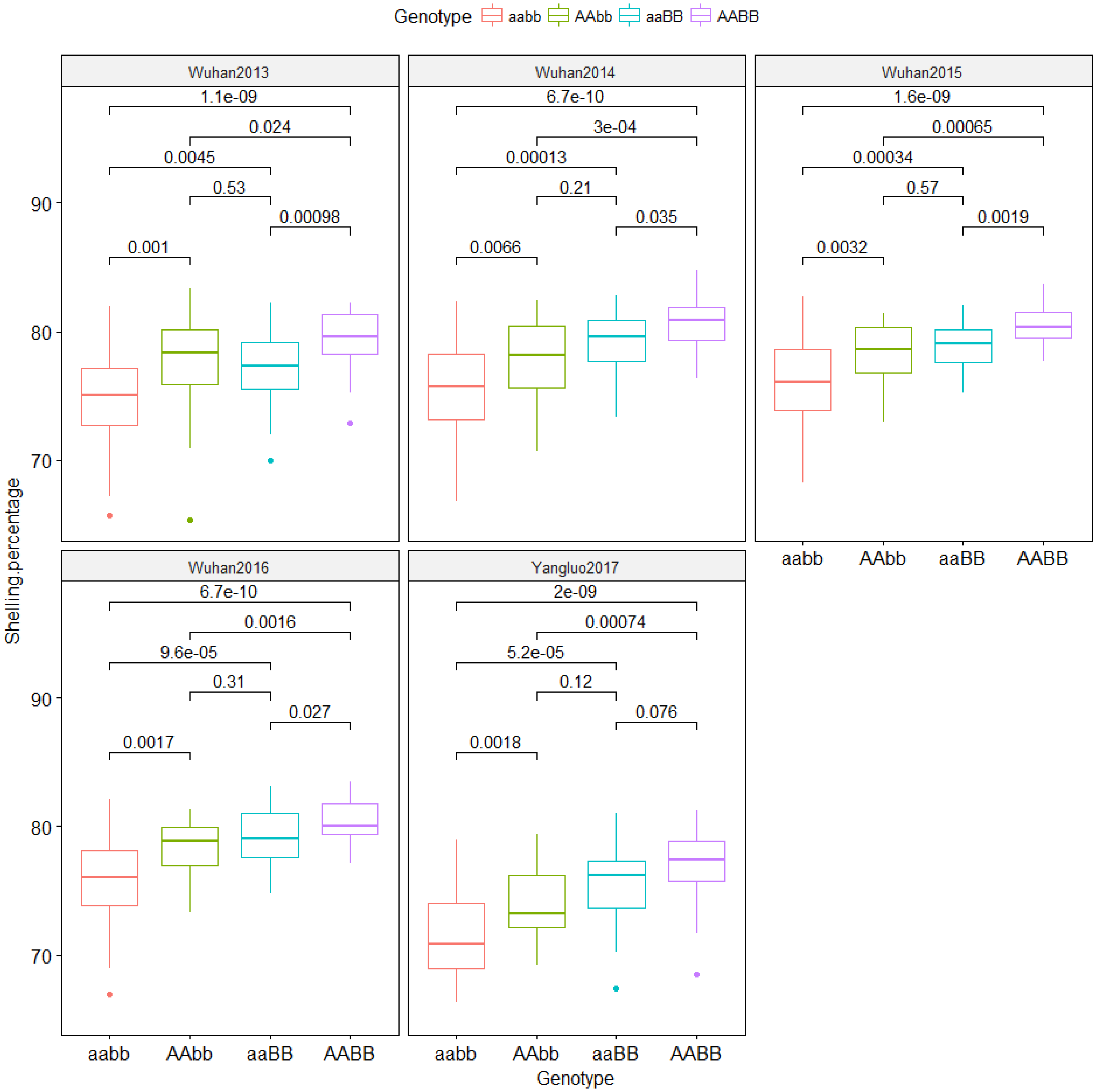

Figure S11 Boxplot of shelling percentage for RILs with different combinations of the two identified QTLs in the present study. The genotypes of the two QTLs in the RIL population were identified with the four KASP markers and classfied into four genotypic combinations. AA: genotype of KASP marker Aradu_A09_66949737 from Yuanza 9102, aa: genotype of KASP marker Aradu_A09_66949737 from Xuzhou 68-4, BB: genotype of KASP markers Araip_B02_6155951, Araip_B02_6770282 and Araip_B02_6776001 from Yuanza 9102, bb: genotype of KASP markers Araip_B02_6155951, Araip_B02_6770282 and Araip_B02_6776001 from Xuzhou 68-4. Boxplot were generated with the ggpubr package in R software. In each box, center line shows the median; box limits indicate the 25th and 75th percentiles; whiskers extend 1.5 times the interquartile range from the 25th and 75th percentiles. The p-values of mean comparisons between each pair of the four genotypes were calculated using wilcox.test and showed above boxes.
