# Supplementary material for: Discovery of genomic regions and candidate genes controlling shelling percentage using QTL‐seq approach in cultivated peanut (Arachis hypogaea L.)
Source: Plant Biotechnol J. 2019 Jan 30;17(7):1248–60. doi: 10.1111/pbi.13050 (PMC6576108; doi:10.1111/pbi.13050)
Supplement: Supplementary file 12 — Figure S12 Boxplots of shelling percentages for diverse cultivars screened with KASP markers. [file PBI-17-1248-s008.pdf]

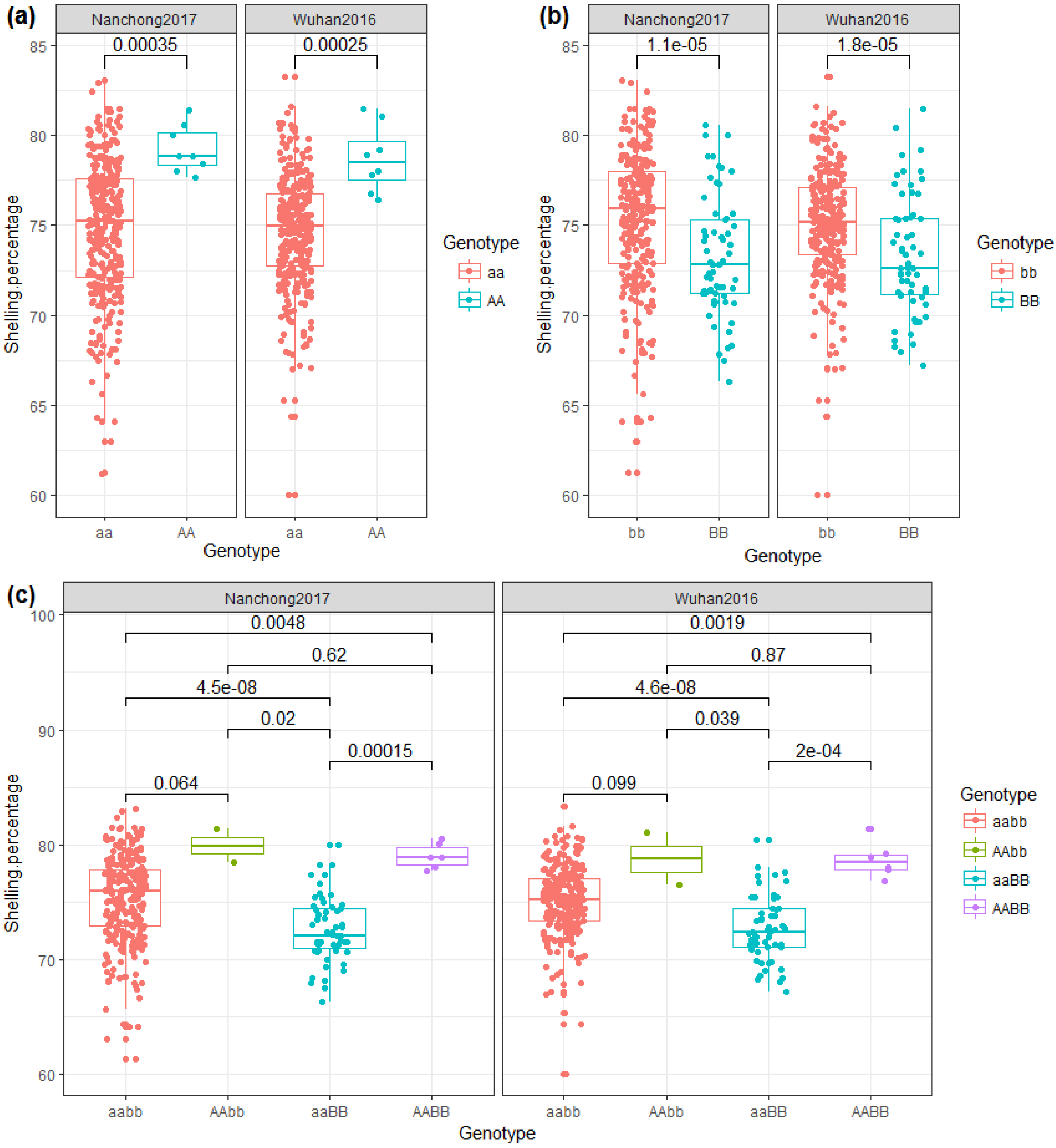

Figure S12 Boxplots of shelling percentages for diverse cultivars screened with KASP markers. (a) The difference of shelling percentages of cultivars with different genotypes for the QTL on A09. AA: genotype of KASP marker Aradu_A09_66949737 from Yuanza 9102; aa: genotype of KASP marker Aradu_A09_66949737 from Xuzhou 68-4. (b) The difference of shelling percentages of cultivars with different genotypes for the QTL on B02. BB: genotype of KASP markers Araip_B02_6155951, Araip_B02_6770282 and Araip_B02_6776001 from Yuanza 9102; bb: genotype of KASP markers Araip_B02_6155951, Araip_B02_6770282 and Araip_B02_6776001 from Xuzhou 68-4. (c) The difference of shelling percentages of cultivars with different combinations of the two QTLs. Boxplots were generated with the ggpubr package in R software. In each box, center line shows the median; box limits indicate the 25th and 75th percentiles; whiskers extend 1.5 times the interquartile range from the 25th and 75th percentiles. The p-values of mean comparisons between each pair of genotypes were calculated using wilcox.test and showed above boxes.
