# Supplementary material for: Discovery of genomic regions and candidate genes controlling shelling percentage using QTL‐seq approach in cultivated peanut (Arachis hypogaea L.)
Source: Plant Biotechnol J. 2019 Jan 30;17(7):1248–60. doi: 10.1111/pbi.13050 (PMC6576108; doi:10.1111/pbi.13050)
Supplement: Supplementary file 13 — Table S1 Details on the recombinant inbred lines (RILs) selected for construction of extreme bulks. [file PBI-17-1248-s009.pdf]

Table S1 Details on the recombinant inbred lines (RILs) selected for construction of extreme bulks.

	Lowest bulk		Highest bulk	
**S. No.**	**RIL name**	**Shelling**	**RIL name**	**Shelling percent**
1	QT644	67.83	QT506	81.71
2	QT643	68.89	QT678	81.87
3	QT602	68.95	QT514	81.90
4	QT523	70.30	QT512	82.02
5	QT648	70.70	QT611	82.06
6	QT631	70.78	QT511	82.12
7	QT544	70.81	QT543	82.13
8	QT500	71.03	QT588	82.36
9	QT666	71.55	QT668	82.43
10	QT535	71.77	QT495	82.59
11	QT671	71.93	QT641	82.75
12	QT574	72.14	QT571	82.92
13	QT502	72.27	QT529	83.04
14	QT504	72.62	QT640	83.04
15	QT677	72.87	QT619	83.36
Mean	Bulk mean	70.96	Bulk mean	82.42
Yuanza 9102	High shelling percentage parent	81.65	High shelling percentage parent	81.65
Xuzhou 68-4	Low shelling percentage parent	75.81	Low shelling percentage parent	75.81

Note: Cross: Yuanza 9102 x Xuzhou 68-4; RIL generation: F9.
