# Supplementary material for: Discovery of genomic regions and candidate genes controlling shelling percentage using QTL‐seq approach in cultivated peanut (Arachis hypogaea L.)
Source: Plant Biotechnol J. 2019 Jan 30;17(7):1248–60. doi: 10.1111/pbi.13050 (PMC6576108; doi:10.1111/pbi.13050)
Supplement: Supplementary file 14 — Table S2 Details on whole genome re‐sequencing data generated on parental genotypes and bulks. [file PBI-17-1248-s010.pdf]

Table S2 Details on whole genome re-sequencing data generated on parental genotypes and bulks.

Sample ID	Genotype / bulks	Total reads generated	Total data (Bases)	Clean reads	High quality reads	Equalized Reads	Mapped reads after filtering	Genome coverage at >= 1x (%)	Genome coverage at >=5 x (%)	Genome coverage at >=10x (%)	Genome coverage at >= 15x (%)	Genome coverage at >= 20x (%)	Bases not covered (%)	Avg depth (X)
Xuzhou 68-4	Low shelling percentage parent	544,700,200	82,249,730,200	467,742,004	387,081,532		236,502,616	92.61%	86.51%	72.18%	45.68%	21.63%	7.39%	16.18
LB	Low shelling percentage bulk	717,723,902	108,376,309,202	614,246,800	503,558,512	441,628,028	306,345,180	93.12%	88.47%	81.71%	67.07%	44.30%	6.88%	20.96
HB	High shelling percentage bulk	790,320,814	119,338,442,914	682,685,602	566,026,261	441,628,028	312,360,116	93.18%	88.60%	82.19%	68.67%	46.72%	6.82%	21.37
Yuanza 9102	High shelling percentage parent	456,893,014	68,990,845,114	399,725,876	327,886,582		205,242,874	91.85%	84.41%	64.20%	33.08%	12.78%	8.15%	14.04
LB	Low shelling percentage bulk	717,723,902	108,376,309,202	614,246,800	503,558,512	441,628,028	306,267,367	93.06%	88.36%	81.59%	66.97%	44.23%	6.94%	20.96
HB	High shelling percentage bulk	790,320,814	119,338,442,914	682,685,602	566,026,261	441,628,028	312,304,015	93.12%	88.49%	82.08%	68.58%	46.65%	6.88%	21.37
