# Supplementary material for: Discovery of genomic regions and candidate genes controlling shelling percentage using QTL‐seq approach in cultivated peanut (Arachis hypogaea L.)
Source: Plant Biotechnol J. 2019 Jan 30;17(7):1248–60. doi: 10.1111/pbi.13050 (PMC6576108; doi:10.1111/pbi.13050)
Supplement: Supplementary file 15 — Table S3 Pseudomolecule‐wise SNPs distribution between extreme bulks for shelling percentage. [file PBI-17-1248-s011.pdf]

Table S3 Pseudomolecule-wise SNPs distribution between extreme bulks for shelling percentage.

Linkage group	Total number of SNPs	Number of homozygous SNPs	Percentage of homozygous SNPs (% of total)	Depth range (X coverage) in low bulk	Depth range (X coverage) in high bulk	Start and end position of SNPs
Xuzhou 68-4 as reference						
Aradu.A01	13,057	3,084	23.62	10-98	10-99	2893-107000700
Aradu.A02	4,603	2,667	57.94	10-105	10-92	46918-93864408
Aradu.A03	7,630	4,351	57.02	10-116	10-104	1187-134986391
Aradu.A04	6,475	3,813	58.89	10-92	10-109	115730-123303739
Aradu.A05	12,850	4,697	36.55	10-91	10-116	6321-110027663
Aradu.A06	10,783	3,691	34.23	10-92	10-109	5820-112645067
Aradu.A07	7,205	3,169	43.98	10-103	10-124	13937-79126321
Aradu.A08	4,038	1,474	36.50	10-106	10-87	16788-49364020
Aradu.A09	14,406	10,672	74.08	10-89	10-99	67045-120587479
Aradu.A10	5,207	2,875	55.21	10-95	10-112	11398-109354527
Unmapped_Aradu	2,997	1,447	48.28	10-146	10-87	
Araip.B01	14,730	2,698	18.32	10-114	10-87	11058-137332285
Araip.B02	14,585	3,754	25.74	10-105	10-97	190-108956324
Araip.B03	3,693	1,204	32.60	10-100	10-95	47583-135928663
Araip.B04	11,365	2,993	26.34	10-92	10-89	81927-133612283
Araip.B05	9,548	2,719	28.48	10-88	10-93	53763-149832347
Araip.B06	3,148	1,206	38.31	10-109	10-88	21697-137080815
Araip.B07	2,723	1,052	38.63	10-106	10-101	51886-126197411
Araip.B08	3,069	1,194	38.91	10-96	10-99	81607-129598696
Araip.B09	3,748	1,354	36.13	10-89	10-109	558969-147049874
Araip.B10	16,315	3,696	22.65	10-92	10-95	87201-136128093
Unmapped_Araip	540	155	28.70	10-79	10-82	
Total	172,715	63,965	37.03			
Yuanza 9102 assembly as reference						
Aradu.A01	13,823	3,869	27.99	10-100	10-100	1918-107025846
Aradu.A02	5,547	3,240	58.41	10-113	10-93	28222-93864408
Aradu.A03	10,066	6,098	60.58	10-101	10-92	9727-135046652
Aradu.A04	8,376	5,085	60.71	10-98	10-98	31924-123441652
Aradu.A05	14,282	5,727	40.10	10-100	10-115	6283-110022998
Aradu.A06	14,776	5,375	36.38	10-112	10-88	8196-112696872
Aradu.A07	10,608	4,917	46.35	10-87	10-92	1089-79123284
Aradu.A08	4,198	1,590	37.88	10-82	10-78	31056-49341383
Aradu.A09	16,647	12,361	74.25	10-106	10-119	77558-120587481
Aradu.A10	6,751	3,823	56.63	10-107	10-99	11398-109443170
Unmapped_Aradu	3,836	1,821	47.47	10-123	10-115	
Araip.B01	21,632	4,265	19.72	10-101	10-97	37183-137224838
Araip.B02	15,688	4,259	27.15	10-128	10-111	190-108956493
Araip.B03	4,394	1,490	33.91	10-92	10-90	176024-135928721
Araip.B04	26,603	8,204	30.84	10-98	10-103	12673-133614414
Araip.B05	25,475	9,178	36.03	10-98	10-103	22399-149832347
Araip.B06	3,872	1,436	37.09	10-87	10-92	25025-137092770
Araip.B07	3,169	1,205	38.02	10-94	10-99	3815-126247990
Araip.B08	3,786	1,439	38.01	10-96	10-100	94459-129598696
Araip.B09	4,512	1,637	36.28	10-87	10-88	67237-147049874
Araip.B10	22,173	5,549	25.03	10-89	10-119	24136-136128093
Unmapped_Araip	864	221	25.58	10-99	10-93	
Total	241,078	92,789	38.49			
