# Supplementary material for: Discovery of genomic regions and candidate genes controlling shelling percentage using QTL‐seq approach in cultivated peanut (Arachis hypogaea L.)
Source: Plant Biotechnol J. 2019 Jan 30;17(7):1248–60. doi: 10.1111/pbi.13050 (PMC6576108; doi:10.1111/pbi.13050)
Supplement: Supplementary file 16 — Table S4 Identification of SNPs for shelling percentage between extreme bulks using the Xuzhou assembly. [file PBI-17-1248-s012.pdf]

Table S4 Identification of SNPs for shelling percentage between extreme bulks using the Xuzhou assembly.

Pseudomolecule	Physical position (bp)	Reference base of Xuzhou assembly	Consensus base of LB	Number of reads covering the	SNP-index of LB	Consensus base of HB	Number of reads covering the site	SNP-index of HB	delta SNP-index (LB SNP-index-HB	U95 (95% confidence interval upper side)	L95 (95% confidence interval lower side)	U99 (99% confidence interval upper side)	L99 (99% confidence interval lower side)	SNP substitution effect	SNP substitution impact	SNP substitution	Amino acid change	Gene	Function
Aradu.A09	66760246	C	C	20	0.10	T	26	0.80	-0.70	0.45	-0.45	0.60	-0.60	intergenic_region	MODIFIER	n.66760246C>T		Aradu.G83RZ-Aradu.A5UR9	
Aradu.A09	66761353	G	G	27	0.04	A	26	0.80	-0.76	0.42	-0.46	0.54	-0.58	intergenic_region	MODIFIER	n.66761353G>A		Aradu.G83RZ-Aradu.A5UR9	
Aradu.A09	66770791	A	A	25	0.08	T	18	1.00	-0.92	0.50	-0.50	0.61	-0.61	intergenic_region	MODIFIER	n.66770791A>T		Aradu.G83RZ-Aradu.A5UR9	
Aradu.A09	66804711	C	C	15	0.13	T	40	0.82	-0.69	0.53	-0.47	0.67	-0.67	intergenic_region	MODIFIER	n.66804711C>T		Aradu.G83RZ-Aradu.A5UR9	
Aradu.A09	66805161	A	A	15	0.07	T	10	0.90	-0.83	0.50	-0.60	0.70	-0.70	intergenic_region	MODIFIER	n.66805161T>A		Aradu.G83RZ-Aradu.A5UR9	
Aradu.A09	66812220	C	C	10	0.00	T	12	1.00	-1.00	0.50	-0.60	0.70	-0.70	intergenic_region	MODIFIER	n.66812220C>T		Aradu.G83RZ-Aradu.A5UR9	
Aradu.A09	66816902	A	A	24	0.00	G	25	0.68	-0.68	0.46	-0.46	0.54	-0.58	intergenic_region	MODIFIER	n.66816902A>G		Aradu.G83RZ-Aradu.A5UR9	
Aradu.A09	66825378	A	A	28	0.14	G	26	0.96	-0.82	0.42	-0.46	0.54	-0.58	intergenic_region	MODIFIER	n.66825378G>A		Aradu.G83RZ-Aradu.A5UR9	
Aradu.A09	66843473	C	C	28	0.00	T	18	0.66	-0.66	0.50	-0.50	0.61	-0.61	intergenic_region	MODIFIER	n.66843473C>T		Aradu.G83RZ-Aradu.A5UR9	
Aradu.A09	66857809	T	T	18	0.06	C	16	0.75	-0.69	0.50	-0.50	0.63	-0.63	intergenic_region	MODIFIER	n.66857809C>T		Aradu.G83RZ-Aradu.A5UR9	
Aradu.A09	66860937	C	C	18	0.00	A	25	0.80	-0.80	0.50	-0.50	0.61	-0.61	intergenic_region	MODIFIER	n.66860937C>A		Aradu.G83RZ-Aradu.A5UR9	
Aradu.A09	66892507	A	A	21	0.00	C	16	0.81	-0.81	0.50	-0.50	0.63	-0.63	intergenic_region	MODIFIER	n.66892507C>A		Aradu.G83RZ-Aradu.A5UR9	
Aradu.A09	66893279	A	A	15	0.00	G	23	0.91	-0.91	0.53	-0.47	0.67	-0.67	intergenic_region	MODIFIER	n.66893279G>A		Aradu.G83RZ-Aradu.A5UR9	
Aradu.A09	66897373	C	C	22	0.00	T	24	0.83	-0.83	0.45	-0.45	0.59	-0.59	intergenic_region	MODIFIER	n.66897373C>T		Aradu.G83RZ-Aradu.A5UR9	
Aradu.A09	66897379	T	T	23	0.00	A	24	0.79	-0.79	0.43	-0.43	0.57	-0.61	intergenic_region	MODIFIER	n.66897379T>A		Aradu.G83RZ-Aradu.A5UR9	
Aradu.A09	66920252	G	G	15	0.13	C	19	0.94	-0.81	0.53	-0.47	0.67	-0.67	intergenic_region	MODIFIER	n.66920252G>C		Aradu.G83RZ-Aradu.A5UR9	
Aradu.A09	66921802	C	C	25	0.04	T	19	0.94	-0.90	0.47	-0.47	0.63	-0.63	intergenic_region	MODIFIER	n.66921802C>T		Aradu.G83RZ-Aradu.A5UR9	
Aradu.A09	66932210	C	C	22	0.00	T	20	0.85	-0.85	0.45	-0.45	0.60	-0.60	intergenic_region	MODIFIER	n.66932210C>T		Aradu.G83RZ-Aradu.A5UR9	
Aradu.A09	66935969	A	A	17	0.00	T	21	1.00	-1.00	0.47	-0.47	0.65	-0.65	downstream_gene_variant	MODIFIER	c.*4577T>A		Aradu.A5UR9	histone-lysine N-methyltransferase SUVR2-like isoform X2 [Glycine max]%3B IPR001214 (SET domain)%2C IPR007728 (Pre-SET domain)%2C IPR018848 (WIYLD domain)%2C IPR025776 (Histone-lysine N-methyltransferase SUVR1/2/4)%3B GO:0005515 (protein binding)%2C GO:0005634 (nucleus)%2C GO:0008270 (zinc ion binding)%2C GO:0018024 (histone-lysine N-methyltransferase activity)%2C GO:0034968 (histone lysine methylation)
Aradu.A09	66940326	T	T	24	0.17	A	16	0.81	-0.64	0.50	-0.50	0.63	-0.63	downstream_gene_variant	MODIFIER	c.*220T>A		Aradu.A5UR9	histone-lysine N-methyltransferase SUVR2-like isoform X2 [Glycine max]%3B IPR001214 (SET domain)%2C IPR007728 (Pre-SET domain)%2C IPR018848 (WIYLD domain)%2C IPR025776 (Histone-lysine N-methyltransferase SUVR1/2/4)%3B GO:0005515 (protein binding)%2C GO:0005634 (nucleus)%2C GO:0008270 (zinc ion binding)%2C GO:0018024 (histone-lysine N-methyltransferase activity)%2C GO:0034968 (histone lysine methylation)

Aradu.A09	66949737	G	G	17	0.00	A	26	0.73	-0.73	0.47	-0.47	0.65	-0.65	missense_variant	MODERATE	c.1723C>T	p.Arg575Trp	Aradu.A5UR9	histone-lysine N-methyltransferase SUVR2-like isoform X2 [Glycine max]%3B IPR001214 (SET domain)%2C IPR007728 (Pre-SET domain)%2C IPR018848 (WIYLD domain)%2C IPR025776 (Histone-lysine N-methyltransferase SUVR1/2/4)%3B GO:0005515 (protein binding)%2C GO:0005634 (nucleus)%2C GO:0008270 (zinc ion binding)%2C GO:0018024 (histone-lysine N-methyltransferase activity)%2C GO:0034968 (histone lysine methylation)
Aradu.A09	66963899	C	C	35	0.09	G	18	0.94	-0.85	0.50	-0.50	0.61	-0.61	intergenic_region	MODIFIER	n.66963899C>G		Aradu.A5UR9-Aradu.IB7ZX	
Aradu.A09	66963969	C	C	32	0.09	T	23	0.86	-0.77	0.43	-0.43	0.57	-0.61	intergenic_region	MODIFIER	n.66963969C>T		Aradu.A5UR9-Aradu.IB7ZX	
Aradu.A09	66965819	T	T	23	0.04	C	24	0.70	-0.66	0.43	-0.43	0.57	-0.61	intergenic_region	MODIFIER	n.66965819C>T		Aradu.A5UR9-Aradu.IB7ZX	
Aradu.A09	66990637	G	G	20	0.05	A	36	0.83	-0.78	0.45	-0.45	0.60	-0.60	intergenic_region	MODIFIER	n.66990637G>A		Aradu.A5UR9-Aradu.IB7ZX	
Aradu.A09	67001812	C	C	37	0.08	G	19	0.73	-0.65	0.47	-0.47	0.63	-0.63	intergenic_region	MODIFIER	n.67001812G>C		Aradu.A5UR9-Aradu.IB7ZX	
Aradu.A09	67006542	G	G	17	0.00	A	10	1.00	-1.00	0.50	-0.60	0.70	-0.70	intergenic_region	MODIFIER	n.67006542G>A		Aradu.A5UR9-Aradu.IB7ZX	
Aradu.A09	67010920	G	G	24	0.08	A	19	0.84	-0.76	0.47	-0.47	0.63	-0.63	intergenic_region	MODIFIER	n.67010920G>A		Aradu.A5UR9-Aradu.IB7ZX	
Aradu.A09	67011914	T	T	21	0.19	G	22	0.81	-0.62	0.48	-0.48	0.62	-0.62	intergenic_region	MODIFIER	n.67011914G>T		Aradu.A5UR9-Aradu.IB7ZX	
Aradu.A09	67048369	C	C	21	0.00	T	20	1.00	-1.00	0.45	-0.45	0.60	-0.60	intergenic_region	MODIFIER	n.67048369C>T		Aradu.A5UR9-Aradu.IB7ZX	
Aradu.A09	67051055	C	C	20	0.15	T	35	0.77	-0.62	0.45	-0.45	0.60	-0.60	intergenic_region	MODIFIER	n.67051055T>C		Aradu.A5UR9-Aradu.IB7ZX	
Aradu.A09	67062469	C	C	23	0.04	T	22	0.95	-0.91	0.45	-0.45	0.59	-0.59	intergenic_region	MODIFIER	n.67062469C>T		Aradu.A5UR9-Aradu.IB7ZX	
Aradu.A09	67066503	C	C	16	0.00	A	29	0.93	-0.93	0.50	-0.50	0.63	-0.63	intergenic_region	MODIFIER	n.67066503C>A		Aradu.A5UR9-Aradu.IB7ZX	
Aradu.A09	67067041	C	C	13	0.00	A	12	0.75	-0.75	0.50	-0.50	0.67	-0.67	intergenic_region	MODIFIER	n.67067041C>A		Aradu.A5UR9-Aradu.IB7ZX	
Aradu.A09	67086749	A	A	15	0.00	G	14	0.92	-0.92	0.50	-0.50	0.64	-0.64	intergenic_region	MODIFIER	n.67086749G>A		Aradu.A5UR9-Aradu.IB7ZX	
Aradu.A09	67091872	T	T	30	0.07	C	24	0.75	-0.68	0.46	-0.46	0.54	-0.58	intergenic_region	MODIFIER	n.67091872C>T		Aradu.A5UR9-Aradu.IB7ZX	
Aradu.A09	67109185	A	A	30	0.07	G	19	0.94	-0.87	0.47	-0.47	0.63	-0.63	intergenic_region	MODIFIER	n.67109185A>G		Aradu.A5UR9-Aradu.IB7ZX	
Aradu.A09	67111152	C	C	11	0.09	T	22	0.86	-0.77	0.55	-0.55	0.64	-0.73	intergenic_region	MODIFIER	n.67111152T>C		Aradu.A5UR9-Aradu.IB7ZX	
Aradu.A09	67117151	C	C	24	0.00	T	22	1.00	-1.00	0.45	-0.45	0.59	-0.59	intergenic_region	MODIFIER	n.67117151C>T		Aradu.A5UR9-Aradu.IB7ZX	
Aradu.A09	67135861	G	G	19	0.21	T	15	0.93	-0.72	0.53	-0.47	0.67	-0.67	intergenic_region	MODIFIER	n.67135861G>T		Aradu.A5UR9-Aradu.IB7ZX	
Aradu.A09	67148541	G	G	25	0.08	T	26	1.00	-0.92	0.44	-0.44	0.56	-0.56	intergenic_region	MODIFIER	n.67148541G>T		Aradu.A5UR9-Aradu.IB7ZX	
Aradu.A09	67159775	T	T	14	0.07	G	34	1.00	-0.93	0.50	-0.50	0.64	-0.64	intergenic_region	MODIFIER	n.67159775T>G		Aradu.A5UR9-Aradu.IB7ZX	
Aradu.A09	67178812	C	C	20	0.10	A	19	0.84	-0.74	0.47	-0.47	0.63	-0.63	downstream_gene_variant	MODIFIER	c.*3710G>T		Aradu.IB7ZX	Unknown protein
Aradu.A09	67187868	T	T	16	0.06	C	19	1.00	-0.94	0.50	-0.50	0.63	-0.63	upstream_gene_variant	MODIFIER	c.-1568G>A		Aradu.IB7ZX	Unknown protein
Aradu.A09	67189333	T	T	10	0.20	A	12	0.91	-0.71	0.50	-0.60	0.70	-0.70	upstream_gene_variant	MODIFIER	c.-3033A>T		Aradu.IB7ZX	Unknown protein
Aradu.A09	67190720	T	T	21	0.00	A	25	1.00	-1.00	0.48	-0.48	0.62	-0.62	upstream_gene_variant	MODIFIER	c.-4420T>A		Aradu.IB7ZX	Unknown protein
Aradu.A09	67195990	C	C	25	0.04	T	27	0.92	-0.88	0.44	-0.44	0.56	-0.56	intergenic_region	MODIFIER	n.67195990C>T		Aradu.IB7ZX-Aradu.1L4C6	
Aradu.A09	67208962	T	T	11	0.00	C	15	0.93	-0.93	0.55	-0.55	0.64	-0.73	intergenic_region	MODIFIER	n.67208962C>T		Aradu.IB7ZX-Aradu.1L4C6	
Aradu.A09	67211242	G	G	25	0.20	A	34	0.94	-0.74	0.44	-0.44	0.56	-0.56	intergenic_region	MODIFIER	n.67211242G>A		Aradu.IB7ZX-Aradu.1L4C6	
Aradu.A09	67219391	C	C	26	0.04	T	32	0.84	-0.80	0.42	-0.46	0.54	-0.58	intergenic_region	MODIFIER	n.67219391C>T		Aradu.IB7ZX-Aradu.1L4C6	
Aradu.A09	67227628	C	C	21	0.10	G	24	0.83	-0.73	0.48	-0.48	0.62	-0.62	intergenic_region	MODIFIER	n.67227628G>C		Aradu.IB7ZX-Aradu.1L4C6	
Aradu.A09	67245607	A	A	23	0.04	G	26	0.92	-0.88	0.43	-0.43	0.57	-0.61	intergenic_region	MODIFIER	n.67245607A>G		Aradu.IB7ZX-Aradu.1L4C6	
Aradu.A09	67247498	C	C	24	0.00	T	28	0.85	-0.85	0.46	-0.46	0.54	-0.58	intergenic_region	MODIFIER	n.67247498C>T		Aradu.IB7ZX-Aradu.1L4C6	
Aradu.A09	67256035	G	G	26	0.19	A	18	0.94	-0.75	0.50	-0.50	0.61	-0.61	intergenic_region	MODIFIER	n.67256035G>A		Aradu.IB7ZX-Aradu.1L4C6	
Aradu.A09	67256078	C	C	25	0.20	T	20	0.95	-0.75	0.45	-0.45	0.60	-0.60	intergenic_region	MODIFIER	n.67256078C>T		Aradu.IB7ZX-Aradu.1L4C6	
Aradu.A09	67263982	C	C	18	0.17	T	25	0.84	-0.67	0.50	-0.50	0.61	-0.61	intergenic_region	MODIFIER	n.67263982C>T		Aradu.IB7ZX-Aradu.1L4C6	
Aradu.A09	67269217	T	T	36	0.03	C	29	0.89	-0.86	0.45	-0.45	0.55	-0.55	intergenic_region	MODIFIER	n.67269217C>T		Aradu.IB7ZX-Aradu.1L4C6	
Aradu.A09	67277047	T	T	19	0.16	C	17	0.82	-0.66	0.47	-0.47	0.65	-0.65	intergenic_region	MODIFIER	n.67277047C>T		Aradu.IB7ZX-Aradu.1L4C6	
Aradu.A09	67316665	G	G	19	0.11	T	27	0.96	-0.85	0.47	-0.47	0.63	-0.63	intergenic_region	MODIFIER	n.67316665G>T		Aradu.IB7ZX-Aradu.1L4C6	
Aradu.A09	67336795	G	G	11	0.09	C	23	1.00	-0.91	0.55	-0.55	0.64	-0.73	intergenic_region	MODIFIER	n.67336795G>C		Aradu.IB7ZX-Aradu.1L4C6	
Aradu.A09	67340256	T	T	18	0.00	C	26	0.76	-0.76	0.50	-0.50	0.61	-0.61	intergenic_region	MODIFIER	n.67340256C>T		Aradu.IB7ZX-Aradu.1L4C6	
Aradu.A09	67353851	C	C	13	0.15	T	11	1.00	-0.85	0.55	-0.55	0.64	-0.73	intergenic_region	MODIFIER	n.67353851C>T		Aradu.IB7ZX-Aradu.1L4C6	
Aradu.A09	67354665	C	C	21	0.00	A	28	0.82	-0.82	0.48	-0.48	0.62	-0.62	intergenic_region	MODIFIER	n.67354665C>A		Aradu.IB7ZX-Aradu.1L4C6	
Aradu.A09	67356696	A	A	42	0.05	G	23	1.00	-0.95	0.43	-0.43	0.57	-0.61	intergenic_region	MODIFIER	n.67356696G>A		Aradu.IB7ZX-Aradu.1L4C6	
Aradu.A09	67370331	T	T	23	0.09	C	39	0.76	-0.67	0.43	-0.43	0.57	-0.61	intergenic_region	MODIFIER	n.67370331C>T		Aradu.IB7ZX-Aradu.1L4C6	
Aradu.A09	67379593	C	C	25	0.12	T	26	0.92	-0.80	0.44	-0.44	0.56	-0.56	intergenic_region	MODIFIER	n.67379593C>T		Aradu.IB7ZX-Aradu.1L4C6	
Aradu.A09	67397743	A	A	19	0.05	G	17	0.94	-0.89	0.47	-0.47	0.65	-0.65	intergenic_region	MODIFIER	n.67397743G>A		Aradu.IB7ZX-Aradu.1L4C6	

Aradu.A09	67400631	A	A	22	0.05	G	26	0.92	-0.87	0.45	-0.45	0.59	-0.59	downstream_gene_variant	MODIFIER	c.*2294C>T	Aradu.1L4C6	oxysterol-binding protein-related protein 4B-like isoform X2 [Glycine max]
Aradu.A09	67402366	G	G	30	0.03	A	29	1.00	-0.97	0.45	-0.45	0.55	-0.55	downstream_gene_variant	MODIFIER	c.*559C>T	Aradu.1L4C6	oxysterol-binding protein-related protein 4B-like isoform X2 [Glycine max]
Aradu.A09	67408708	T	T	20	0.00	C	17	0.94	-0.94	0.47	-0.47	0.65	-0.65	upstream_gene_variant	MODIFIER	c.-3095G>A	Aradu.1L4C6	oxysterol-binding protein-related protein 4B-like isoform X2 [Glycine max]
Aradu.A09	67412562	T	T	45	0.11	A	26	0.73	-0.62	0.42	-0.46	0.54	-0.58	intergenic_region	MODIFIER	n.67412562A>T	Aradu.1L4C6-Aradu.24QXF	
Aradu.A09	67414242	A	A	21	0.00	T	27	0.77	-0.77	0.48	-0.48	0.62	-0.62	intergenic_region	MODIFIER	n.67414242A>T	Aradu.1L4C6-Aradu.24QXF	
Aradu.A09	67427913	G	G	23	0.09	A	24	0.83	-0.74	0.43	-0.43	0.57	-0.61	intergenic_region	MODIFIER	n.67427913G>A	Aradu.1L4C6-Aradu.24QXF	
Aradu.A09	67434458	G	G	18	0.00	C	22	0.81	-0.81	0.50	-0.50	0.61	-0.61	intergenic_region	MODIFIER	n.67434458G>C	Aradu.1L4C6-Aradu.24QXF	
Aradu.A09	67435142	G	G	22	0.09	A	15	0.80	-0.71	0.53	-0.47	0.67	-0.67	intergenic_region	MODIFIER	n.67435142G>A	Aradu.1L4C6-Aradu.24QXF	
Aradu.A09	67438593	G	G	22	0.00	C	20	0.85	-0.85	0.45	-0.45	0.60	-0.60	intergenic_region	MODIFIER	n.67438593C>G	Aradu.1L4C6-Aradu.24QXF	
Aradu.A09	67445237	A	A	17	0.12	G	27	0.81	-0.69	0.47	-0.47	0.65	-0.65	intergenic_region	MODIFIER	n.67445237A>G	Aradu.1L4C6-Aradu.24QXF	
Aradu.A09	67452623	G	G	15	0.00	C	11	0.81	-0.81	0.55	-0.55	0.64	-0.73	intergenic_region	MODIFIER	n.67452623G>C	Aradu.1L4C6-Aradu.24QXF	
Aradu.A09	67453305	C	C	13	0.15	T	19	1.00	-0.85	0.54	-0.54	0.62	-0.69	intergenic_region	MODIFIER	n.67453305C>T	Aradu.1L4C6-Aradu.24QXF	
Aradu.A09	67458202	A	A	37	0.14	T	30	0.73	-0.59	0.43	-0.43	0.57	-0.57	intergenic_region	MODIFIER	n.67458202T>A	Aradu.1L4C6-Aradu.24QXF	
Aradu.A09	67459242	T	T	25	0.36	C	39	0.94	-0.58	0.44	-0.44	0.56	-0.56	intergenic_region	MODIFIER	n.67459242C>T	Aradu.1L4C6-Aradu.24QXF	
Aradu.A09	67468365	G	G	18	0.11	A	26	0.84	-0.73	0.50	-0.50	0.61	-0.61	intergenic_region	MODIFIER	n.67468365G>A	Aradu.1L4C6-Aradu.24QXF	
Aradu.A09	67472158	T	T	38	0.05	C	30	0.73	-0.68	0.43	-0.43	0.57	-0.57	intergenic_region	MODIFIER	n.67472158C>T	Aradu.1L4C6-Aradu.24QXF	
Aradu.A09	67472762	T	T	28	0.14	C	23	0.87	-0.73	0.43	-0.43	0.57	-0.61	intergenic_region	MODIFIER	n.67472762T>C	Aradu.1L4C6-Aradu.24QXF	
Aradu.A09	67493224	T	T	27	0.00	C	26	0.96	-0.96	0.42	-0.46	0.54	-0.58	intergenic_region	MODIFIER	n.67493224C>T	Aradu.1L4C6-Aradu.24QXF	
Aradu.A09	67498986	C	C	16	0.00	T	23	0.91	-0.91	0.50	-0.50	0.63	-0.63	intergenic_region	MODIFIER	n.67498986C>T	Aradu.1L4C6-Aradu.24QXF	
Aradu.A09	67511441	C	C	17	0.00	T	18	0.94	-0.94	0.47	-0.47	0.65	-0.65	intergenic_region	MODIFIER	n.67511441C>T	Aradu.1L4C6-Aradu.24QXF	
Aradu.A09	67520721	A	A	23	0.09	G	21	1.00	-0.91	0.48	-0.48	0.62	-0.62	intergenic_region	MODIFIER	n.67520721A>G	Aradu.1L4C6-Aradu.24QXF	
Aradu.A09	67552722	C	C	22	0.09	A	10	1.00	-0.91	0.50	-0.60	0.70	-0.70	intergenic_region	MODIFIER	n.67552722C>A	Aradu.1L4C6-Aradu.24QXF	
Aradu.A09	67555647	T	T	12	0.00	C	11	1.00	-1.00	0.55	-0.55	0.64	-0.73	intergenic_region	MODIFIER	n.67555647C>T	Aradu.1L4C6-Aradu.24QXF	
Aradu.A09	67560684	C	C	17	0.06	T	21	0.95	-0.89	0.47	-0.47	0.65	-0.65	intergenic_region	MODIFIER	n.67560684C>T	Aradu.1L4C6-Aradu.24QXF	
Aradu.A09	67561298	G	G	18	0.00	A	26	0.80	-0.80	0.50	-0.50	0.61	-0.61	intergenic_region	MODIFIER	n.67561298A>G	Aradu.1L4C6-Aradu.24QXF	
Aradu.A09	67574218	C	C	33	0.12	T	28	0.92	-0.80	0.43	-0.43	0.57	-0.57	intergenic_region	MODIFIER	n.67574218C>T	Aradu.1L4C6-Aradu.24QXF	
Aradu.A09	67576789	A	A	17	0.00	G	21	1.00	-1.00	0.47	-0.47	0.65	-0.65	intergenic_region	MODIFIER	n.67576789G>A	Aradu.1L4C6-Aradu.24QXF	
Aradu.A09	67604144	G	G	27	0.07	A	21	0.80	-0.73	0.48	-0.48	0.62	-0.62	intergenic_region	MODIFIER	n.67604144G>A	Aradu.1L4C6-Aradu.24QXF	
Aradu.A09	67615836	G	G	39	0.03	A	30	0.80	-0.77	0.43	-0.43	0.57	-0.57	intergenic_region	MODIFIER	n.67615836G>A	Aradu.1L4C6-Aradu.24QXF	
Aradu.A09	67626465	A	A	20	0.05	G	24	1.00	-0.95	0.45	-0.45	0.60	-0.60	intergenic_region	MODIFIER	n.67626465G>A	Aradu.1L4C6-Aradu.24QXF	
Aradu.A09	67685704	C	C	20	0.00	A	25	1.00	-1.00	0.45	-0.45	0.60	-0.60	intergenic_region	MODIFIER	n.67685704C>A	Aradu.1L4C6-Aradu.24QXF	
Aradu.A09	67689285	T	T	18	0.06	A	11	1.00	-0.94	0.55	-0.55	0.64	-0.73	intergenic_region	MODIFIER	n.67689285T>A	Aradu.1L4C6-Aradu.24QXF	
Aradu.A09	67692800	A	A	21	0.14	C	20	0.80	-0.66	0.45	-0.45	0.60	-0.60	intergenic_region	MODIFIER	n.67692800C>A	Aradu.1L4C6-Aradu.24QXF	
Aradu.A09	67694010	C	C	19	0.05	A	13	1.00	-0.95	0.54	-0.54	0.62	-0.69	intergenic_region	MODIFIER	n.67694010C>A	Aradu.1L4C6-Aradu.24QXF	
Aradu.A09	67703288	C	C	32	0.06	T	31	0.64	-0.58	0.42	-0.42	0.55	-0.55	intergenic_region	MODIFIER	n.67703288C>T	Aradu.1L4C6-Aradu.24QXF	
Aradu.A09	67706202	G	G	12	0.00	T	19	0.94	-0.94	0.50	-0.50	0.67	-0.67	intergenic_region	MODIFIER	n.67706202G>T	Aradu.1L4C6-Aradu.24QXF	
Aradu.A09	67730743	T	T	21	0.05	C	15	0.93	-0.88	0.53	-0.47	0.67	-0.67	intergenic_region	MODIFIER	n.67730743C>T	Aradu.24QXF-Aradu.C6VT4	
Aradu.A09	67736289	C	C	21	0.00	T	22	0.77	-0.77	0.48	-0.48	0.62	-0.62	intergenic_region	MODIFIER	n.67736289C>T	Aradu.24QXF-Aradu.C6VT4	
Aradu.A09	67752457	C	C	33	0.03	T	32	0.81	-0.78	0.44	-0.44	0.56	-0.56	intergenic_region	MODIFIER	n.67752457C>T	Aradu.24QXF-Aradu.C6VT4	
Aradu.A09	67754108	C	C	29	0.07	G	37	0.94	-0.87	0.45	-0.45	0.55	-0.55	intergenic_region	MODIFIER	n.67754108C>G	Aradu.24QXF-Aradu.C6VT4	
Aradu.A09	67754130	A	A	29	0.07	G	30	0.93	-0.86	0.45	-0.45	0.55	-0.55	intergenic_region	MODIFIER	n.67754130G>A	Aradu.24QXF-Aradu.C6VT4	
Aradu.A09	67768035	C	C	25	0.00	G	21	0.76	-0.76	0.48	-0.48	0.62	-0.62	intergenic_region	MODIFIER	n.67768035G>C	Aradu.24QXF-Aradu.C6VT4	
Aradu.A09	67768926	C	C	23	0.04	A	19	0.94	-0.90	0.47	-0.47	0.63	-0.63	intergenic_region	MODIFIER	n.67768926C>A	Aradu.24QXF-Aradu.C6VT4	
Aradu.A09	67777024	T	T	14	0.07	C	29	0.86	-0.79	0.50	-0.50	0.64	-0.64	intergenic_region	MODIFIER	n.67777024T>C	Aradu.24QXF-Aradu.C6VT4	
Aradu.A09	67783669	C	C	19	0.00	A	26	0.96	-0.96	0.47	-0.47	0.63	-0.63	intergenic_region	MODIFIER	n.67783669C>A	Aradu.24QXF-Aradu.C6VT4	
Aradu.A09	67783678	A	A	19	0.00	G	25	0.96	-0.96	0.47	-0.47	0.63	-0.63	intergenic_region	MODIFIER	n.67783678G>A	Aradu.24QXF-Aradu.C6VT4	
Aradu.A09	67785833	G	G	15	0.00	T	22	0.95	-0.95	0.53	-0.47	0.67	-0.67	intergenic_region	MODIFIER	n.67785833G>T	Aradu.24QXF-Aradu.C6VT4	
Aradu.A09	67786402	T	T	16	0.00	A	22	1.00	-1.00	0.50	-0.50	0.63	-0.63	intergenic_region	MODIFIER	n.67786402T>A	Aradu.24QXF-Aradu.C6VT4	
Aradu.A09	67786466	T	T	25	0.00	A	18	1.00	-1.00	0.50	-0.50	0.61	-0.61	intergenic_region	MODIFIER	n.67786466T>A	Aradu.24QXF-Aradu.C6VT4	
Aradu.A09	67798289	C	C	18	0.00	A	15	1.00	-1.00	0.53	-0.47	0.67	-0.67	intergenic_region	MODIFIER	n.67798289C>A	Aradu.24QXF-Aradu.C6VT4	
Aradu.A09	67813194	C	C	16	0.12	T	22	0.81	-0.69	0.50	-0.50	0.63	-0.63	intergenic_region	MODIFIER	n.67813194C>T	Aradu.24QXF-Aradu.C6VT4	
Aradu.A09	67815769	A	A	20	0.00	G	24	0.75	-0.75	0.45	-0.45	0.60	-0.60	intergenic_region	MODIFIER	n.67815769A>G	Aradu.24QXF-Aradu.C6VT4	
Aradu.A09	67822816	G	G	18	0.06	A	15	0.80	-0.74	0.53	-0.47	0.67	-0.67	intergenic_region	MODIFIER	n.67822816G>A	Aradu.24QXF-Aradu.C6VT4	
Aradu.A09	67839681	C	C	23	0.09	T	23	0.91	-0.82	0.43	-0.43	0.57	-0.61	intergenic_region	MODIFIER	n.67839681C>T	Aradu.24QXF-Aradu.C6VT4	
Aradu.A09	67842680	G	G	20	0.05	A	18	0.94	-0.89	0.50	-0.50	0.61	-0.61	intergenic_region	MODIFIER	n.67842680G>A	Aradu.24QXF-Aradu.C6VT4	
Aradu.A09	67849608	C	C	12	0.00	T	11	1.00	-1.00	0.55	-0.55	0.64	-0.73	intergenic_region	MODIFIER	n.67849608C>T	Aradu.24QXF-Aradu.C6VT4	
Aradu.A09	67849909	C	C	15	0.07	A	18	0.77	-0.70	0.53	-0.47	0.67	-0.67	intergenic_region	MODIFIER	n.67849909C>A	Aradu.24QXF-Aradu.C6VT4	
Aradu.A09	67856309	C	C	17	0.12	A	27	0.85	-0.73	0.47	-0.47	0.65	-0.65	intergenic_region	MODIFIER	n.67856309C>A	Aradu.24QXF-Aradu.C6VT4	
Aradu.A09	67863283	C	C	35	0.06	A	25	0.64	-0.58	0.44	-0.44	0.56	-0.56	intergenic_region	MODIFIER	n.67863283C>A	Aradu.24QXF-Aradu.C6VT4	

Aradu.A09	67881146	G	G	23	0.22	A	22	1.00	-0.78	0.45	-0.45	0.59	-0.59	intergenic_region	MODIFIER	n.67881146G>A	Aradu.24QXF-Aradu.C6VT4	
Aradu.A09	67886290	C	C	17	0.18	T	26	0.92	-0.74	0.47	-0.47	0.65	-0.65	intergenic_region	MODIFIER	n.67886290C>T	Aradu.24QXF-Aradu.C6VT4	
Aradu.A09	67892067	G	G	20	0.00	A	20	0.80	-0.80	0.45	-0.45	0.60	-0.60	intergenic_region	MODIFIER	n.67892067G>A	Aradu.24QXF-Aradu.C6VT4	
Aradu.A09	67893381	G	G	28	0.07	A	27	0.77	-0.70	0.44	-0.44	0.56	-0.56	intergenic_region	MODIFIER	n.67893381G>A	Aradu.24QXF-Aradu.C6VT4	
Aradu.A09	68006955	C	C	34	0.09	A	20	0.95	-0.86	0.45	-0.45	0.60	-0.60	intergenic_region	MODIFIER	n.68006955C>A	Aradu.C6VT4-Aradu.Q64BX	
Aradu.A09	68013410	T	T	13	0.00	G	12	0.91	-0.91	0.50	-0.50	0.67	-0.67	intergenic_region	MODIFIER	n.68013410G>T	Aradu.C6VT4-Aradu.Q64BX	
Aradu.A09	68025079	C	C	33	0.06	T	11	1.00	-0.94	0.55	-0.55	0.64	-0.73	intergenic_region	MODIFIER	n.68025079T>C	Aradu.C6VT4-Aradu.Q64BX	
Aradu.A09	68031405	G	G	24	0.08	A	25	0.92	-0.84	0.46	-0.46	0.54	-0.58	intergenic_region	MODIFIER	n.68031405G>A	Aradu.C6VT4-Aradu.Q64BX	
Aradu.A09	68069046	G	G	20	0.00	T	29	0.72	-0.72	0.45	-0.45	0.60	-0.60	intergenic_region	MODIFIER	n.68069046G>T	Aradu.C6VT4-Aradu.Q64BX	
Aradu.A09	68079778	G	G	29	0.00	C	20	0.80	-0.80	0.45	-0.45	0.60	-0.60	intergenic_region	MODIFIER	n.68079778G>C	Aradu.C6VT4-Aradu.Q64BX	
Aradu.A09	68085778	G	G	23	0.00	A	26	0.96	-0.96	0.43	-0.43	0.57	-0.61	intergenic_region	MODIFIER	n.68085778G>A	Aradu.C6VT4-Aradu.Q64BX	
Aradu.A09	68096717	C	C	23	0.04	A	19	1.00	-0.96	0.47	-0.47	0.63	-0.63	intergenic_region	MODIFIER	n.68096717C>A	Aradu.C6VT4-Aradu.Q64BX	
Aradu.A09	68101677	G	G	13	0.00	A	16	0.87	-0.87	0.54	-0.54	0.62	-0.69	intergenic_region	MODIFIER	n.68101677G>A	Aradu.C6VT4-Aradu.Q64BX	
Aradu.A09	68112516	C	C	21	0.05	T	18	0.77	-0.72	0.50	-0.50	0.61	-0.61	intergenic_region	MODIFIER	n.68112516C>T	Aradu.C6VT4-Aradu.Q64BX	
Aradu.A09	68112800	G	G	32	0.06	A	25	0.96	-0.90	0.44	-0.44	0.56	-0.56	intergenic_region	MODIFIER	n.68112800G>A	Aradu.C6VT4-Aradu.Q64BX	
Aradu.A09	68116912	T	T	12	0.00	C	29	0.93	-0.93	0.50	-0.50	0.67	-0.67	intergenic_region	MODIFIER	n.68116912T>C	Aradu.C6VT4-Aradu.Q64BX	
Aradu.A09	68134256	G	G	27	0.04	T	16	0.68	-0.64	0.50	-0.50	0.63	-0.63	intergenic_region	MODIFIER	n.68134256G>T	Aradu.C6VT4-Aradu.Q64BX	
Aradu.A09	68141143	A	A	18	0.06	C	14	1.00	-0.94	0.50	-0.50	0.64	-0.64	intergenic_region	MODIFIER	n.68141143A>C	Aradu.C6VT4-Aradu.Q64BX	
Aradu.A09	68143067	T	T	19	0.26	G	24	1.00	-0.74	0.47	-0.47	0.63	-0.63	intergenic_region	MODIFIER	n.68143067T>G	Aradu.C6VT4-Aradu.Q64BX	
Aradu.A09	68143344	T	T	16	0.25	C	30	0.93	-0.68	0.50	-0.50	0.63	-0.63	intergenic_region	MODIFIER	n.68143344C>T	Aradu.C6VT4-Aradu.Q64BX	
Aradu.A09	68163108	G	G	19	0.00	A	24	1.00	-1.00	0.47	-0.47	0.63	-0.63	intergenic_region	MODIFIER	n.68163108G>A	Aradu.C6VT4-Aradu.Q64BX	
Aradu.A09	68177536	A	A	21	0.00	G	18	1.00	-1.00	0.50	-0.50	0.61	-0.61	intergenic_region	MODIFIER	n.68177536G>A	Aradu.C6VT4-Aradu.Q64BX	
Aradu.A09	68183063	C	C	21	0.00	T	15	0.93	-0.93	0.53	-0.47	0.67	-0.67	intergenic_region	MODIFIER	n.68183063C>T	Aradu.C6VT4-Aradu.Q64BX	
Aradu.A09	68202459	C	C	16	0.12	T	16	0.93	-0.81	0.50	-0.50	0.63	-0.63	intergenic_region	MODIFIER	n.68202459C>T	Aradu.C6VT4-Aradu.Q64BX	
Aradu.A09	68224225	G	G	13	0.15	T	18	0.94	-0.79	0.54	-0.54	0.62	-0.69	intergenic_region	MODIFIER	n.68224225G>T	Aradu.C6VT4-Aradu.Q64BX	
Aradu.A09	68226523	C	C	16	0.00	T	16	0.93	-0.93	0.50	-0.50	0.63	-0.63	intergenic_region	MODIFIER	n.68226523C>T	Aradu.C6VT4-Aradu.Q64BX	
Aradu.A09	68236840	T	T	22	0.09	G	22	0.77	-0.68	0.45	-0.45	0.59	-0.59	intergenic_region	MODIFIER	n.68236840T>G	Aradu.C6VT4-Aradu.Q64BX	
Aradu.A09	68247500	T	T	25	0.08	A	29	1.00	-0.92	0.44	-0.44	0.56	-0.56	intergenic_region	MODIFIER	n.68247500A>T	Aradu.C6VT4-Aradu.Q64BX	
Aradu.A09	68251871	T	T	15	0.07	C	14	0.78	-0.71	0.50	-0.50	0.64	-0.64	intergenic_region	MODIFIER	n.68251871T>C	Aradu.C6VT4-Aradu.Q64BX	
Aradu.A09	68258096	G	G	26	0.04	T	21	0.95	-0.91	0.48	-0.48	0.62	-0.62	intergenic_region	MODIFIER	n.68258096G>T	Aradu.C6VT4-Aradu.Q64BX	
Aradu.A09	68269813	C	C	18	0.06	A	22	0.86	-0.80	0.50	-0.50	0.61	-0.61	intergenic_region	MODIFIER	n.68269813C>A	Aradu.C6VT4-Aradu.Q64BX	
Aradu.A09	68310469	G	G	34	0.06	A	15	0.93	-0.87	0.53	-0.47	0.67	-0.67	intergenic_region	MODIFIER	n.68310469G>A	Aradu.C6VT4-Aradu.Q64BX	
Aradu.A09	68319303	T	T	22	0.14	G	16	0.93	-0.79	0.50	-0.50	0.63	-0.63	intergenic_region	MODIFIER	n.68319303T>G	Aradu.C6VT4-Aradu.Q64BX	
Aradu.A09	68319439	A	A	17	0.12	G	13	1.00	-0.88	0.54	-0.54	0.62	-0.69	intergenic_region	MODIFIER	n.68319439A>G	Aradu.C6VT4-Aradu.Q64BX	
Aradu.A09	68327156	C	C	15	0.07	T	22	0.81	-0.74	0.53	-0.47	0.67	-0.67	intergenic_region	MODIFIER	n.68327156C>T	Aradu.C6VT4-Aradu.Q64BX	
Aradu.A09	68327657	C	C	17	0.23	T	21	1.00	-0.77	0.47	-0.47	0.65	-0.65	intergenic_region	MODIFIER	n.68327657C>T	Aradu.C6VT4-Aradu.Q64BX	
Aradu.A09	68342895	C	C	17	0.12	A	21	0.95	-0.83	0.47	-0.47	0.65	-0.65	downstream_gene_variant	MODIFIER	c.*4607C>A	Aradu.Q64BX	threonine aldolase 1%3B IPR015424 (Pyridoxal phosphate-dependent transferase)%2C IPR023603 (Threonine aldolase)%3B GO:0003824 (catalytic activity)%2C GO:0006520 (cellular amino acid metabolic process)%2C GO:0016829 (lyase activity)%2C GO:0030170 (pyridoxal phosphate binding)
Aradu.A09	68352803	C	C	17	0.00	A	22	0.81	-0.81	0.47	-0.47	0.65	-0.65	intergenic_region	MODIFIER	n.68352803C>A	Aradu.Q64BX-Aradu.G8368	
Aradu.A09	68353219	C	C	13	0.08	T	26	0.84	-0.76	0.54	-0.54	0.62	-0.69	intergenic_region	MODIFIER	n.68353219C>T	Aradu.Q64BX-Aradu.G8368	
Aradu.A09	68365300	A	A	16	0.00	G	11	1.00	-1.00	0.55	-0.55	0.64	-0.73	intergenic_region	MODIFIER	n.68365300A>G	Aradu.Q64BX-Aradu.G8368	
Aradu.A09	68366164	C	C	23	0.13	T	29	0.93	-0.80	0.43	-0.43	0.57	-0.61	intergenic_region	MODIFIER	n.68366164C>T	Aradu.Q64BX-Aradu.G8368	
Aradu.A09	68366991	C	C	28	0.07	A	16	0.81	-0.74	0.50	-0.50	0.63	-0.63	intergenic_region	MODIFIER	n.68366991C>A	Aradu.Q64BX-Aradu.G8368	
Aradu.A09	68374061	T	T	19	0.00	C	22	1.00	-1.00	0.47	-0.47	0.63	-0.63	intergenic_region	MODIFIER	n.68374061T>C	Aradu.Q64BX-Aradu.G8368	
Aradu.A09	68378541	C	C	25	0.00	T	20	0.95	-0.95	0.45	-0.45	0.60	-0.60	intergenic_region	MODIFIER	n.68378541C>T	Aradu.Q64BX-Aradu.G8368	
Aradu.A09	68382423	T	T	23	0.13	G	14	1.00	-0.87	0.50	-0.50	0.64	-0.64	intergenic_region	MODIFIER	n.68382423G>T	Aradu.Q64BX-Aradu.G8368	
Aradu.A09	68385327	C	C	18	0.17	T	33	0.96	-0.79	0.50	-0.50	0.61	-0.61	intergenic_region	MODIFIER	n.68385327C>T	Aradu.Q64BX-Aradu.G8368	
Aradu.A09	68389008	G	G	22	0.09	A	22	0.77	-0.68	0.45	-0.45	0.59	-0.59	intergenic_region	MODIFIER	n.68389008G>A	Aradu.Q64BX-Aradu.G8368	
Aradu.A09	68392750	C	C	19	0.16	A	17	0.82	-0.66	0.47	-0.47	0.65	-0.65	intergenic_region	MODIFIER	n.68392750C>A	Aradu.Q64BX-Aradu.G8368	
Aradu.A09	68400311	A	A	19	0.00	G	19	0.94	-0.94	0.47	-0.47	0.63	-0.63	intergenic_region	MODIFIER	n.68400311A>G	Aradu.Q64BX-Aradu.G8368	
Aradu.A09	68411177	G	G	17	0.18	A	16	0.81	-0.63	0.50	-0.50	0.63	-0.63	intergenic_region	MODIFIER	n.68411177G>A	Aradu.Q64BX-Aradu.G8368	
Aradu.A09	68425221	G	G	22	0.00	T	17	0.82	-0.82	0.47	-0.47	0.65	-0.65	intergenic_region	MODIFIER	n.68425221G>T	Aradu.Q64BX-Aradu.G8368	
Aradu.A09	68431818	C	C	12	0.00	A	24	0.91	-0.91	0.50	-0.50	0.67	-0.67	intergenic_region	MODIFIER	n.68431818C>A	Aradu.Q64BX-Aradu.G8368	
Aradu.A09	68434378	T	T	19	0.11	C	22	1.00	-0.89	0.47	-0.47	0.63	-0.63	intergenic_region	MODIFIER	n.68434378C>T	Aradu.Q64BX-Aradu.G8368	
Aradu.A09	68436044	G	G	22	0.22	T	28	0.82	-0.60	0.45	-0.45	0.59	-0.59	intergenic_region	MODIFIER	n.68436044G>T	Aradu.Q64BX-Aradu.G8368	
Aradu.A09	68439910	C	C	28	0.00	T	38	1.00	-1.00	0.43	-0.43	0.57	-0.57	intergenic_region	MODIFIER	n.68439910C>T	Aradu.Q64BX-Aradu.G8368	
Aradu.A09	68441783	T	T	30	0.00	G	27	1.00	-1.00	0.44	-0.44	0.56	-0.56	intergenic_region	MODIFIER	n.68441783G>T	Aradu.Q64BX-Aradu.G8368	

Aradu.A09	68468474	T	T	33	0.06	C	26	0.84	−0.78	0.42	−0.46	0.54	−0.58	intergenic_region	MODIFIER	n.68468474C>T	Aradu.Q64BX-Aradu.G8368	
Aradu.A09	68476952	T	T	25	0.12	C	27	0.85	−0.73	0.44	−0.44	0.56	−0.56	intergenic_region	MODIFIER	n.68476952C>T	Aradu.Q64BX-Aradu.G8368	
Aradu.A09	68479662	T	T	16	0.00	C	19	0.78	−0.78	0.50	−0.50	0.63	−0.63	intergenic_region	MODIFIER	n.68479662T>C	Aradu.Q64BX-Aradu.G8368	
Aradu.A09	68520977	C	C	22	0.09	T	26	0.92	−0.83	0.45	−0.45	0.59	−0.59	intergenic_region	MODIFIER	n.68520977C>T	Aradu.Q64BX-Aradu.G8368	
Aradu.A09	68523294	G	G	27	0.04	A	16	0.93	−0.89	0.50	−0.50	0.63	−0.63	intergenic_region	MODIFIER	n.68523294G>A	Aradu.Q64BX-Aradu.G8368	
Aradu.A09	68531382	C	C	22	0.05	T	22	0.95	−0.90	0.45	−0.45	0.59	−0.59	intergenic_region	MODIFIER	n.68531382C>T	Aradu.Q64BX-Aradu.G8368	
Aradu.A09	68531569	C	C	20	0.10	A	25	0.96	−0.86	0.45	−0.45	0.60	−0.60	intergenic_region	MODIFIER	n.68531569C>A	Aradu.Q64BX-Aradu.G8368	
Aradu.A09	68532033	C	C	24	0.00	T	22	0.95	−0.95	0.45	−0.45	0.59	−0.59	intergenic_region	MODIFIER	n.68532033C>T	Aradu.Q64BX-Aradu.G8368	
Aradu.A09	68538953	C	C	29	0.00	T	23	0.91	−0.91	0.43	−0.43	0.57	−0.61	intergenic_region	MODIFIER	n.68538953C>T	Aradu.Q64BX-Aradu.G8368	
Aradu.A09	68559953	C	C	27	0.07	T	19	0.94	−0.87	0.47	−0.47	0.63	−0.63	intergenic_region	MODIFIER	n.68559953C>T	Aradu.Q64BX-Aradu.G8368	
Aradu.A09	68563576	A	A	21	0.29	G	26	1.00	−0.71	0.48	−0.48	0.62	−0.62	intergenic_region	MODIFIER	n.68563576G>A	Aradu.Q64BX-Aradu.G8368	
Aradu.A09	68563599	T	T	25	0.24	C	28	1.00	−0.76	0.44	−0.44	0.56	−0.56	intergenic_region	MODIFIER	n.68563599C>T	Aradu.Q64BX-Aradu.G8368	
Aradu.A09	68564744	G	G	14	0.07	T	18	0.77	−0.70	0.50	−0.50	0.64	−0.64	intergenic_region	MODIFIER	n.68564744G>T	Aradu.Q64BX-Aradu.G8368	
Aradu.A09	68566427	C	C	20	0.00	T	15	1.00	−1.00	0.53	−0.47	0.67	−0.67	intergenic_region	MODIFIER	n.68566427C>T	Aradu.Q64BX-Aradu.G8368	
Aradu.A09	68568178	C	C	22	0.14	A	21	0.80	−0.66	0.48	−0.48	0.62	−0.62	intergenic_region	MODIFIER	n.68568178C>A	Aradu.Q64BX-Aradu.G8368	
Aradu.A09	68575314	T	T	20	0.05	C	19	1.00	−0.95	0.47	−0.47	0.63	−0.63	intergenic_region	MODIFIER	n.68575314C>T	Aradu.Q64BX-Aradu.G8368	
Aradu.A09	68579565	G	G	22	0.09	A	32	0.93	−0.84	0.45	−0.45	0.59	−0.59	intergenic_region	MODIFIER	n.68579565G>A	Aradu.Q64BX-Aradu.G8368	
Aradu.A09	68581050	G	G	27	0.04	T	29	0.79	−0.75	0.44	−0.44	0.56	−0.56	intergenic_region	MODIFIER	n.68581050G>T	Aradu.Q64BX-Aradu.G8368	
Aradu.A09	68584292	T	T	34	0.06	G	29	0.93	−0.87	0.45	−0.45	0.55	−0.55	intergenic_region	MODIFIER	n.68584292G>T	Aradu.Q64BX-Aradu.G8368	
Aradu.A09	68585218	C	C	20	0.10	A	23	0.78	−0.68	0.45	−0.45	0.60	−0.60	intergenic_region	MODIFIER	n.68585218C>A	Aradu.Q64BX-Aradu.G8368	
Aradu.A09	68590090	C	C	19	0.00	T	31	0.80	−0.80	0.47	−0.47	0.63	−0.63	intergenic_region	MODIFIER	n.68590090C>T	Aradu.Q64BX-Aradu.G8368	
Aradu.A09	68615308	C	C	26	0.08	T	28	0.78	−0.70	0.42	−0.46	0.54	−0.58	intergenic_region	MODIFIER	n.68615308C>T	Aradu.Q64BX-Aradu.G8368	
Aradu.A09	68632333	G	G	19	0.00	A	15	0.80	−0.80	0.53	−0.47	0.67	−0.67	intergenic_region	MODIFIER	n.68632333G>A	Aradu.Q64BX-Aradu.G8368	
Aradu.A09	68645171	C	C	33	0.18	T	24	0.79	−0.61	0.46	−0.46	0.54	−0.58	intergenic_region	MODIFIER	n.68645171C>T	Aradu.Q64BX-Aradu.G8368	
Aradu.A09	68657795	G	G	13	0.00	T	15	0.93	−0.93	0.54	−0.54	0.62	−0.69	intergenic_region	MODIFIER	n.68657795G>T	Aradu.Q64BX-Aradu.G8368	
Aradu.A09	68676285	A	A	14	0.00	G	19	0.78	−0.78	0.50	−0.50	0.64	−0.64	downstream_gene_variant	MODIFIER	c.*2632G>A	Aradu.G8368	Unknown protein
Aradu.A09	68685486	G	G	20	0.25	C	18	0.94	−0.69	0.50	−0.50	0.61	−0.61	intergenic_region	MODIFIER	n.68685486C>G	Aradu.G8368-Aradu.YTOWZ	
Aradu.A09	68694249	C	C	19	0.05	A	12	0.75	−0.70	0.50	−0.50	0.67	−0.67	intergenic_region	MODIFIER	n.68694249C>A	Aradu.G8368-Aradu.YTOWZ	
Aradu.A09	68695435	C	C	28	0.04	A	21	0.76	−0.72	0.48	−0.48	0.62	−0.62	intergenic_region	MODIFIER	n.68695435C>A	Aradu.G8368-Aradu.YTOWZ	
Aradu.A09	68704391	A	A	24	0.12	G	14	0.78	−0.66	0.50	−0.50	0.64	−0.64	intergenic_region	MODIFIER	n.68704391A>G	Aradu.G8368-Aradu.YTOWZ	
Aradu.A09	68724474	T	T	22	0.00	C	23	0.82	−0.82	0.45	−0.45	0.59	−0.59	intergenic_region	MODIFIER	n.68724474C>T	Aradu.G8368-Aradu.YTOWZ	
Aradu.A09	68731942	A	A	24	0.00	G	13	0.92	−0.92	0.54	−0.54	0.62	−0.69	intergenic_region	MODIFIER	n.68731942G>A	Aradu.G8368-Aradu.YTOWZ	
Aradu.A09	68733520	T	T	26	0.08	A	24	0.91	−0.83	0.46	−0.46	0.54	−0.58	intergenic_region	MODIFIER	n.68733520A>T	Aradu.G8368-Aradu.YTOWZ	
Aradu.A09	68740053	A	A	19	0.11	G	19	0.94	−0.83	0.47	−0.47	0.63	−0.63	intergenic_region	MODIFIER	n.68740053G>A	Aradu.G8368-Aradu.YTOWZ	
Aradu.A09	68748344	C	C	19	0.11	A	19	0.84	−0.73	0.47	−0.47	0.63	−0.63	intergenic_region	MODIFIER	n.68748344A>C	Aradu.G8368-Aradu.YTOWZ	
Aradu.A09	68763527	C	C	22	0.00	T	20	1.00	−1.00	0.45	−0.45	0.60	−0.60	intergenic_region	MODIFIER	n.68763527C>T	Aradu.G8368-Aradu.YTOWZ	
Aradu.A09	68771907	T	T	13	0.00	G	14	1.00	−1.00	0.54	−0.54	0.62	−0.69	intergenic_region	MODIFIER	n.68771907G>T	Aradu.G8368-Aradu.YTOWZ	
Aradu.A09	68775295	G	G	27	0.11	A	30	0.96	−0.85	0.44	−0.44	0.56	−0.56	intergenic_region	MODIFIER	n.68775295G>A	Aradu.G8368-Aradu.YTOWZ	
Aradu.A09	68799499	T	T	22	0.00	A	23	0.82	−0.82	0.45	−0.45	0.59	−0.59	intergenic_region	MODIFIER	n.68799499T>A	Aradu.G8368-Aradu.YTOWZ	
Aradu.A09	68801311	C	C	23	0.00	T	24	0.70	−0.70	0.43	−0.43	0.57	−0.61	intergenic_region	MODIFIER	n.68801311C>T	Aradu.G8368-Aradu.YTOWZ	
Aradu.A09	68803866	T	T	13	0.00	A	11	0.81	−0.81	0.55	−0.55	0.64	−0.73	intergenic_region	MODIFIER	n.68803866T>A	Aradu.G8368-Aradu.YTOWZ	
Aradu.A09	68824036	C	C	32	0.06	A	13	0.76	−0.70	0.54	−0.54	0.62	−0.69	intergenic_region	MODIFIER	n.68824036C>A	Aradu.YTOWZ-Aradu.DPOS5	
Aradu.A09	68827336	G	G	20	0.00	A	18	0.94	−0.94	0.50	−0.50	0.61	−0.61	intergenic_region	MODIFIER	n.68827336G>A	Aradu.YTOWZ-Aradu.DPOS5	
Aradu.A09	68837417	C	C	13	0.00	T	20	0.90	−0.90	0.54	−0.54	0.62	−0.69	intergenic_region	MODIFIER	n.68837417C>T	Aradu.YTOWZ-Aradu.DPOS5	
Aradu.A09	68858857	A	A	16	0.19	G	17	0.82	−0.63	0.50	−0.50	0.63	−0.63	intergenic_region	MODIFIER	n.68858857G>A	Aradu.DPOS5-Aradu.I3J28	
Aradu.A09	68861089	G	G	15	0.07	T	28	0.82	−0.75	0.53	−0.47	0.67	−0.67	intergenic_region	MODIFIER	n.68861089G>T	Aradu.DPOS5-Aradu.I3J28	
Aradu.A09	68862285	C	C	23	0.00	A	27	0.92	−0.92	0.43	−0.43	0.57	−0.61	intergenic_region	MODIFIER	n.68862285C>A	Aradu.DPOS5-Aradu.I3J28	
Aradu.A09	68862976	G	G	16	0.06	A	22	0.95	−0.89	0.50	−0.50	0.63	−0.63	intergenic_region	MODIFIER	n.68862976G>A	Aradu.DPOS5-Aradu.I3J28	
Aradu.A09	68872838	A	A	21	0.00	G	21	0.85	−0.85	0.48	−0.48	0.62	−0.62	intergenic_region	MODIFIER	n.68872838A>G	Aradu.DPOS5-Aradu.I3J28	
Aradu.A09	68885111	C	C	25	0.04	G	18	0.77	−0.73	0.50	−0.50	0.61	−0.61	intergenic_region	MODIFIER	n.68885111C>G	Aradu.DPOS5-Aradu.I3J28	
Aradu.A09	68890537	G	G	20	0.10	A	26	0.84	−0.74	0.45	−0.45	0.60	−0.60	intergenic_region	MODIFIER	n.68890537G>A	Aradu.DPOS5-Aradu.I3J28	
Aradu.A09	68892439	A	A	12	0.00	C	23	0.82	−0.82	0.50	−0.50	0.67	−0.67	intergenic_region	MODIFIER	n.68892439C>A	Aradu.DPOS5-Aradu.I3J28	
Aradu.A09	68895367	C	C	19	0.11	T	29	0.86	−0.75	0.47	−0.47	0.63	−0.63	intergenic_region	MODIFIER	n.68895367C>T	Aradu.DPOS5-Aradu.I3J28	
Aradu.A09	68895712	T	T	19	0.05	G	17	0.94	−0.89	0.47	−0.47	0.65	−0.65	intergenic_region	MODIFIER	n.68895712G>T	Aradu.DPOS5-Aradu.I3J28	
Aradu.A09	68903066	T	T	26	0.08	C	27	0.85	−0.77	0.42	−0.46	0.54	−0.58	intergenic_region	MODIFIER	n.68903066T>C	Aradu.DPOS5-Aradu.I3J28	
Aradu.A09	68915978	G	G	17	0.00	T	25	0.80	−0.80	0.47	−0.47	0.65	−0.65	intergenic_region	MODIFIER	n.68915978G>T	Aradu.DPOS5-Aradu.I3J28	
Aradu.A09	68926282	C	C	20	0.05	T	11	1.00	−0.95	0.55	−0.55	0.64	−0.73	intergenic_region	MODIFIER	n.68926282C>T	Aradu.DPOS5-Aradu.I3J28	
Aradu.A09	68939121	T	T	21	0.10	G	14	0.78	−0.68	0.50	−0.50	0.64	−0.64	intergenic_region	MODIFIER	n.68939121G>T	Aradu.DPOS5-Aradu.I3J28	
Aradu.A09	68954230	C	C	20	0.30	G	29	0.93	−0.63	0.45	−0.45	0.60	−0.60	intergenic_region	MODIFIER	n.68954230C>G	Aradu.DPOS5-Aradu.I3J28	
Aradu.A09	68958390	C	C	23	0.17	T	15	0.87	−0.70	0.53	−0.47	0.67	−0.67	intergenic_region	MODIFIER	n.68958390C>T	Aradu.DPOS5-Aradu.I3J28	
Aradu.A09	68959338	C	C	27	0.04	A	23	1.00	−0.96	0.43	−0.43	0.57	−0.61	intergenic_region	MODIFIER	n.68959338C>A	Aradu.DPOS5-Aradu.I3J28	
Aradu.A09	68975069	G	G	17	0.12	A	18	0.94	−0.82	0.47	−0.47	0.65	−0.65	intergenic_region	MODIFIER	n.68975069G>A	Aradu.DPOS5-Aradu.I3J28	
Aradu.A09	68983184	C	C	16	0.00	T	27	0.96	−0.96	0.50	−0.50	0.63	−0.63	intergenic_region	MODIFIER	n.68983184C>T	Aradu.DPOS5-Aradu.I3J28	
Aradu.A09	68987284	T	T	14	0.00	C	30	0.86	−0.86	0.50	−0.50	0.64	−0.64	intergenic_region	MODIFIER	n.68987284T>C	Aradu.DPOS5-Aradu.I3J28	
Aradu.A09	68989373	A	A	19	0.05	G	18	1.00	−0.95	0.50	−0.50	0.61	−0.61	intergenic_region	MODIFIER	n.68989373G>A	Aradu.DPOS5-Aradu.I3J28	
Aradu.A09	68996197	C	C	17	0.00	T	21	1.00	−1.00	0.47	−0.47	0.65	−0.65	intergenic_region	MODIFIER	n.68996197C>T	Aradu.DPOS5-Aradu.I3J28	
Aradu.A09	68999445	C	C	35	0.17	A	11	1.00	−0.83	0.55	−0.55	0.64	−0.73	intergenic_region	MODIFIER	n.68999445C>A	Aradu.DPOS5-Aradu.I3J28	
Aradu.A09	69007381	C	C	29	0.07	G	20	1.00	−0.93	0.45	−0.45	0.60	−0.60	intergenic_region	MODIFIER	n.69007381C>G	Aradu.DPOS5-Aradu.I3J28	

Aradu.A09	69013571	A	A	25	0.08	G	12	0.91	−0.83	0.50	−0.50	0.67	−0.67	intergenic_region	MODIFIER	n.69013571G>A	Aradu.DP0S5-Aradu.I3J28	
Aradu.A09	69025510	T	T	17	0.12	C	10	0.90	−0.78	0.50	−0.60	0.70	−0.70	intergenic_region	MODIFIER	n.69025510C>T	Aradu.DP0S5-Aradu.I3J28	
Aradu.A09	69034675	C	C	16	0.12	T	20	1.00	−0.88	0.50	−0.50	0.63	−0.63	intergenic_region	MODIFIER	n.69034675C>T	Aradu.DP0S5-Aradu.I3J28	
Aradu.A09	69037237	G	G	20	0.15	T	12	1.00	−0.85	0.50	−0.50	0.67	−0.67	intergenic_region	MODIFIER	n.69037237G>T	Aradu.DP0S5-Aradu.I3J28	
Aradu.A09	69045757	A	A	14	0.07	C	24	0.83	−0.76	0.50	−0.50	0.64	−0.64	intergenic_region	MODIFIER	n.69045757C>A	Aradu.DP0S5-Aradu.I3J28	
Aradu.A09	69050499	G	G	21	0.14	A	24	1.00	−0.86	0.48	−0.48	0.62	−0.62	intergenic_region	MODIFIER	n.69050499G>A	Aradu.DP0S5-Aradu.I3J28	
Aradu.A09	69052622	C	C	15	0.13	T	28	0.82	−0.69	0.53	−0.47	0.67	−0.67	intergenic_region	MODIFIER	n.69052622C>T	Aradu.DP0S5-Aradu.I3J28	
Aradu.A09	69053720	C	C	30	0.00	T	24	0.70	−0.70	0.46	−0.46	0.54	−0.58	intergenic_region	MODIFIER	n.69053720C>T	Aradu.DP0S5-Aradu.I3J28	
Aradu.A09	69076871	C	C	24	0.08	T	23	0.82	−0.74	0.43	−0.43	0.57	−0.61	intergenic_region	MODIFIER	n.69076871T>C	Aradu.DP0S5-Aradu.I3J28	
Aradu.A09	69083072	A	A	15	0.00	C	16	0.93	−0.93	0.53	−0.47	0.67	−0.67	intergenic_region	MODIFIER	n.69083072C>A	Aradu.DP0S5-Aradu.I3J28	
Aradu.A09	69087457	C	C	19	0.05	T	23	0.82	−0.77	0.47	−0.47	0.63	−0.63	intergenic_region	MODIFIER	n.69087457C>T	Aradu.DP0S5-Aradu.I3J28	
Aradu.A09	69096506	G	G	17	0.06	A	25	0.84	−0.78	0.47	−0.47	0.65	−0.65	downstream_gene_variant	MODIFIER	c.*3602C>T	Aradu.I3J28	RNA-binding protein 1%3B IPR012677 (Nucleotide-binding%2C alpha-beta plait)%3B GO:0000166 (nucleotide binding)%2C GO:0003676 (nucleic acid binding)
Aradu.A09	69098176	A	A	13	0.00	G	18	1.00	−1.00	0.54	−0.54	0.62	−0.69	downstream_gene_variant	MODIFIER	c.*1932T>C	Aradu.I3J28	RNA-binding protein 1%3B IPR012677 (Nucleotide-binding%2C alpha-beta plait)%3B GO:0000166 (nucleotide binding)%2C GO:0003676 (nucleic acid binding)
Aradu.A09	69099105	C	C	27	0.04	A	35	0.97	−0.93	0.44	−0.44	0.56	−0.56	downstream_gene_variant	MODIFIER	c.*1003G>T	Aradu.I3J28	RNA-binding protein 1%3B IPR012677 (Nucleotide-binding%2C alpha-beta plait)%3B GO:0000166 (nucleotide binding)%2C GO:0003676 (nucleic acid binding)
Aradu.A09	69126683	G	G	20	0.20	T	16	1.00	−0.80	0.50	−0.50	0.63	−0.63	intergenic_region	MODIFIER	n.69126683G>T	Aradu.I3J28-Aradu.E3GVW	
Aradu.A09	69133128	G	G	16	0.06	A	19	0.84	−0.78	0.50	−0.50	0.63	−0.63	intergenic_region	MODIFIER	n.69133128G>A	Aradu.I3J28-Aradu.E3GVW	
Aradu.A09	69141350	C	C	13	0.08	T	25	0.92	−0.84	0.54	−0.54	0.62	−0.69	intergenic_region	MODIFIER	n.69141350C>T	Aradu.I3J28-Aradu.E3GVW	
Aradu.A09	69141823	T	T	38	0.11	C	22	0.90	−0.79	0.45	−0.45	0.59	−0.59	intergenic_region	MODIFIER	n.69141823C>T	Aradu.I3J28-Aradu.E3GVW	
Aradu.A09	69169108	G	G	28	0.04	T	14	0.92	−0.88	0.50	−0.50	0.64	−0.64	intergenic_region	MODIFIER	n.69169108G>T	Aradu.I3J28-Aradu.E3GVW	
Aradu.A09	69171326	G	G	30	0.00	A	18	0.77	−0.77	0.50	−0.50	0.61	−0.61	intergenic_region	MODIFIER	n.69171326G>A	Aradu.I3J28-Aradu.E3GVW	
Aradu.A09	69173630	T	T	14	0.00	C	24	0.79	−0.79	0.50	−0.50	0.64	−0.64	intergenic_region	MODIFIER	n.69173630C>T	Aradu.I3J28-Aradu.E3GVW	
Aradu.A09	69174286	A	A	20	0.00	T	17	0.94	−0.94	0.47	−0.47	0.65	−0.65	intergenic_region	MODIFIER	n.69174286A>T	Aradu.I3J28-Aradu.E3GVW	
Aradu.A09	69175812	C	C	21	0.00	A	29	0.79	−0.79	0.48	−0.48	0.62	−0.62	intergenic_region	MODIFIER	n.69175812C>A	Aradu.I3J28-Aradu.E3GVW	
Aradu.A09	69177764	T	T	28	0.07	G	24	0.75	−0.68	0.46	−0.46	0.54	−0.58	intergenic_region	MODIFIER	n.69177764T>G	Aradu.I3J28-Aradu.E3GVW	
Aradu.A09	69181847	G	G	11	0.00	A	12	1.00	−1.00	0.55	−0.55	0.64	−0.73	intergenic_region	MODIFIER	n.69181847G>A	Aradu.I3J28-Aradu.E3GVW	
Aradu.A09	69186706	T	T	13	0.00	C	15	1.00	−1.00	0.54	−0.54	0.62	−0.69	intergenic_region	MODIFIER	n.69186706C>T	Aradu.I3J28-Aradu.E3GVW	
Aradu.A09	69191206	T	T	15	0.07	A	20	1.00	−0.93	0.53	−0.47	0.67	−0.67	upstream_gene_variant	MODIFIER	c.-2506T>A	Aradu.E3GVW	Unknown protein
Aradu.A09	69200342	C	C	23	0.04	T	37	0.94	−0.90	0.43	−0.43	0.57	−0.61	intergenic_region	MODIFIER	n.69200342C>T	Aradu.E3GVW-Aradu.14TMT	
Aradu.A09	69200820	G	G	29	0.00	A	25	0.68	−0.68	0.44	−0.44	0.56	−0.56	intergenic_region	MODIFIER	n.69200820G>A	Aradu.E3GVW-Aradu.14TMT	
Aradu.A09	69216364	T	T	21	0.00	C	26	0.84	−0.84	0.48	−0.48	0.62	−0.62	intergenic_region	MODIFIER	n.69216364C>T	Aradu.E3GVW-Aradu.14TMT	
Aradu.A09	69225461	C	C	30	0.10	T	24	0.70	−0.60	0.46	−0.46	0.54	−0.58	intergenic_region	MODIFIER	n.69225461C>T	Aradu.E3GVW-Aradu.14TMT	
Aradu.A09	69225800	G	G	15	0.00	A	13	1.00	−1.00	0.54	−0.54	0.62	−0.69	intergenic_region	MODIFIER	n.69225800G>A	Aradu.E3GVW-Aradu.14TMT	
Aradu.A09	69225802	A	A	15	0.00	T	13	1.00	−1.00	0.54	−0.54	0.62	−0.69	intergenic_region	MODIFIER	n.69225802A>T	Aradu.E3GVW-Aradu.14TMT	
Aradu.A09	69271008	G	G	19	0.00	A	29	0.93	−0.93	0.47	−0.47	0.63	−0.63	intergenic_region	MODIFIER	n.69271008G>A	Aradu.E3GVW-Aradu.14TMT	
Aradu.A09	69271155	G	G	19	0.11	A	19	1.00	−0.89	0.47	−0.47	0.63	−0.63	intergenic_region	MODIFIER	n.69271155G>A	Aradu.E3GVW-Aradu.14TMT	
Aradu.A09	69273246	C	C	14	0.00	T	22	0.86	−0.86	0.50	−0.50	0.64	−0.64	intergenic_region	MODIFIER	n.69273246C>T	Aradu.E3GVW-Aradu.14TMT	
Aradu.A09	69282903	G	G	23	0.09	A	16	0.93	−0.84	0.50	−0.50	0.63	−0.63	intergenic_region	MODIFIER	n.69282903G>A	Aradu.E3GVW-Aradu.14TMT	
Aradu.A09	69297395	C	C	20	0.00	A	19	0.94	−0.94	0.47	−0.47	0.63	−0.63	intergenic_region	MODIFIER	n.69297395C>A	Aradu.E3GVW-Aradu.14TMT	
Aradu.A09	69307251	T	T	13	0.00	C	29	0.96	−0.96	0.54	−0.54	0.62	−0.69	intergenic_region	MODIFIER	n.69307251T>C	Aradu.E3GVW-Aradu.14TMT	
Aradu.A09	69308359	T	T	11	0.00	C	21	0.95	−0.95	0.55	−0.55	0.64	−0.73	intergenic_region	MODIFIER	n.69308359C>T	Aradu.E3GVW-Aradu.14TMT	
Aradu.A09	69321550	C	C	12	0.00	T	25	0.80	−0.80	0.50	−0.50	0.67	−0.67	intergenic_region	MODIFIER	n.69321550C>T	Aradu.E3GVW-Aradu.14TMT	
Aradu.A09	69321698	G	G	22	0.00	A	30	0.90	−0.90	0.45	−0.45	0.59	−0.59	intergenic_region	MODIFIER	n.69321698G>A	Aradu.E3GVW-Aradu.14TMT	
Aradu.A09	69344132	T	T	18	0.00	G	15	0.93	−0.93	0.53	−0.47	0.67	−0.67	intergenic_region	MODIFIER	n.69344132G>T	Aradu.E3GVW-Aradu.14TMT	
Aradu.A09	69352243	C	C	30	0.03	A	23	0.86	−0.83	0.43	−0.43	0.57	−0.61	intergenic_region	MODIFIER	n.69352243C>A	Aradu.E3GVW-Aradu.14TMT	
Aradu.A09	69367894	A	A	14	0.00	G	13	0.92	−0.92	0.54	−0.54	0.62	−0.69	intergenic_region	MODIFIER	n.69367894A>G	Aradu.E3GVW-Aradu.14TMT	
Aradu.A09	69381645	C	C	24	0.08	T	29	0.93	−0.85	0.46	−0.46	0.54	−0.58	intergenic_region	MODIFIER	n.69381645C>T	Aradu.E3GVW-Aradu.14TMT	
Aradu.A09	69395386	C	C	33	0.06	T	18	0.83	−0.77	0.50	−0.50	0.61	−0.61	intergenic_region	MODIFIER	n.69395386C>T	Aradu.E3GVW-Aradu.14TMT	
Aradu.A09	69396147	G	G	11	0.00	A	14	0.92	−0.92	0.55	−0.55	0.64	−0.73	intergenic_region	MODIFIER	n.69396147G>A	Aradu.E3GVW-Aradu.14TMT	
Aradu.A09	69415358	G	G	27	0.04	A	25	0.96	−0.92	0.44	−0.44	0.56	−0.56	intergenic_region	MODIFIER	n.69415358G>A	Aradu.14TMT-Aradu.V6J08	
Aradu.A09	69426680	A	A	21	0.05	G	18	0.83	−0.78	0.50	−0.50	0.61	−0.61	intergenic_region	MODIFIER	n.69426680G>A	Aradu.14TMT-Aradu.V6J08	

Aradu.A09	69428554	C	C	16	0.12	T	14	0.92	-0.80	0.50	-0.50	0.64	-0.64	intergenic_region	MODIFIER	n.69428554C>T	Aradu.14TMT-Aradu.V6J08	
Aradu.A09	69435304	A	A	32	0.03	T	15	0.93	-0.90	0.53	-0.47	0.67	-0.67	intergenic_region	MODIFIER	n.69435304A>T	Aradu.14TMT-Aradu.V6J08	
Aradu.A09	69436924	C	C	16	0.00	A	34	0.73	-0.73	0.50	-0.50	0.63	-0.63	intergenic_region	MODIFIER	n.69436924C>A	Aradu.14TMT-Aradu.V6J08	
Aradu.A09	69437380	A	A	13	0.00	C	11	0.81	-0.81	0.55	-0.55	0.64	-0.73	intergenic_region	MODIFIER	n.69437380A>C	Aradu.14TMT-Aradu.V6J08	
Aradu.A09	69445225	T	T	25	0.12	A	23	0.91	-0.79	0.43	-0.43	0.57	-0.61	intergenic_region	MODIFIER	n.69445225T>A	Aradu.14TMT-Aradu.V6J08	
Aradu.A09	69446671	A	A	20	0.20	G	19	1.00	-0.80	0.47	-0.47	0.63	-0.63	intergenic_region	MODIFIER	n.69446671G>A	Aradu.14TMT-Aradu.V6J08	
Aradu.A09	69450089	G	G	21	0.05	T	25	0.76	-0.71	0.48	-0.48	0.62	-0.62	intergenic_region	MODIFIER	n.69450089G>T	Aradu.14TMT-Aradu.V6J08	
Aradu.A09	69450092	G	G	22	0.00	T	26	0.76	-0.76	0.45	-0.45	0.59	-0.59	intergenic_region	MODIFIER	n.69450092G>T	Aradu.14TMT-Aradu.V6J08	
Aradu.A09	69450213	C	C	26	0.00	T	29	0.72	-0.72	0.42	-0.46	0.54	-0.58	intergenic_region	MODIFIER	n.69450213C>T	Aradu.14TMT-Aradu.V6J08	
Aradu.A09	69451548	A	A	15	0.00	G	22	0.77	-0.77	0.53	-0.47	0.67	-0.67	intergenic_region	MODIFIER	n.69451548G>A	Aradu.14TMT-Aradu.V6J08	
Aradu.A09	69457641	G	G	12	0.00	A	12	1.00	-1.00	0.50	-0.50	0.67	-0.67	intergenic_region	MODIFIER	n.69457641G>A	Aradu.14TMT-Aradu.V6J08	
Aradu.A09	69464498	A	A	14	0.00	G	23	0.82	-0.82	0.50	-0.50	0.64	-0.64	intergenic_region	MODIFIER	n.69464498G>A	Aradu.14TMT-Aradu.V6J08	
Aradu.A09	69476553	G	G	26	0.00	A	39	0.84	-0.84	0.42	-0.46	0.54	-0.58	intergenic_region	MODIFIER	n.69476553G>A	Aradu.14TMT-Aradu.V6J08	
Aradu.A09	69499225	T	T	20	0.00	C	11	1.00	-1.00	0.55	-0.55	0.64	-0.73	intergenic_region	MODIFIER	n.69499225T>C	Aradu.14TMT-Aradu.V6J08	
Araip.B02	5871612	T	T	32	0.03	C	22	0.77	-0.74	0.45	-0.45	0.59	-0.59	intergenic_region	MODIFIER	n.5871612C>T	Araip.G7SZ3-Araip.U3EQS	
Araip.B02	5877115	T	T	20	0.10	C	17	0.76	-0.66	0.47	-0.47	0.65	-0.65	downstream_gene_variant	MODIFIER	c.*4843A>G	Araip.U3EQS	protein IQ-DOMAIN 1-like isoform X3 [Glycine max]%3B IPR000048 (IQ motif%2C EF-hand binding site)%2C IPR025064 (Domain of unknown function DUF4005)%2C IPR027417 (P-loop containing nucleoside triphosphate hydrolase)%3B GO:0005515 (protein binding)
Araip.B02	5884382	G	G	19	0.00	A	15	0.93	-0.93	0.53	-0.47	0.67	-0.67	upstream_gene_variant	MODIFIER	c.-394T>C	Araip.U3EQS	protein IQ-DOMAIN 1-like isoform X3 [Glycine max]%3B IPR000048 (IQ motif%2C EF-hand binding site)%2C IPR025064 (Domain of unknown function DUF4005)%2C IPR027417 (P-loop containing nucleoside triphosphate hydrolase)%3B GO:0005515 (protein binding)
Araip.B02	5885390	G	G	25	0.00	T	26	0.76	-0.76	0.44	-0.44	0.56	-0.56	upstream_gene_variant	MODIFIER	c.-1402C>A	Araip.U3EQS	protein IQ-DOMAIN 1-like isoform X3 [Glycine max]%3B IPR000048 (IQ motif%2C EF-hand binding site)%2C IPR025064 (Domain of unknown function DUF4005)%2C IPR027417 (P-loop containing nucleoside triphosphate hydrolase)%3B GO:0005515 (protein binding)
Araip.B02	5889813	C	C	19	0.05	G	17	1.00	-0.95	0.47	-0.47	0.65	-0.65	upstream_gene_variant	MODIFIER	c.-4358C>G	Araip.DDJ8Y	S-adenosyl-L-methionine-dependent methyltransferases superfamily protein n%3D1 Tax%3DTheobroma cacao RepID%3DUPI00042B6183
Araip.B02	5902378	G	G	23	0.13	T	21	0.76	-0.63	0.48	-0.48	0.62	-0.62	intergenic_region	MODIFIER	n.5902378T>G	Araip.DDJ8Y-Araip.X635E	
Araip.B02	5905491	G	G	14	0.00	A	19	0.84	-0.84	0.50	-0.50	0.64	-0.64	intergenic_region	MODIFIER	n.5905491G>A	Araip.DDJ8Y-Araip.X635E	
Araip.B02	5960763	G	G	21	0.10	A	12	0.91	-0.81	0.50	-0.50	0.67	-0.67	upstream_gene_variant	MODIFIER	c.-4744C>T	Araip.CC7W1	NAC domain containing protein 12%3B IPR003441 (NAC domain)%3B GO:0003677 (DNA binding)
Araip.B02	5962753	G	G	23	0.00	A	16	0.93	-0.93	0.50	-0.50	0.63	-0.63	intergenic_region	MODIFIER	n.5962753G>A	Araip.CC7W1-Araip.HEW90	
Araip.B02	6010231	T	T	17	0.00	C	14	0.92	-0.92	0.50	-0.50	0.64	-0.64	intergenic_region	MODIFIER	n.6010231C>T	Araip.HEW90-Araip.U5JM0	
Araip.B02	6015164	G	G	25	0.04	A	17	0.94	-0.90	0.47	-0.47	0.65	-0.65	intergenic_region	MODIFIER	n.6015164G>A	Araip.HEW90-Araip.U5JM0	
Araip.B02	6016132	C	C	14	0.07	T	23	0.82	-0.75	0.50	-0.50	0.64	-0.64	intergenic_region	MODIFIER	n.6016132C>T	Araip.HEW90-Araip.U5JM0	
Araip.B02	6022898	G	G	15	0.07	A	21	0.76	-0.69	0.53	-0.47	0.67	-0.67	intergenic_region	MODIFIER	n.6022898G>A	Araip.HEW90-Araip.U5JM0	

Araip.B02	6027583	C	C	18	0.11	T	18	0.83	-0.72	0.50	-0.50	0.61	-0.61	intergenic_region	MODIFIER	n.6027583C>T		Araip.HEW90-Araip.U5JMO	
Araip.B02	6033950	G	G	17	0.00	A	12	0.91	-0.91	0.50	-0.50	0.67	-0.67	intergenic_region	MODIFIER	n.6033950G>A		Araip.HEW90-Araip.U5JMO	
Araip.B02	6066338	C	C	19	0.00	T	28	0.78	-0.78	0.47	-0.47	0.63	-0.63	upstream_gene_variant	MODIFIER	c.-1130G>A		Araip.G92JZ	hypothetical protein
Araip.B02	6081543	G	G	22	0.05	A	20	1.00	-0.95	0.45	-0.45	0.60	-0.60	upstream_gene_variant	MODIFIER	c.-3188C>T		Araip.S3IMP	protein n%3D1 Tax%3DOryza sativa subsp. japonica RepID%3DQ0DGM8_ORYSJ
Araip.B02	6121202	A	A	30	0.03	G	19	1.00	-0.97	0.47	-0.47	0.63	-0.63	missense_variant	MODERATE	c.320C>T	p.Thr107Ile	Araip.R30RS	serine/threonine-protein phosphatase 7 long form homolog [Glycine max]%3B IPR019557 (Aminotransferase-like%2C plant mobile domain)
Araip.B02	6169704	G	G	23	0.17	T	22	0.77	-0.60	0.45	-0.45	0.59	-0.59	upstream_gene_variant	MODIFIER	c.-3721G>T		Araip.3Z628	Vacuolar protein-sorting protein bro1 n%3D4 Tax%3DAspergillaceae RepID%3DBRO1_ASPFU%3B IPR004328 (BRO1 domain)
Araip.B02	6190652	G	G	17	0.00	C	19	0.78	-0.78	0.47	-0.47	0.65	-0.65	downstream_gene_variant	MODIFIER	c.*4484C>G		Araip.08M0G	response regulator 12%3B IPR010402 (CCT domain)%2C IPR011006 (CheY-like superfamily)%3B GO:0000156 (phosphorelay response regulator activity)%2C GO:0000160 (phosphorelay signal transduction system)%2C GO:0005515 (protein binding)
Araip.B02	6197397	A	A	20	0.10	G	20	0.85	-0.75	0.45	-0.45	0.60	-0.60	intergenic_region	MODIFIER	n.6197397G>A		Araip.0JQ81-Araip.TL9EX	
Araip.B02	6199467	C	C	22	0.00	A	24	0.79	-0.79	0.45	-0.45	0.59	-0.59	intergenic_region	MODIFIER	n.6199467C>A		Araip.0JQ81-Araip.TL9EX	
Araip.B02	6206543	G	G	18	0.06	A	27	0.85	-0.79	0.50	-0.50	0.61	-0.61	intergenic_region	MODIFIER	n.6206543G>A		Araip.0JQ81-Araip.TL9EX	
Araip.B02	6222423	G	G	16	0.00	A	15	0.93	-0.93	0.53	-0.47	0.67	-0.67	upstream_gene_variant	MODIFIER	c.-4927G>A		Araip.TL9EX	Zinc ion transmembrane transporter n%3D2 Tax%3DMedicago RepID%3DC9WEK2_MEDSA%3B IPR002524 (Cation efflux protein)%2C IPR027469 (Cation efflux protein transmembrane domain)%3B GO:0006812 (cation transport)%2C GO:0008324 (cation transmembrane transporter activity)%2C GO:0016021 (integral component of membrane)%2C GO:0055085 (transmembrane transport)
Araip.B02	6231733	G	G	17	0.00	A	24	0.83	-0.83	0.47	-0.47	0.65	-0.65	downstream_gene_variant	MODIFIER	c.*3097G>A		Araip.TL9EX	Zinc ion transmembrane transporter n%3D2 Tax%3DMedicago RepID%3DC9WEK2_MEDSA%3B IPR002524 (Cation efflux protein)%2C IPR027469 (Cation efflux protein transmembrane domain)%3B GO:0006812 (cation transport)%2C GO:0008324 (cation transmembrane transporter activity)%2C GO:0016021 (integral component of membrane)%2C GO:0055085 (transmembrane transport)
Araip.B02	6234681	C	C	21	0.24	T	23	0.86	-0.62	0.48	-0.48	0.62	-0.62	intergenic_region	MODIFIER	n.6234681C>T		Araip.TL9EX-Araip.9H3WY	
Araip.B02	6239040	G	G	22	0.00	T	18	0.77	-0.77	0.50	-0.50	0.61	-0.61	intergenic_region	MODIFIER	n.6239040G>T		Araip.TL9EX-Araip.9H3WY	
Araip.B02	6246329	C	C	14	0.07	A	16	0.81	-0.74	0.50	-0.50	0.64	-0.64	intergenic_region	MODIFIER	n.6246329C>A		Araip.TL9EX-Araip.9H3WY	

Araip.B02	6256880	G	G	28	0.00	A	10	1.00	-1.00	0.50	-0.60	0.70	-0.70	downstream_gene_variant	MODIFIER	c.*1101G>A		Araip.9H3WY	basic helix-loop-helix (bHLH) DNA-binding superfamily protein%3B IPR011598 (Myc-type%2C basic helix-loop-helix (bHLH) domain)%3B GO:0046983 (protein dimerization activity)
Araip.B02	6261054	C	C	12	0.00	T	14	0.71	-0.71	0.50	-0.50	0.67	-0.67	intergenic_region	MODIFIER	n.6261054C>T		Araip.9H3WY-Araip.TJ2RV	
Araip.B02	6273162	T	T	22	0.09	C	17	0.82	-0.73	0.47	-0.47	0.65	-0.65	intergenic_region	MODIFIER	n.6273162C>T		Araip.9H3WY-Araip.TJ2RV	
Araip.B02	6273949	C	C	12	0.08	T	13	0.92	-0.84	0.50	-0.50	0.67	-0.67	intergenic_region	MODIFIER	n.6273949C>T		Araip.9H3WY-Araip.TJ2RV	
Araip.B02	6282412	C	C	20	0.15	T	22	0.81	-0.66	0.45	-0.45	0.60	-0.60	upstream_gene_variant	MODIFIER	c.-222C>T		Araip.TJ2RV	receptor-like kinase%3B IPR001611 (Leucine-rich repeat)%2C IPR013210 (Leucine-rich repeat-containing N-terminal%2C type 2)%3B GO:0005515 (protein binding)
Araip.B02	6287545	T	T	15	0.00	C	23	0.82	-0.82	0.53	-0.47	0.67	-0.67	downstream_gene_variant	MODIFIER	c.*3276C>T		Araip.TJ2RV	receptor-like kinase%3B IPR001611 (Leucine-rich repeat)%2C IPR013210 (Leucine-rich repeat-containing N-terminal%2C type 2)%3B GO:0005515 (protein binding)
Araip.B02	6292722	C	C	18	0.06	G	21	0.76	-0.70	0.50	-0.50	0.61	-0.61	intergenic_region	MODIFIER	n.6292722C>G		Araip.C5BJ8-Araip.4C72L	
Araip.B02	6305789	G	G	10	0.00	T	13	1.00	-1.00	0.50	-0.60	0.70	-0.70	downstream_gene_variant	MODIFIER	c.*4551C>A		Araip.4C72L	stress up-regulated Nod 19 protein%3B IPR011692 (Stress up-regulated Nod 19)
Araip.B02	6309002	C	C	25	0.12	A	18	0.83	-0.71	0.50	-0.50	0.61	-0.61	downstream_gene_variant	MODIFIER	c.*1338G>T		Araip.4C72L	stress up-regulated Nod 19 protein%3B IPR011692 (Stress up-regulated Nod 19)
Araip.B02	6329882	T	T	19	0.16	C	23	0.86	-0.70	0.47	-0.47	0.63	-0.63	intron_variant	MODIFIER	c.1320+869G>A		Araip.NXC1U	stress up-regulated Nod 19 protein%3B IPR011692 (Stress up-regulated Nod 19)
Araip.B02	6334746	T	T	15	0.00	A	19	0.68	-0.68	0.53	-0.47	0.67	-0.67	synonymous_variant	LOW	c.849T>A	p.Ile283Ile	Araip.NXC1U	stress up-regulated Nod 19 protein%3B IPR011692 (Stress up-regulated Nod 19)
Araip.B02	6343425	G	G	21	0.00	A	18	0.94	-0.94	0.50	-0.50	0.61	-0.61	downstream_gene_variant	MODIFIER	c.*4327G>A		Araip.SX1UB	thylakoid membrane phosphoprotein 14 kDa protein%3B IPR025564 (Cyanobacterial aminoacyl-tRNA synthetase%2C CAAD domain)
Araip.B02	6356116	T	T	15	0.13	C	20	0.85	-0.72	0.53	-0.47	0.67	-0.67	intergenic_region	MODIFIER	n.6356116T>C		Araip.SX3RK-Araip.VA01D	
Araip.B02	6357112	A	A	14	0.14	C	12	1.00	-0.86	0.50	-0.50	0.67	-0.67	intergenic_region	MODIFIER	n.6357112C>A		Araip.SX3RK-Araip.VA01D	
Araip.B02	6361449	A	A	17	0.00	G	27	0.77	-0.77	0.47	-0.47	0.65	-0.65	downstream_gene_variant	MODIFIER	c.*767T>C		Araip.VA01D	Unknown protein
Araip.B02	6364567	T	T	14	0.07	C	24	0.83	-0.76	0.50	-0.50	0.64	-0.64	upstream_gene_variant	MODIFIER	c.-2010G>A		Araip.VA01D	Unknown protein
Araip.B02	6375003	G	G	17	0.00	A	19	0.68	-0.68	0.47	-0.47	0.65	-0.65	intron_variant	MODIFIER	c.1230+91C>T		Araip.E3E4E	O-methyltransferase 1%3B IPR001077 (O-methyltransferase%2C family 2)%2C IPR012967 (Plant methyltransferase dimerisation)%3B GO:0008171 (O-methyltransferase activity)%2C GO:0046983 (protein dimerization activity)

Araip.B02	6395627	T	T	17	0.00	C	17	0.94	-0.94	0.47	-0.47	0.65	-0.65	downstream_gene_variant	MODIFIER	c.*436G>A		Araip.I128H	2-oxoglutarate (2OG) and Fe(II)-dependent oxygenase superfamily protein%3B IPR002283 (Isopenicillin N synthase)%2C IPR026992 (Non-haem dioxygenase N-terminal domain)%2C IPR027443 (Isopenicillin N synthase-like)%3B GO:0005506 (iron ion binding)%2C GO:0016491 (oxidoreductase activity)%2C GO:0055114 (oxidation-reduction process)
Araip.B02	6410636	T	T	30	0.07	C	48	0.64	-0.57	0.43	-0.43	0.57	-0.57	intergenic_region	MODIFIER	n.6410636C>T		Araip.I128H-Araip.4GOH6	
Araip.B02	6446982	T	T	20	0.00	C	16	0.81	-0.81	0.50	-0.50	0.63	-0.63	upstream_gene_variant	MODIFIER	c.-1084G>A		Araip.4GOH6	DNA ligase I%2C ATP-dependent protein%3B IPR001810 (F-box domain)%2C IPR011043 (Galactose oxidase/kelch%2C beta-propeller)%2C IPR012308 (DNA ligase%2C ATP-dependent%2C N-terminal)%2C IPR017451 (F-box associated interaction domain)%3B GO:0003677 (DNA binding)%2C GO:0003910 (DNA ligase (ATP) activity)%2C GO:0005515 (protein binding)%2C GO:0006281 (DNA repair)%2C GO:0006310 (DNA recombination)
Araip.B02	6470236	C	C	22	0.09	G	26	0.76	-0.67	0.45	-0.45	0.59	-0.59	missense_variant	MODERATE	c.335C>G	p.Ala112Gly	Araip.VZ4DX	Ulp1 protease family%2C carboxy-terminal domain protein%3B IPR003653 (Peptidase C48%2C SUMO/Sentrin/Ubl1)%3B GO:0006508 (proteolysis)%2C GO:0008234 (cysteine-type peptidase activity)
Araip.B02	6471046	T	T	21	0.00	C	25	0.84	-0.84	0.48	-0.48	0.62	-0.62	missense_variant	MODERATE	c.707C>T	p.Ser236Phe	Araip.VZ4DX	Ulp1 protease family%2C carboxy-terminal domain protein%3B IPR003653 (Peptidase C48%2C SUMO/Sentrin/Ubl1)%3B GO:0006508 (proteolysis)%2C GO:0008234 (cysteine-type peptidase activity)
Araip.B02	6490299	A	A	18	0.00	G	16	0.81	-0.81	0.50	-0.50	0.63	-0.63	intergenic_region	MODIFIER	n.6490299G>A		Araip.17KGH-Araip.04KBR	
Araip.B02	6530047	T	T	29	0.00	C	25	0.76	-0.76	0.44	-0.44	0.56	-0.56	intergenic_region	MODIFIER	n.6530047T>C		Araip.POTWG-Araip.BFF3A	
Araip.B02	6531032	G	G	20	0.10	A	24	0.83	-0.73	0.45	-0.45	0.60	-0.60	intergenic_region	MODIFIER	n.6531032G>A		Araip.POTWG-Araip.BFF3A	
Araip.B02	6537097	G	G	11	0.09	A	14	0.92	-0.83	0.55	-0.55	0.64	-0.73	intergenic_region	MODIFIER	n.6537097G>A		Araip.POTWG-Araip.BFF3A	
Araip.B02	6548388	G	G	22	0.00	A	20	0.80	-0.80	0.45	-0.45	0.60	-0.60	intergenic_region	MODIFIER	n.6548388A>G		Araip.POTWG-Araip.BFF3A	
Araip.B02	6552305	T	T	14	0.00	C	25	1.00	-1.00	0.50	-0.50	0.64	-0.64	intergenic_region	MODIFIER	n.6552305T>C		Araip.POTWG-Araip.BFF3A	
Araip.B02	6555214	T	T	18	0.00	C	24	0.95	-0.95	0.50	-0.50	0.61	-0.61	intergenic_region	MODIFIER	n.6555214C>T		Araip.POTWG-Araip.BFF3A	
Araip.B02	6555244	C	C	20	0.00	T	26	0.96	-0.96	0.45	-0.45	0.60	-0.60	intergenic_region	MODIFIER	n.6555244C>T		Araip.POTWG-Araip.BFF3A	
Araip.B02	6583673	T	T	37	0.03	C	20	0.95	-0.92	0.45	-0.45	0.60	-0.60	intergenic_region	MODIFIER	n.6583673C>T		Araip.BFF3A-Araip.LIL9G	
Araip.B02	6587027	G	G	18	0.16	A	16	0.88	-0.72	0.50	-0.50	0.63	-0.63	intergenic_region	MODIFIER	n.6587027G>A		Araip.BFF3A-Araip.LIL9G	
Araip.B02	6589765	G	G	14	0.00	A	21	0.80	-0.80	0.50	-0.50	0.64	-0.64	intergenic_region	MODIFIER	n.6589765G>A		Araip.BFF3A-Araip.LIL9G	
Araip.B02	6609168	A	A	32	0.00	T	23	0.86	-0.86	0.43	-0.43	0.57	-0.61	downstream_gene_variant	MODIFIER	c.*496T>A		Araip.LIL9G	Arv1-like protein%3B IPR007290 (Arv1 protein)

Araip.B02	6614497	G	G	13	0.08	A	32	0.96	-0.88	0.54	-0.54	0.62	-0.69	upstream_gene_variant	MODIFIER	c.-755C>T	Araip.MQD5S	NAC domain containing protein 62%3B IPR003441 (NAC domain)%3B GO:0003677 (DNA binding)
Araip.B02	6616839	G	G	13	0.00	A	25	0.92	-0.92	0.54	-0.54	0.62	-0.69	upstream_gene_variant	MODIFIER	c.-3097C>T	Araip.MQD5S	NAC domain containing protein 62%3B IPR003441 (NAC domain)%3B GO:0003677 (DNA binding)
Araip.B02	6622970	G	G	11	0.00	A	25	0.76	-0.76	0.55	-0.55	0.64	-0.73	intergenic_region	MODIFIER	n.6622970G>A	Araip.MQD5S-Araip.A6QWC	
Araip.B02	6655945	T	T	11	0.00	C	26	0.80	-0.80	0.55	-0.55	0.64	-0.73	upstream_gene_variant	MODIFIER	c.-1822G>A	Araip.A6QWC	NAC domain containing protein 62%3B IPR003441 (NAC domain)%3B GO:0003677 (DNA binding)
Araip.B02	6671701	G	G	17	0.00	T	16	0.87	-0.87	0.50	-0.50	0.63	-0.63	intergenic_region	MODIFIER	n.6671701G>T	Araip.A6QWC-Araip.64IP1	
Araip.B02	6680512	G	G	10	0.00	A	25	0.92	-0.92	0.50	-0.60	0.70	-0.70	intergenic_region	MODIFIER	n.6680512G>A	Araip.A6QWC-Araip.64IP1	
Araip.B02	6683030	T	T	21	0.10	C	17	0.82	-0.72	0.47	-0.47	0.65	-0.65	downstream_gene_variant	MODIFIER	c.*2965G>A	Araip.64IP1	Calcium-binding endonuclease/exonuclease/phosphatase family%3B IPR005135 (Endonuclease/exonuclease/phosphatase)%2C IPR011992 (EF-hand domain pair)%3B GO:0005509 (calcium ion binding)
Araip.B02	6693008	A	A	16	0.00	G	19	0.94	-0.94	0.50	-0.50	0.63	-0.63	upstream_gene_variant	MODIFIER	c.-3181T>C	Araip.64IP1	Calcium-binding endonuclease/exonuclease/phosphatase family%3B IPR005135 (Endonuclease/exonuclease/phosphatase)%2C IPR011992 (EF-hand domain pair)%3B GO:0005509 (calcium ion binding)
Araip.B02	6699651	C	C	25	0.08	A	18	0.72	-0.64	0.50	-0.50	0.61	-0.61	upstream_gene_variant	MODIFIER	c.-1406G>T	Araip.6ZN4Z	ribosome production factor-like protein%3B IPR004154 (Anticodon-binding)%2C IPR007109 (Brix domain)
Araip.B02	6702583	G	G	26	0.00	T	20	0.85	-0.85	0.45	-0.45	0.60	-0.60	upstream_gene_variant	MODIFIER	c.-4338C>A	Araip.6ZN4Z	ribosome production factor-like protein%3B IPR004154 (Anticodon-binding)%2C IPR007109 (Brix domain)
Araip.B02	6717053	C	C	18	0.11	T	22	0.95	-0.84	0.50	-0.50	0.61	-0.61	upstream_gene_variant	MODIFIER	c.-3468G>A	Araip.XVM4V	purple acid phosphatase 29%3B IPR011230 (Phosphoesterase At2g46880)%3B GO:0016787 (hydrolase activity)
Araip.B02	6718176	T	T	23	0.00	C	28	0.78	-0.78	0.43	-0.43	0.57	-0.61	upstream_gene_variant	MODIFIER	c.-4591G>A	Araip.XVM4V	purple acid phosphatase 29%3B IPR011230 (Phosphoesterase At2g46880)%3B GO:0016787 (hydrolase activity)
Araip.B02	6734782	G	G	26	0.12	T	29	0.82	-0.70	0.42	-0.46	0.54	-0.58	intergenic_region	MODIFIER	n.6734782G>T	Araip.WFR11-Araip.PEM8B	
Araip.B02	6742382	C	C	18	0.22	T	12	1.00	-0.78	0.50	-0.50	0.67	-0.67	intergenic_region	MODIFIER	n.6742382C>T	Araip.WFR11-Araip.PEM8B	
Araip.B02	6744797	C	C	18	0.00	T	15	0.73	-0.73	0.53	-0.47	0.67	-0.67	downstream_gene_variant	MODIFIER	c.*5045G>A	Araip.PEM8B	Protein of unknown function (DUF1295)%3B IPR010721 (Protein of unknown function DUF1295)
Araip.B02	6760101	T	T	17	0.12	C	21	0.90	-0.78	0.47	-0.47	0.65	-0.65	upstream_gene_variant	MODIFIER	c.-2330A>G	Araip.T5WNM	transcription factor 25-like [Glycine max]%3B IPR006994 (Transcription factor 25)
Araip.B02	6771633	C	C	23	0.09	T	18	0.94	-0.85	0.50	-0.50	0.61	-0.61	downstream_gene_variant	MODIFIER	c.*4639C>T	Araip.3A95K	Unknown protein
Araip.B02	6773423	G	G	12	0.00	A	24	0.83	-0.83	0.50	-0.50	0.67	-0.67	downstream_gene_variant	MODIFIER	c.*2896G>A	Araip.YO3J3	hypothetical protein
Araip.B02	6776001	C	C	31	0.03	T	18	1.00	-0.97	0.50	-0.50	0.61	-0.61	5_prime_UTR_variant	MODIFIER	c.-1559A>G	Araip.X1ALQ	Unknown protein
Araip.B02	6807794	T	T	18	0.11	C	12	1.00	-0.89	0.50	-0.50	0.67	-0.67	intergenic_region	MODIFIER	n.6807794C>T	Araip.I9VQZ-Araip.866IZ	
Araip.B02	6808732	G	G	12	0.00	A	14	0.92	-0.92	0.50	-0.50	0.67	-0.67	intergenic_region	MODIFIER	n.6808732G>A	Araip.I9VQZ-Araip.866IZ	

Araip.B02	6810305	C	C	23	0.17	T	20	1.00	-0.83	0.45	-0.45	0.60	-0.60	intergenic_region	MODIFIER	n.6810305C>T	Araip.I9VQZ-Araip.866IZ	
Araip.B02	6816826	C	C	16	0.00	T	12	0.75	-0.75	0.50	-0.50	0.67	-0.67	intergenic_region	MODIFIER	n.6816826C>T	Araip.I9VQZ-Araip.866IZ	
Araip.B02	6817378	C	C	10	0.00	T	13	1.00	-1.00	0.50	-0.60	0.70	-0.70	intergenic_region	MODIFIER	n.6817378C>T	Araip.I9VQZ-Araip.866IZ	
Araip.B02	6852677	G	G	16	0.12	A	25	0.92	-0.80	0.50	-0.50	0.63	-0.63	intergenic_region	MODIFIER	n.6852677G>A	Araip.XD6TC-Araip.I5XHJ	
Araip.B02	6854733	C	C	11	0.00	T	16	0.75	-0.75	0.55	-0.55	0.64	-0.73	intergenic_region	MODIFIER	n.6854733T>C	Araip.XD6TC-Araip.I5XHJ	
Araip.B02	6876093	C	C	17	0.06	T	11	1.00	-0.94	0.55	-0.55	0.64	-0.73	upstream_gene_variant	MODIFIER	c.-3458C>T	Araip.V7FAZ	Protein kinase superfamily protein%3B IPR011009 (Protein kinase-like domain)%2C IPR013320 (Concanavalin A-like lectin/glucanase%2C subgroup)%3B GO:0004672 (protein kinase activity)%2C GO:0005524 (ATP binding)%2C GO:0006468 (protein phosphorylation)
Araip.B02	6903908	G	G	25	0.08	T	26	0.92	-0.84	0.44	-0.44	0.56	-0.56	intergenic_region	MODIFIER	n.6903908G>T	Araip.Y2TM4-Araip.26RHR	
Araip.B02	6916395	G	G	27	0.00	A	18	0.83	-0.83	0.50	-0.50	0.61	-0.61	upstream_gene_variant	MODIFIER	c.-1333C>T	Araip.26RHR	unknown protein%3B FUNCTIONS IN: molecular_function unknown%3B INVOLVED IN: biological_process unknown%3B LOCATED IN: mitochondrion%2C plastid%3B EXPRESSED IN: 23 plant structures%3B EXPRESSED DURING: 13 growth stages %3B IPR018786 (Protein of unknown function DUF2343)
Araip.B02	6931414	A	A	19	0.00	G	23	0.69	-0.69	0.47	-0.47	0.63	-0.63	intron_variant	MODIFIER	c.*274-1231C>T	Araip.7KX61	unknown protein
Araip.B02	6938207	A	A	24	0.00	G	16	0.81	-0.81	0.50	-0.50	0.63	-0.63	upstream_gene_variant	MODIFIER	c.-224C>T	Araip.7KX61	unknown protein
Araip.B02	6949316	G	G	16	0.06	A	25	0.72	-0.66	0.50	-0.50	0.63	-0.63	intron_variant	MODIFIER	c.*101-90C>T	Araip.SBL82	unknown protein%3B FUNCTIONS IN: molecular_function unknown%3B INVOLVED IN: biological_process unknown%3B LOCATED IN: mitochondrion%2C plastid%3B EXPRESSED IN: 23 plant structures%3B EXPRESSED DURING: 13 growth stages %3B IPR018786 (Protein of unknown function DUF2343)
