# Supplementary material for: Discovery of genomic regions and candidate genes controlling shelling percentage using QTL‐seq approach in cultivated peanut (Arachis hypogaea L.)
Source: Plant Biotechnol J. 2019 Jan 30;17(7):1248–60. doi: 10.1111/pbi.13050 (PMC6576108; doi:10.1111/pbi.13050)
Supplement: Supplementary file 18 — Table S6 Genotypes of the developed KASP markers in the RIL population. [file PBI-17-1248-s014.pdf]

Table S6 Genotypes of the developed KASP markers in the RIL population.

RILs	Aradu_A09_66949737	Araip_B02_6155951	Araip_B02_6770282	Araip_B02_6776001
QT487	G	T	A	T
QT488	-	G	G	C
QT489	-	T	A	T
QT490	G	T	?	T
QT491	-	G	G	C
QT492	A	G	G	C
QT493	A	G	G	C
QT494	A	T	G	C
QT495	A	T	A	T
QT496	-	?	A	T
QT497	-	T	A	T
QT498	A	T	A	T
QT499	A	G	G	C
QT500	-	T	A	T
QT501	-	T	A	T
QT502	-	G	G	C
QT503	G	G	G	C
QT504	-	G	G	C
QT505	H	-	-	-
QT506	A	T	?	T
QT507	G	T	A	T
QT508	?	H	H	H
QT509	A	T	A	T
QT510	A	T	A	T
QT511	A	T	A	?
QT512	A	T	A	T
QT513	-	T	A	T
QT514	G	T	A	T
QT515	G	-	-	-
QT516	G	G	H	H
QT517	A	T	A	T
QT518	A	T	H	H
QT519	A	T	A	T
QT520	A	G	G	C
QT521	A	G	G	C
QT522	-	T	A	T
QT523	G	G	G	C
QT524	A	T	A	T
QT525	A	T	A	T
QT526	H	G	G	C
QT527	A	G	G	C
QT528	G	-	-	-
QT529	G	G	G	?
QT530	G	G	G	?
QT531	G	T	A	T
QT532	A	T	A	T
QT533	A	G	G	C
QT534	A	G	G	C
QT535	G	G	G	C
QT536	G	T	A	T
QT537	G	T	A	T
QT538	G	G	G	C
QT539	A	?	G	C
QT540	A	G	G	?
QT541	A	T	A	T
QT542	A	T	A	T
QT543	A	T	A	T
QT544	G	-	-	-
QT545	G	G	G	C
QT546	A	G	G	C

QT547	A	G	G	C
QT548	G	G	G	C
QT549	G	G	G	C
QT550	G	G	G	C
QT551	G	G	G	C
QT552	G	G	G	C
QT553	G	G	?	?
QT554	A	T	A	T
QT555	A	T	A	T
QT556	G	G	G	C
QT557	G	?	G	C
QT558	G	G	G	C
QT559	A	-	-	-
QT560	A	G	G	C
QT561	G	T	A	T
QT562	G	T	A	T
QT563	G	G	G	C
QT564	G	?	G	C
QT565	A	T	A	T
QT566	A	T	A	T
QT567	A	T	A	T
QT568	A	T	A	T
QT569	G	G	G	C
QT570	A	G	G	C
QT571	A	T	A	T
QT572	G	G	G	C
QT573	A	G	G	C
QT574	A	G	G	C
QT575	A	T	A	T
QT576	A	G	G	C
QT577	A	G	G	C
QT578	G	G	G	C
QT579	A	T	A	T
QT580	A	T	A	T
QT581	A	T	G	C
QT582	G	T	G	C
QT583	G	T	A	T
QT584	G	T	A	T
QT585	A	G	G	C
QT586	G	-	-	-
QT587	G	G	A	T
QT588	G	T	A	T
QT589	G	T	A	T
QT590	G	G	?	C
QT591	G	T	A	T
QT592	A	G	?	C
QT593	A	T	A	T
QT594	G	G	G	C
QT595	G	G	G	C
QT596	A	T	A	T
QT597	G	G	G	C
QT598	G	G	G	C
QT599	A	T	A	T
QT600	A	G	G	C
QT601	G	G	G	C
QT602	G	G	G	C
QT603	G	G	G	C
QT604	G	T	A	T
QT605	G	T	A	T
QT606	A	T	A	T
QT607	G	G	?	C
QT608	G	T	A	T
QT609	A	T	A	T
QT610	G	G	G	C

QT611	A	T	A	?
QT612	G	G	G	C
QT613	A	?	G	C
QT614	A	T	A	?
QT615	A	G	G	C
QT616	A	G	G	?
QT617	A	T	A	T
QT618	A	T	?	T
QT619	A	G	?	C
QT620	A	-	-	-
QT621	G	T	?	T
QT622	G	G	G	C
QT623	A	G	G	C
QT624	A	G	G	C
QT625	G	T	A	T
QT626	-	G	G	C
QT627	G	H	H	H
QT628	G	G	A	T
QT629	A	T	A	T
QT630	G	G	G	C
QT631	G	G	G	C
QT632	A	T	A	?
QT633	G	T	A	T
QT634	A	G	G	C
QT635	G	G	G	C
QT636	G	G	G	C
QT637	G	G	G	C
QT638	G	G	A	T
QT639	G	T	A	?
QT640	A	T	A	T
QT641	G	-	-	-
QT642	G	G	G	?
QT643	G	G	G	C
QT644	G	G	G	C
QT645	G	?	A	T
QT646	A	G	G	C
QT647	A	T	A	T
QT648	G	G	G	C
QT649	G	G	G	C
QT650	G	T	A	T
QT651	G	G	G	C
QT652	G	T	A	T
QT653	G	G	G	C
QT654	G	T	A	T
QT655	G	G	A	T
QT656	G	G	G	C
QT657	A	?	A	T
QT658	G	T	A	T
QT659	G	G	G	C
QT660	G	G	G	C
QT661	A	-	-	-
QT662	G	G	G	C
QT663	G	G	G	C
QT664	A	G	G	C
QT665	A	G	G	C
QT666	G	G	G	C
QT667	A	T	A	T
QT668	A	T	A	T
QT669	G	G	G	C
QT670	G	G	G	C
QT671	G	G	G	C
QT672	-	G	G	C
QT673	G	T	A	T
QT674	A	G	G	C

QT675	G	G	G	C
QT676	A	T	A	T
QT677	G	G	G	C
QT678	A	T	A	T
QT679	G	T	A	?
QT1980	-	G	G	C
QT1981	-	H	A	T
Xuzhou68-4	G	G	G	C
Yuanza 9102	A	T	A	T
