# Supplementary material for: Discovery of genomic regions and candidate genes controlling shelling percentage using QTL‐seq approach in cultivated peanut (Arachis hypogaea L.)
Source: Plant Biotechnol J. 2019 Jan 30;17(7):1248–60. doi: 10.1111/pbi.13050 (PMC6576108; doi:10.1111/pbi.13050)
Supplement: Supplementary file 19 — Table S7 Phenotyping data generated in the Yangluo2017 environment for the RIL population. [file PBI-17-1248-s015.pdf]

Table S7 Phenotyping data generated in the Yangluo2017 environment for the RIL population.

Environment	Block	RILs	Shelling percentage(%)
Yangluo2017	1	QT487	76.22
Yangluo2017	1	QT488	71.83
Yangluo2017	1	QT489	75.47
Yangluo2017	1	QT490	73.20
Yangluo2017	1	QT491	73.44
Yangluo2017	1	QT492	77.41
Yangluo2017	1	QT493	79.54
Yangluo2017	1	QT494	75.08
Yangluo2017	1	QT495	80.99
Yangluo2017	1	QT496	77.48
Yangluo2017	1	QT497	78.73
Yangluo2017	1	QT498	77.98
Yangluo2017	1	QT499	72.12
Yangluo2017	1	QT500	69.29
Yangluo2017	1	QT501	79.50
Yangluo2017	1	QT502	72.51
Yangluo2017	1	QT503	72.41
Yangluo2017	1	QT504	73.45
Yangluo2017	1	QT505	70.44
Yangluo2017	1	QT506	77.41
Yangluo2017	1	QT507	75.96
Yangluo2017	1	QT508	74.53
Yangluo2017	1	QT509	72.10
Yangluo2017	1	QT510	79.58
Yangluo2017	1	QT511	81.24
Yangluo2017	1	QT512	79.86
Yangluo2017	1	QT513	73.94
Yangluo2017	1	QT514	81.36
Yangluo2017	1	QT515	73.60
Yangluo2017	1	QT516	74.59
Yangluo2017	1	QT517	77.38
Yangluo2017	1	QT518	79.87
Yangluo2017	1	QT519	69.36
Yangluo2017	1	QT520	72.33
Yangluo2017	1	QT521	74.86
Yangluo2017	1	QT522	77.71
Yangluo2017	1	QT523	65.35
Yangluo2017	1	QT524	78.38
Yangluo2017	1	QT525	76.59
Yangluo2017	1	QT526	74.40

Yangluo2017	1	QT527	72.98
Yangluo2017	1	QT528	67.59
Yangluo2017	1	QT529	81.45
Yangluo2017	1	QT530	77.27
Yangluo2017	1	QT531	73.22
Yangluo2017	1	QT532	75.71
Yangluo2017	1	QT533	76.24
Yangluo2017	1	QT534	76.92
Yangluo2017	1	QT535	65.93
Yangluo2017	1	QT536	72.75
Yangluo2017	1	QT537	74.26
Yangluo2017	1	QT538	69.83
Yangluo2017	1	QT539	71.00
Yangluo2017	1	QT540	79.37
Yangluo2017	1	QT541	78.57
Yangluo2017	1	QT542	76.26
Yangluo2017	1	QT543	-
Yangluo2017	1	QT544	68.27
Yangluo2017	1	QT545	78.13
Yangluo2017	1	QT546	72.13
Yangluo2017	1	QT547	79.58
Yangluo2017	1	QT548	67.78
Yangluo2017	1	QT549	67.43
Yangluo2017	1	QT550	68.92
Yangluo2017	1	QT551	76.84
Yangluo2017	1	QT552	71.50
Yangluo2017	1	QT553	74.31
Yangluo2017	1	QT554	77.01
Yangluo2017	1	QT555	74.67
Yangluo2017	1	QT556	74.94
Yangluo2017	1	QT557	72.47
Yangluo2017	1	QT558	70.66
Yangluo2017	1	QT559	73.05
Yangluo2017	1	QT560	76.60
Yangluo2017	1	QT561	78.25
Yangluo2017	1	QT562	77.05
Yangluo2017	1	QT563	71.44
Yangluo2017	1	QT564	72.10
Yangluo2017	1	QT565	80.11
Yangluo2017	1	QT566	77.87
Yangluo2017	1	QT567	75.23
Yangluo2017	1	QT568	78.37
Yangluo2017	1	QT569	70.45

Yangluo2017	1	QT570	73.16
Yangluo2017	1	QT571	80.93
Yangluo2017	1	QT572	70.31
Yangluo2017	1	QT573	69.46
Yangluo2017	1	QT574	-
Yangluo2017	1	QT575	77.68
Yangluo2017	1	QT576	72.72
Yangluo2017	1	QT577	70.61
Yangluo2017	1	QT578	73.58
Yangluo2017	1	QT579	77.81
Yangluo2017	1	QT580	80.40
Yangluo2017	1	QT581	75.75
Yangluo2017	1	QT582	76.33
Yangluo2017	1	QT583	78.15
Yangluo2017	1	QT584	78.44
Yangluo2017	1	QT585	77.07
Yangluo2017	1	QT586	74.70
Yangluo2017	1	QT587	70.29
Yangluo2017	1	QT588	80.61
Yangluo2017	1	QT589	80.56
Yangluo2017	1	QT590	75.26
Yangluo2017	1	QT591	70.30
Yangluo2017	1	QT592	72.50
Yangluo2017	1	QT593	76.43
Yangluo2017	1	QT594	74.66
Yangluo2017	1	QT595	74.35
Yangluo2017	1	QT596	74.37
Yangluo2017	1	QT597	69.18
Yangluo2017	1	QT598	-
Yangluo2017	1	QT599	76.85
Yangluo2017	1	QT600	79.65
Yangluo2017	1	QT601	69.66
Yangluo2017	1	QT602	69.18
Yangluo2017	1	QT603	69.89
Yangluo2017	1	QT604	74.23
Yangluo2017	1	QT605	76.64
Yangluo2017	1	QT606	78.23
Yangluo2017	1	QT607	71.16
Yangluo2017	1	QT608	76.87
Yangluo2017	1	QT609	76.83
Yangluo2017	1	QT610	73.24
Yangluo2017	1	QT611	78.45
Yangluo2017	1	QT612	76.61

Yangluo2017	1	QT613	72.28
Yangluo2017	1	QT614	70.53
Yangluo2017	1	QT615	72.58
Yangluo2017	1	QT616	79.58
Yangluo2017	1	QT617	75.11
Yangluo2017	1	QT618	78.00
Yangluo2017	1	QT619	81.61
Yangluo2017	1	QT620	71.67
Yangluo2017	1	QT621	74.47
Yangluo2017	1	QT622	77.69
Yangluo2017	1	QT623	73.34
Yangluo2017	1	QT624	77.94
Yangluo2017	1	QT625	77.54
Yangluo2017	1	QT626	76.10
Yangluo2017	1	QT627	71.58
Yangluo2017	1	QT628	77.09
Yangluo2017	1	QT629	74.03
Yangluo2017	1	QT630	69.32
Yangluo2017	1	QT631	67.24
Yangluo2017	1	QT632	74.13
Yangluo2017	1	QT633	75.40
Yangluo2017	1	QT634	69.88
Yangluo2017	1	QT635	74.33
Yangluo2017	1	QT636	72.27
Yangluo2017	1	QT637	71.82
Yangluo2017	1	QT638	68.92
Yangluo2017	1	QT639	73.74
Yangluo2017	1	QT640	78.99
Yangluo2017	1	QT641	80.08
Yangluo2017	1	QT642	73.31
Yangluo2017	1	QT643	68.86
Yangluo2017	1	QT644	-
Yangluo2017	1	QT645	75.81
Yangluo2017	1	QT646	72.70
Yangluo2017	1	QT647	75.73
Yangluo2017	1	QT648	67.84
Yangluo2017	1	QT649	78.39
Yangluo2017	1	QT650	71.43
Yangluo2017	1	QT651	71.07
Yangluo2017	1	QT652	71.04
Yangluo2017	1	QT653	75.99
Yangluo2017	1	QT654	76.49
Yangluo2017	1	QT655	75.61

Yangluo2017	1	QT656	73.71
Yangluo2017	1	QT657	74.36
Yangluo2017	1	QT658	67.11
Yangluo2017	1	QT659	67.45
Yangluo2017	1	QT660	71.31
Yangluo2017	1	QT661	74.30
Yangluo2017	1	QT662	68.17
Yangluo2017	1	QT663	71.32
Yangluo2017	1	QT664	77.01
Yangluo2017	1	QT665	71.88
Yangluo2017	1	QT666	70.19
Yangluo2017	1	QT667	75.51
Yangluo2017	1	QT668	77.95
Yangluo2017	1	QT669	72.98
Yangluo2017	1	QT670	78.18
Yangluo2017	1	QT671	71.03
Yangluo2017	1	QT672	65.92
Yangluo2017	1	QT673	74.73
Yangluo2017	1	QT674	69.02
Yangluo2017	1	QT675	66.35
Yangluo2017	1	QT676	73.80
Yangluo2017	1	QT677	72.10
Yangluo2017	1	QT678	80.10
Yangluo2017	1	QT679	73.87
Yangluo2017	1	QT1980	70.28
Yangluo2017	1	QT1981	73.92
Yangluo2017	1	Xuzhou68-4	72.15
Yangluo2017	1	Yuanza 9102	78.69
Yangluo2017	2	QT487	77.21
Yangluo2017	2	QT488	71.55
Yangluo2017	2	QT489	75.88
Yangluo2017	2	QT490	72.79
Yangluo2017	2	QT491	72.15
Yangluo2017	2	QT492	77.65
Yangluo2017	2	QT493	80.30
Yangluo2017	2	QT494	74.12
Yangluo2017	2	QT495	81.84
Yangluo2017	2	QT496	76.70
Yangluo2017	2	QT497	79.61
Yangluo2017	2	QT498	77.58
Yangluo2017	2	QT499	73.23
Yangluo2017	2	QT500	69.21
Yangluo2017	2	QT501	77.21

Yangluo2017	2	QT502	72.81
Yangluo2017	2	QT503	73.77
Yangluo2017	2	QT504	72.54
Yangluo2017	2	QT505	69.41
Yangluo2017	2	QT506	77.71
Yangluo2017	2	QT507	77.50
Yangluo2017	2	QT508	75.05
Yangluo2017	2	QT509	71.42
Yangluo2017	2	QT510	79.67
Yangluo2017	2	QT511	79.24
Yangluo2017	2	QT512	81.05
Yangluo2017	2	QT513	72.84
Yangluo2017	2	QT514	79.57
Yangluo2017	2	QT515	72.97
Yangluo2017	2	QT516	73.14
Yangluo2017	2	QT517	76.65
Yangluo2017	2	QT518	78.95
Yangluo2017	2	QT519	69.15
Yangluo2017	2	QT520	72.76
Yangluo2017	2	QT521	74.31
Yangluo2017	2	QT522	77.37
Yangluo2017	2	QT523	66.60
Yangluo2017	2	QT524	78.87
Yangluo2017	2	QT525	78.21
Yangluo2017	2	QT526	75.71
Yangluo2017	2	QT527	72.00
Yangluo2017	2	QT528	67.57
Yangluo2017	2	QT529	82.10
Yangluo2017	2	QT530	77.42
Yangluo2017	2	QT531	72.77
Yangluo2017	2	QT532	74.42
Yangluo2017	2	QT533	75.51
Yangluo2017	2	QT534	76.48
Yangluo2017	2	QT535	67.65
Yangluo2017	2	QT536	71.87
Yangluo2017	2	QT537	75.31
Yangluo2017	2	QT538	69.07
Yangluo2017	2	QT539	70.13
Yangluo2017	2	QT540	78.90
Yangluo2017	2	QT541	79.74
Yangluo2017	2	QT542	75.97
Yangluo2017	2	QT543	-
Yangluo2017	2	QT544	68.36

Yangluo2017	2	QT545	77.69
Yangluo2017	2	QT546	73.23
Yangluo2017	2	QT547	79.91
Yangluo2017	2	QT548	65.78
Yangluo2017	2	QT549	67.41
Yangluo2017	2	QT550	70.03
Yangluo2017	2	QT551	77.14
Yangluo2017	2	QT552	72.96
Yangluo2017	2	QT553	76.21
Yangluo2017	2	QT554	77.92
Yangluo2017	2	QT555	76.17
Yangluo2017	2	QT556	75.88
Yangluo2017	2	QT557	72.58
Yangluo2017	2	QT558	71.49
Yangluo2017	2	QT559	74.00
Yangluo2017	2	QT560	74.40
Yangluo2017	2	QT561	76.00
Yangluo2017	2	QT562	77.47
Yangluo2017	2	QT563	71.92
Yangluo2017	2	QT564	71.39
Yangluo2017	2	QT565	79.82
Yangluo2017	2	QT566	77.14
Yangluo2017	2	QT567	76.18
Yangluo2017	2	QT568	78.22
Yangluo2017	2	QT569	71.49
Yangluo2017	2	QT570	73.73
Yangluo2017	2	QT571	80.19
Yangluo2017	2	QT572	69.36
Yangluo2017	2	QT573	71.67
Yangluo2017	2	QT574	-
Yangluo2017	2	QT575	77.68
Yangluo2017	2	QT576	73.20
Yangluo2017	2	QT577	72.00
Yangluo2017	2	QT578	74.50
Yangluo2017	2	QT579	76.32
Yangluo2017	2	QT580	79.48
Yangluo2017	2	QT581	75.11
Yangluo2017	2	QT582	77.67
Yangluo2017	2	QT583	78.59
Yangluo2017	2	QT584	78.73
Yangluo2017	2	QT585	75.78
Yangluo2017	2	QT586	75.71
Yangluo2017	2	QT587	71.38

Yangluo2017	2	QT588	80.20
Yangluo2017	2	QT589	82.08
Yangluo2017	2	QT590	75.69
Yangluo2017	2	QT591	70.17
Yangluo2017	2	QT592	72.93
Yangluo2017	2	QT593	77.12
Yangluo2017	2	QT594	73.96
Yangluo2017	2	QT595	76.01
Yangluo2017	2	QT596	72.00
Yangluo2017	2	QT597	69.59
Yangluo2017	2	QT598	-
Yangluo2017	2	QT599	76.96
Yangluo2017	2	QT600	80.33
Yangluo2017	2	QT601	71.32
Yangluo2017	2	QT602	68.69
Yangluo2017	2	QT603	67.87
Yangluo2017	2	QT604	73.66
Yangluo2017	2	QT605	74.91
Yangluo2017	2	QT606	78.49
Yangluo2017	2	QT607	69.45
Yangluo2017	2	QT608	78.00
Yangluo2017	2	QT609	78.66
Yangluo2017	2	QT610	73.12
Yangluo2017	2	QT611	79.62
Yangluo2017	2	QT612	76.83
Yangluo2017	2	QT613	71.95
Yangluo2017	2	QT614	70.34
Yangluo2017	2	QT615	72.93
Yangluo2017	2	QT616	78.90
Yangluo2017	2	QT617	74.96
Yangluo2017	2	QT618	78.52
Yangluo2017	2	QT619	80.73
Yangluo2017	2	QT620	72.20
Yangluo2017	2	QT621	75.20
Yangluo2017	2	QT622	78.32
Yangluo2017	2	QT623	73.52
Yangluo2017	2	QT624	76.45
Yangluo2017	2	QT625	78.06
Yangluo2017	2	QT626	75.98
Yangluo2017	2	QT627	72.64
Yangluo2017	2	QT628	76.23
Yangluo2017	2	QT629	76.36
Yangluo2017	2	QT630	68.03

Yangluo2017	2	QT631	68.57
Yangluo2017	2	QT632	73.44
Yangluo2017	2	QT633	73.85
Yangluo2017	2	QT634	69.09
Yangluo2017	2	QT635	74.08
Yangluo2017	2	QT636	73.66
Yangluo2017	2	QT637	70.93
Yangluo2017	2	QT638	68.04
Yangluo2017	2	QT639	75.01
Yangluo2017	2	QT640	79.75
Yangluo2017	2	QT641	81.24
Yangluo2017	2	QT642	73.95
Yangluo2017	2	QT643	68.96
Yangluo2017	2	QT644	-
Yangluo2017	2	QT645	77.35
Yangluo2017	2	QT646	73.41
Yangluo2017	2	QT647	76.97
Yangluo2017	2	QT648	68.24
Yangluo2017	2	QT649	78.20
Yangluo2017	2	QT650	72.42
Yangluo2017	2	QT651	69.73
Yangluo2017	2	QT652	71.77
Yangluo2017	2	QT653	76.46
Yangluo2017	2	QT654	76.12
Yangluo2017	2	QT655	74.11
Yangluo2017	2	QT656	75.85
Yangluo2017	2	QT657	74.15
Yangluo2017	2	QT658	67.63
Yangluo2017	2	QT659	67.99
Yangluo2017	2	QT660	70.30
Yangluo2017	2	QT661	74.58
Yangluo2017	2	QT662	67.49
Yangluo2017	2	QT663	71.03
Yangluo2017	2	QT664	76.95
Yangluo2017	2	QT665	73.55
Yangluo2017	2	QT666	69.98
Yangluo2017	2	QT667	74.47
Yangluo2017	2	QT668	79.64
Yangluo2017	2	QT669	73.28
Yangluo2017	2	QT670	77.89
Yangluo2017	2	QT671	71.83
Yangluo2017	2	QT672	66.50
Yangluo2017	2	QT673	73.74

Yangluo2017	2	QT674	69.68
Yangluo2017	2	QT675	66.93
Yangluo2017	2	QT676	73.61
Yangluo2017	2	QT677	71.85
Yangluo2017	2	QT678	78.90
Yangluo2017	2	QT679	74.37
Yangluo2017	2	QT1980	69.76
Yangluo2017	2	QT1981	74.07
Yangluo2017	2	Xuzhou68-4	70.95
Yangluo2017	2	Yuanza 9102	78.98
Yangluo2017	3	QT487	78.54
Yangluo2017	3	QT488	72.24
Yangluo2017	3	QT489	74.75
Yangluo2017	3	QT490	74.73
Yangluo2017	3	QT491	70.79
Yangluo2017	3	QT492	75.53
Yangluo2017	3	QT493	78.39
Yangluo2017	3	QT494	75.62
Yangluo2017	3	QT495	80.76
Yangluo2017	3	QT496	77.37
Yangluo2017	3	QT497	76.03
Yangluo2017	3	QT498	78.35
Yangluo2017	3	QT499	71.17
Yangluo2017	3	QT500	70.83
Yangluo2017	3	QT501	78.23
Yangluo2017	3	QT502	70.45
Yangluo2017	3	QT503	71.26
Yangluo2017	3	QT504	70.06
Yangluo2017	3	QT505	68.51
Yangluo2017	3	QT506	79.51
Yangluo2017	3	QT507	76.72
Yangluo2017	3	QT508	71.30
Yangluo2017	3	QT509	71.69
Yangluo2017	3	QT510	76.56
Yangluo2017	3	QT511	79.39
Yangluo2017	3	QT512	80.04
Yangluo2017	3	QT513	74.31
Yangluo2017	3	QT514	78.92
Yangluo2017	3	QT515	74.55
Yangluo2017	3	QT516	74.55
Yangluo2017	3	QT517	76.21
Yangluo2017	3	QT518	79.30
Yangluo2017	3	QT519	67.18

Yangluo2017	3	QT520	69.05
Yangluo2017	3	QT521	73.03
Yangluo2017	3	QT522	80.21
Yangluo2017	3	QT523	67.02
Yangluo2017	3	QT524	76.14
Yangluo2017	3	QT525	77.95
Yangluo2017	3	QT526	73.12
Yangluo2017	3	QT527	71.49
Yangluo2017	3	QT528	68.39
Yangluo2017	3	QT529	81.28
Yangluo2017	3	QT530	79.38
Yangluo2017	3	QT531	75.44
Yangluo2017	3	QT532	76.53
Yangluo2017	3	QT533	72.55
Yangluo2017	3	QT534	79.48
Yangluo2017	3	QT535	65.55
Yangluo2017	3	QT536	72.90
Yangluo2017	3	QT537	75.86
Yangluo2017	3	QT538	70.63
Yangluo2017	3	QT539	68.78
Yangluo2017	3	QT540	76.09
Yangluo2017	3	QT541	78.28
Yangluo2017	3	QT542	76.44
Yangluo2017	3	QT543	-
Yangluo2017	3	QT544	68.48
Yangluo2017	3	QT545	75.73
Yangluo2017	3	QT546	74.11
Yangluo2017	3	QT547	76.70
Yangluo2017	3	QT548	66.67
Yangluo2017	3	QT549	68.47
Yangluo2017	3	QT550	69.29
Yangluo2017	3	QT551	74.17
Yangluo2017	3	QT552	70.14
Yangluo2017	3	QT553	74.94
Yangluo2017	3	QT554	77.52
Yangluo2017	3	QT555	74.79
Yangluo2017	3	QT556	72.97
Yangluo2017	3	QT557	68.65
Yangluo2017	3	QT558	71.39
Yangluo2017	3	QT559	74.32
Yangluo2017	3	QT560	75.55
Yangluo2017	3	QT561	76.46
Yangluo2017	3	QT562	74.17

Yangluo2017	3	QT563	68.63
Yangluo2017	3	QT564	70.46
Yangluo2017	3	QT565	78.37
Yangluo2017	3	QT566	74.40
Yangluo2017	3	QT567	76.96
Yangluo2017	3	QT568	75.40
Yangluo2017	3	QT569	72.16
Yangluo2017	3	QT570	72.91
Yangluo2017	3	QT571	82.02
Yangluo2017	3	QT572	68.60
Yangluo2017	3	QT573	69.80
Yangluo2017	3	QT574	-
Yangluo2017	3	QT575	77.47
Yangluo2017	3	QT576	71.74
Yangluo2017	3	QT577	71.46
Yangluo2017	3	QT578	74.57
Yangluo2017	3	QT579	76.31
Yangluo2017	3	QT580	77.41
Yangluo2017	3	QT581	76.22
Yangluo2017	3	QT582	77.57
Yangluo2017	3	QT583	75.38
Yangluo2017	3	QT584	81.48
Yangluo2017	3	QT585	75.71
Yangluo2017	3	QT586	74.00
Yangluo2017	3	QT587	70.05
Yangluo2017	3	QT588	77.10
Yangluo2017	3	QT589	80.37
Yangluo2017	3	QT590	72.58
Yangluo2017	3	QT591	70.40
Yangluo2017	3	QT592	73.46
Yangluo2017	3	QT593	78.79
Yangluo2017	3	QT594	71.07
Yangluo2017	3	QT595	74.19
Yangluo2017	3	QT596	72.72
Yangluo2017	3	QT597	71.69
Yangluo2017	3	QT598	-
Yangluo2017	3	QT599	74.46
Yangluo2017	3	QT600	76.74
Yangluo2017	3	QT601	69.44
Yangluo2017	3	QT602	69.99
Yangluo2017	3	QT603	68.13
Yangluo2017	3	QT604	74.20
Yangluo2017	3	QT605	74.90

Yangluo2017	3	QT606	80.75
Yangluo2017	3	QT607	70.18
Yangluo2017	3	QT608	78.52
Yangluo2017	3	QT609	77.36
Yangluo2017	3	QT610	73.53
Yangluo2017	3	QT611	77.57
Yangluo2017	3	QT612	79.62
Yangluo2017	3	QT613	73.89
Yangluo2017	3	QT614	71.01
Yangluo2017	3	QT615	74.37
Yangluo2017	3	QT616	76.62
Yangluo2017	3	QT617	71.10
Yangluo2017	3	QT618	77.92
Yangluo2017	3	QT619	79.05
Yangluo2017	3	QT620	72.02
Yangluo2017	3	QT621	74.27
Yangluo2017	3	QT622	75.35
Yangluo2017	3	QT623	71.26
Yangluo2017	3	QT624	78.02
Yangluo2017	3	QT625	74.36
Yangluo2017	3	QT626	76.86
Yangluo2017	3	QT627	71.98
Yangluo2017	3	QT628	77.83
Yangluo2017	3	QT629	74.30
Yangluo2017	3	QT630	69.65
Yangluo2017	3	QT631	67.87
Yangluo2017	3	QT632	75.16
Yangluo2017	3	QT633	74.53
Yangluo2017	3	QT634	68.83
Yangluo2017	3	QT635	73.48
Yangluo2017	3	QT636	73.12
Yangluo2017	3	QT637	68.58
Yangluo2017	3	QT638	69.51
Yangluo2017	3	QT639	72.79
Yangluo2017	3	QT640	81.33
Yangluo2017	3	QT641	79.14
Yangluo2017	3	QT642	72.51
Yangluo2017	3	QT643	66.33
Yangluo2017	3	QT644	-
Yangluo2017	3	QT645	75.94
Yangluo2017	3	QT646	73.28
Yangluo2017	3	QT647	75.78
Yangluo2017	3	QT648	68.76

Yangluo2017	3	QT649	80.20
Yangluo2017	3	QT650	69.87
Yangluo2017	3	QT651	70.27
Yangluo2017	3	QT652	73.21
Yangluo2017	3	QT653	74.53
Yangluo2017	3	QT654	77.12
Yangluo2017	3	QT655	75.98
Yangluo2017	3	QT656	74.90
Yangluo2017	3	QT657	75.72
Yangluo2017	3	QT658	67.54
Yangluo2017	3	QT659	69.62
Yangluo2017	3	QT660	72.03
Yangluo2017	3	QT661	72.95
Yangluo2017	3	QT662	67.32
Yangluo2017	3	QT663	68.20
Yangluo2017	3	QT664	74.84
Yangluo2017	3	QT665	71.70
Yangluo2017	3	QT666	67.02
Yangluo2017	3	QT667	76.08
Yangluo2017	3	QT668	79.04
Yangluo2017	3	QT669	75.84
Yangluo2017	3	QT670	74.01
Yangluo2017	3	QT671	70.63
Yangluo2017	3	QT672	63.41
Yangluo2017	3	QT673	72.38
Yangluo2017	3	QT674	71.56
Yangluo2017	3	QT675	66.55
Yangluo2017	3	QT676	71.37
Yangluo2017	3	QT677	70.70
Yangluo2017	3	QT678	78.11
Yangluo2017	3	QT679	74.72
Yangluo2017	3	QT1980	68.53
Yangluo2017	3	QT1981	70.52
Yangluo2017	3	Xuzhou68-4	69.86
Yangluo2017	3	Yuanza 9102	81.71
