# Supplementary material for: Discovery of genomic regions and candidate genes controlling shelling percentage using QTL‐seq approach in cultivated peanut (Arachis hypogaea L.)
Source: Plant Biotechnol J. 2019 Jan 30;17(7):1248–60. doi: 10.1111/pbi.13050 (PMC6576108; doi:10.1111/pbi.13050)
Supplement: Supplementary file 20 — Table S8 Phenotypic effect of the two major and stable QTLs for shelling percentage in the RIL population. [file PBI-17-1248-s016.pdf]

Table S8 Phenotypic effect of the two major and stable QTLs for shelling percentage in the RIL population.

Genotype	No. of RILs	Yangluo2017	Wuhan2016	Wuhan2015	Wuhan2014	Wuhan2013	Signifiance level
aabb	48	71.44 ±3.34[a]	75.71 ±3.45[a]	75.88 ±3.51[a]	75.45 ±3.68[a]	74.53 ±3.74[a]	0.1
AAbb	26	74.12 ±2.90[b]	78.29 ±2.39[b]	78.31 ±2.30[b]	77.88 ±3.05[b]	77.13 ±3.86[b]	
aaBB	23	75.46 ±3.30[b]	79.10 ±2.23[b]	78.89 ±1.70[b]	79.07 ±2.58[b]	77.43 ±2.92[b]	
AABB	35	76.92 ±2.68[c]	80.36 ±1.56[c]	80.43 ±1.47[c]	80.70 ±1.91[c]	79.44 ±2.07[c]	
aabb	48	71.44 ±3.34[A]	75.71 ±3.45[A]	75.88 ±3.51[A]	75.45 ±3.68[A]	74.53 ±3.74[A]	0.05
AAbb	26	74.12 ±2.90[B]	78.29 ±2.39[B]	78.31 ±2.30[B]	77.88 ±3.05[B]	77.13 ±3.86[B]	
aaBB	23	75.46 ±3.30[BC]	79.10 ±2.23[BC]	78.89 ±1.70[B]	79.07 ±2.58[B]	77.43 ±2.92[B]	
AABB	35	76.92 ±2.68[C]	80.36 ±1.56[C]	80.43 ±1.47[C]	80.70 ±1.91[C]	79.44 ±2.07[C]	

Genotype the genotype of RILs, AA: genotype of KASP marker Aradu_A09_66949737 from Yuanza 9102, aa: genotype of KASP marker Aradu_A09_66949737 from Xuzhou 68-4, BB: genotype of KASP markers Araip_B02_6155951, Araip_B02_6770282 and Araip_B02_6776001 from Yuanza 9102, bb: genotype of KASP markers Araip_B02_6155951, Araip_B02_6770282 and Araip_B02_6776001 from Xuzhou 68-4.

[a,b,c]Means followed by different letter are statistically different at $p<0.10$ based on ANOVA and Duncan multiple-comparison.

[A,B,C]Means followed by different letter are statistically different at $p<0.05$ based on ANOVA and Duncan multiple-comparison.
